# The Sucrose Synthase Gene Family in Chinese Pear (*Pyrus bretschneideri* Rehd.): Structure, Expression, and Evolution

**DOI:** 10.3390/molecules23051144

**Published:** 2018-05-11

**Authors:** Muhammad Abdullah, Yungpeng Cao, Xi Cheng, Dandan Meng, Yu Chen, Awais Shakoor, Junshan Gao, Yongping Cai

**Affiliations:** 1 School of Life Sciences, Anhui Agricultural University, Hefei 230036, China; abdullahpadana@hotmail.com (M.A.); xfcypeng@126.com (Y.C.); cxzp1114@163.com (X.C.); mdd5749@126.com (D.M.); jacyhao@163.com (Y.C.); gaojsh@ahau.edu.cn (J.G.); 2 School of Resources and Environment, Anhui Agricultural University, Hefei 230036, China; awais.shakoor22@gmail.com

**Keywords:** sucrose synthase, characteristics, phylogenetic analysis, RNA-seq data analysis, expression pattern

## Abstract

Sucrose synthase (SS) is a key enzyme involved in sucrose metabolism that is critical in plant growth and development, and particularly quality of the fruit. Sucrose synthase gene families have been identified and characterized in plants various plants such as tobacco, grape, rice, and *Arabidopsis*. However, there is still lack of detailed information about sucrose synthase gene in pear. In the present study, we performed a systematic analysis of the pear (*Pyrus bretschneideri* Rehd.) genome and reported 30 sucrose synthase genes. Subsequently, gene structure, phylogenetic relationship, chromosomal localization, gene duplications, promoter regions, collinearity, RNA-Seq data and qRT-PCR were conducted on these sucrose synthase genes. The transcript analysis revealed that 10 *PbSSs* genes (30%) were especially expressed in pear fruit development. Additionally, qRT-PCR analysis verified the RNA-seq data and shown that *PbSS30*, *PbSS24*, and *PbSS15* have a potential role in the pear fruit development stages. This study provides important insights into the evolution of sucrose synthase gene family in pear and will provide assistance for further investigation of sucrose synthase genes functions in the process of fruit development, fruit quality and resistance to environmental stresses.

## 1. Introduction

In higher plants, sucrose is an essential element of the life cycle. It is mainly produced by photosynthesis source tissues and transported to sink tissues where it serves as a carbon and energy source for the various metabolic pathways. The utilization of sucrose in plant cells requires its cleavage, which is performed by two key enzymes, sucrose synthase (UDP-glucose: d-fructose-2-glucosyltransferase, EC 2.4.1.13, EC 2.4.1.13) and sucrose cleavage invertase. Sucrose synthase (Sus) accelerates the reversible conversion of sucrose and UDP into UDP-glucose and fructose, whereas invertase (Inv) speeds up the cleavage of sucrose into glucose and fructose [1,2,3]. Sus and Inv are both involved in the energy source for phloem loading. In addition, Sus contributes to dispensing of carbon resources into several pathways that are essential for the metabolic and storage physiology of plant cell [4,5,6].

In recent studies, it has been acknowledged that sucrose acts as a signal to modulate a wide range of processes in plants by regulating the expression level of genes encoding enzymes, storage proteins and transformations [1,2,3], for example cell division [7], fruit development [3] seed germination [8], flowering induction [4], anthocyanin biosynthesis [5], vascular tissue differentiation [6], response to abiotic stresses [9,10] and the accumulation of storage products [7,8]. Thus, the study of sucrose metabolism is focal for understanding a myriad sides of plant physiology.

Previous studies reported that sucrose synthase is encoded by multiple small gene families, which have distinct and partially overlapping expression patterns. The identification of the genes encoding sucrose synthase is the first step for understanding their physiological roles and involvement in different metabolic processes. To date, with growing whole genome sequencing data more sucrose synthase gene families have been identified and subsequently characterized in plants. The number of sucrose synthase gene family members differs among the plant species. For example, the pea and maize genomes have three sucrose synthase genes [11,12] while six distinct genes were identified in *Arabidopsis*, rice, and *Lotus japonica* [2,13]. Eight sucrose synthase genes have been identified in diploid cotton genomes (*Gossypium arboreum* L. and *G. raimondii* Ulbr.), while fifteen members are found in the tetraploid cotton genome (*G. hirsutum* L.) [14]. *Arabidopsis* sucrose synthase genes contain three clearly distinct groups but partially overlapping expression pattern; genes functions is also distinct, according to studies from loss-of-function mutants [6,15,16].

Pear (*Pyrus. bretschneideri*) which can produce fruit with great commercial value, is grown in temperate regions. The accumulation of sugar content is an important factor affecting the taste and quality of fruit. The sucrose synthase gene family has shown its potential roles in sugar accumulation through the Sus enzyme activities and each member in sucrose synthase gene families may have a specific role in a given tissue or organ of the species [15,17]. The first step is the identification of the genes encoding sucrose synthase for understanding their physiological roles and connection in different metabolic process. Sucrose synthase gene families have been identified in many plant species including rice, *Arabidopsis*, tobacco, cotton, poplar, and peach but still have not been functionally characterized in *Pyrus* (*P. bretschneideri*). In the present study, we report the identification of the sucrose synthase family genes using pear (*Pyrus. bretschneideri*) sequences. To further expand the knowledge of sucrose synthase gene family, we identified 30 *PbSS* genes in pear (*P. bretschneideri*). The gene structure, evolutionary relationship, microcollinearity, divergence expression were conducted on pear sucrose synthase gene family. In addition, expression profiles of candidate genes during different fruit development stages were analyzed by quantitative real-time reverse transcription PCR (qRT-PCR) and also describe the dynamics of the corresponding transcript levels during different fruit development stages. Our results provided precise information of sucrose synthase gene family that will be supportive for ongoing research on this important gene family in fruit plants, predominantly in the pear.

## 2. Results

### 2.1. Data Mining, Identification and Molecular Characterization of Sucrose Synthase Gene Family in Chinese Pear

In order to obtain sucrose synthase proteins in pear genome, *Arabidopsis* sucrose synthase proteins were extracted from previous studies (http://peargenome.njau.edu.cn/). We performed BLAST searches using *Arabidopsis* sucrose synthase protein sequences against the pear genome and finally retained 30 *PbSS* genes. Additionally, all *PbSS* protein sequences were verified with SMART and Pfam databases, and named from *PbSS1* to *PbSS30* based on chromosomes information (Supplementary Table S1). All the *PbSS* genes contained at least one SS domain, and some *PbSS* genes have two or three SS domains, such as *PbSS27*, *PbSS26*, *PbSS20* (contains two domains) and *PbSS13* (contains three domains). At the same time, we found that the *PbSS* genes in the same subfamily have similar domain distributions and compositions, suggesting a similar evolutionary history in these subfamilies. ExPASy server was used to calculate the molecular characteristics of all *PbSS* genes including the protein length, molecular weight and isoelectric point.

The amino acid lengths of these *PbSS* proteins ranged from 63 to 1142, with an average of 758. Similarly, the molecular weight of these *PbSS* genes ranged from 7543.7 to 129,417.5 kDa with estimated Isoelectric point (IP) ranging from 4.69 to 8.66. The predicted molecular features of these *PbSS* genes were similar to previously characterized sucrose synthase genes from other plant species. Pear genome annotation (http://peargenome.njau.edu.cn/) was used to determine the location of all *PbSS* genes on the Pear chromosomes, length of the chromosomes, gene number, and start/end sites. All *PbSS* genes physical map was drawn, except that gene located on scaffold according to pear genome annotation. As a result, a total of 30 *PbSS* genes, 20 genes located on chromosomes and 10 genes were located on the scaffold. Among 20 *PbSS* genes, maximum number of *PbSS* genes (20%) located on the 15th chromosome, while chromosomes 2 and 9 contained three *PbSS* genes (15%). Chromosome 3, 13, and 17 comprise two *PbSS* genes and remaining chromosomes 5, 6, 8 and 11 only have one *PbSS* gene each.

### 2.2. Gene Structure and Conserved Motif Organization in the Pear Sucrose Synthase Gene Family

**Figure 1:**
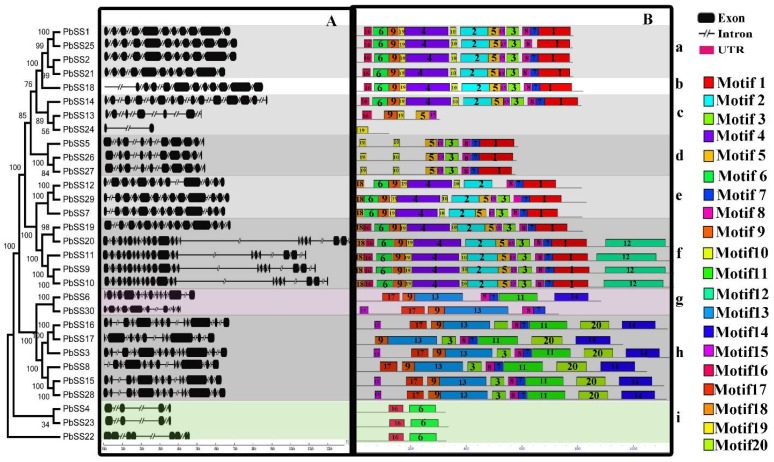
Gene structure and distribution of conserved motif of *PbSSs* genes in Chinese pear. (**A**) Phylogenetic relationship and gene structure of pear *PbSSs* genes. (**B**) Conserved motifs located on each gene with the relative combined *p*-value.

To determine the further genesis of sucrose synthase gene family in Chinese pear, we performed exon/introns structure analysis. In order to investigate the structural diversity of *PbSS* genes, genomic sequences and CDS sequences were compared. As shown in Figure 1, most *PbSS* genes were clustered in the same group having a similar number of exons and introns. For example, four *PbSS* genes belonging to clade “a” having 11/10 exon/intron and three members within clade “e” contain 13/12 exons/introns. Additionally, the number of exons ranged from 2 to 20, clade “f” containing the highest number of exons (18 to 20), while group “i, c” members have only 2 to 4 exons, respectively (Figure 1). These results deduced that exon/intron loss or gain occurred during the evolution of pear sucrose synthase gene family.

In order to analyze the sequence features of PbSS genes, the conserved motifs were predicted using the MEME online web server. Finally, twenty motifs were identified in each comparison and named as motif 1 to 20. As shown in Figure 1, the majority of PbSS genes contained several motifs: 1, 2, 4, 5 and 6. For example, clade “i” contained only two motifs, 6 and 16, while other clades contained several motifs. Most of the closely related PbSS genes had the same motif compositions, suggesting that there are some functional similarities of PbSS genes within the same subfamily. Additionally, we observed that some subfamily specific motif, which might be a critical role in subfamily specific functions. For example, motif 8 and 10 in clade “d” and motif 12 in clade “f”. Furthermore, some motifs were found almost in every subfamily, such as motifs 19, 6 and 1, suggesting these motifs might be important to the function of PbSS genes. The similarity in motif composition and intron/exon structure of PbSS genes within the same group supported the phylogenetic analysis of sucrose synthase gene family, while the difference between the different groups specified their function was diversified.

### 2.3. Phylogenetic Analysis of the Pear Sucrose Synthase Gene Family

**Figure 2:**
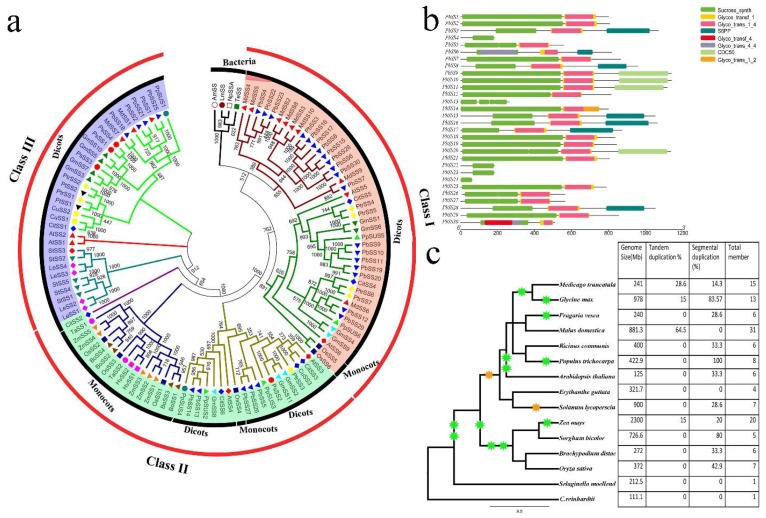
Phylogenetic relationships and domain structures of the sucrose synthase genes. (**a**) Phylogenetic analysis of pear sucrose synthase genes with other ten species (Supplementary Table S2) (**b**) Domain structures of the sucrose synthase proteins. Sucrose_synth indicated sucrose synthase domains. (**c**) The phylogenetic tree was generated by using NCBI database (https://www.ncbi.nlm.nih.gov/Taxonomy/CommonTree/wwwcmt.cgi) and presented the history of whole genome duplication of fifteen species. Green and dark orange specify whole genome duplication and triplication, respectively.

To investigate the evolutionary relationships of pear sucrose synthase gene family and other plant species, a phylogenetic tree was constructed after multiple sequence alignment of the full-length sequence of sucrose synthase genes from 94 dicot sequences, 19 monocot sequences and four bacterial sequences using ClustalX (Supplementary Table S2). All the sucrose synthase genes were divided into three groups based on previous analyses with which our results were consistent. The phylogenetic analysis of sucrose synthase family genes exposed relatively recent duplications and deep evolutionary origin. Of these, plant 94 sucrose synthase genes formed a monophyletic group, while all bacterial sucrose synthase genes were clustered into the same group, designating that all land plant sucrose synthase genes originated from an ancestral type [18]. Plant 94 sucrose synthase genes were clustered into three subgroups designated as class I, II and class III, respectively [8,10,11,12] (Figure 2a). Additionally, class I and II could be further classified into two distinct subgroups, one specific for monocot sucrose synthase genes and another for dicot sucrose synthase genes, respectively. The phylogenetic analysis proposed that mostly plant sucrose synthase genes duplication event occurred gave rise to sucrose synthase gene of class I and III before the divergence of monocot/dicot. Nineteen pear sucrose synthase genes were distributed in the dicot branch of class I. Similarly class II and III dicot subgroup consist of five pear sucrose synthase genes. Although the pear sucrose synthase genes shared high sequence similarities, phylogenetic analysis exposed that diversification occurred within this family. These results signifying discrete evolutionary histories and diverse biological roles of sucrose synthase gene family members in pear.

### 2.4. Expansion Patterns and Syntenic Analysis of the Pear Sucrose Synthase Genes

**Figure 3:**
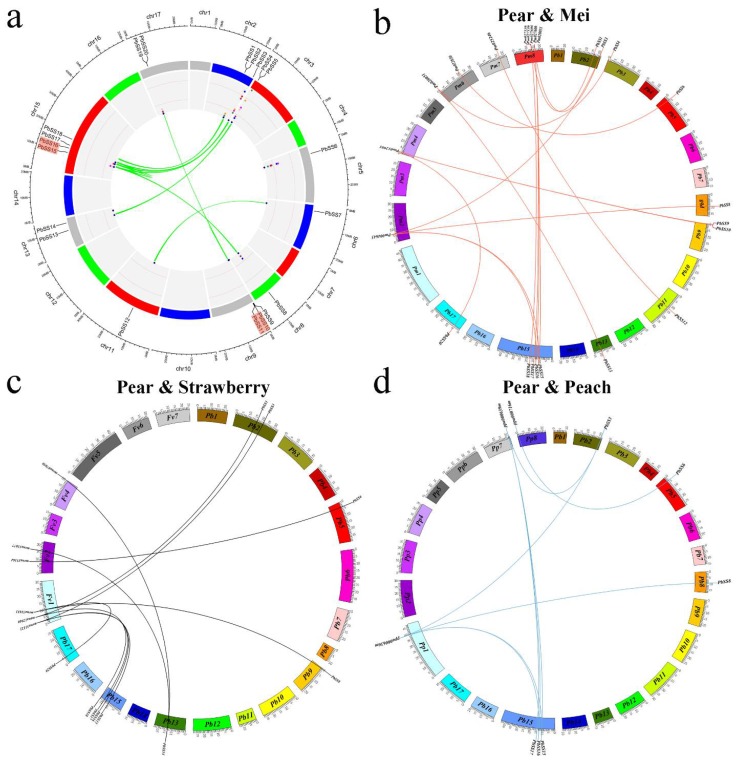
Chromosomal location and microsynteny regions of *PbSSs* genes. (**a**) Chromosomal location of all *PbSSs* genes and blue line represent segmental duplication and red highlighted *PbSSs* genes IDs represent tandem duplication. (**b**) Synteny analyses of *PbSSs* genes between *Pyrus bretschneideri* and *Prunus mume*. (**c**) Synteny analyses of *PbSSs* genes between *Pyrus bretschneideri* and *Fragaria vesca.* (**d**) Synteny analyses of *PbSSs* genes between *Pyrus bretschneideri* and *Prunus persica*.

Gene duplication (segmental and tandem duplication) and divergence are known to be important in gene family expansions and the evolution of novel functions [19]. Chinese pear (*Pyrus bretschneideri* Rehd.) shared an ancient whole-genome duplication (WGD) event that took place about 140 million years ago [20]. In order to investigate the impact of duplication on the pear sucrose synthase gene family, we examined the tandem and segmental duplications within the pear genome by using MCScanX and visualized by using circos software (Figure 3a). Finally, we identified 11 segmental duplication pairs and two tandem duplication pairs in pear sucrose synthase genes (Figure 3a). The segmental duplications genes on different chromosomes were indicated with green lines as shown in Figure 3a.

To gain further insights into the origin and evolutionary dynamics of pear sucrose synthase genes, microsynteny analysis was performed between the four species, including Chinese pear, peach, mei, and strawberry, respectively. In total, we identified 17, nine, and 11 orthologous gene pair in the cross of pear/mei, pear/peach, and pear/strawberry, respectively. Interestingly, we found that a single gene of Mei, peach, and strawberry syntenically corresponded to multiple genes of pear such as *Pm004643*-*PbSS3*/*PbSS8*/*PbSS15*/*PbSS16*, *mrna31653-PbSS9*/*PbSS20*, *Pp00036m*-*PbSS3*/*PbSS8*/*PbSS15*/*PbSS16* (Figure 3b–d) signifying that preferential gene duplication occurred in certain syntenic locations during pear genome evolution. Subsequently, some collinear gene pairs between pear and mei while could not identify pear and peach, pear and strawberry such as *PbSS4/Pm020051*, *PbSS13/Pm020051*, *PbSS10/Pm012991*, and *PbSS12/Pm025246*, suggested that these orthologous gene pairs were developed after peach and strawberry diverged from the common ancestor of pear and mei. Some collinear genes identified among pear/strawberry and pear/mei while were not found in pear/peach (Figure 3b–d).

**Figure 4:**
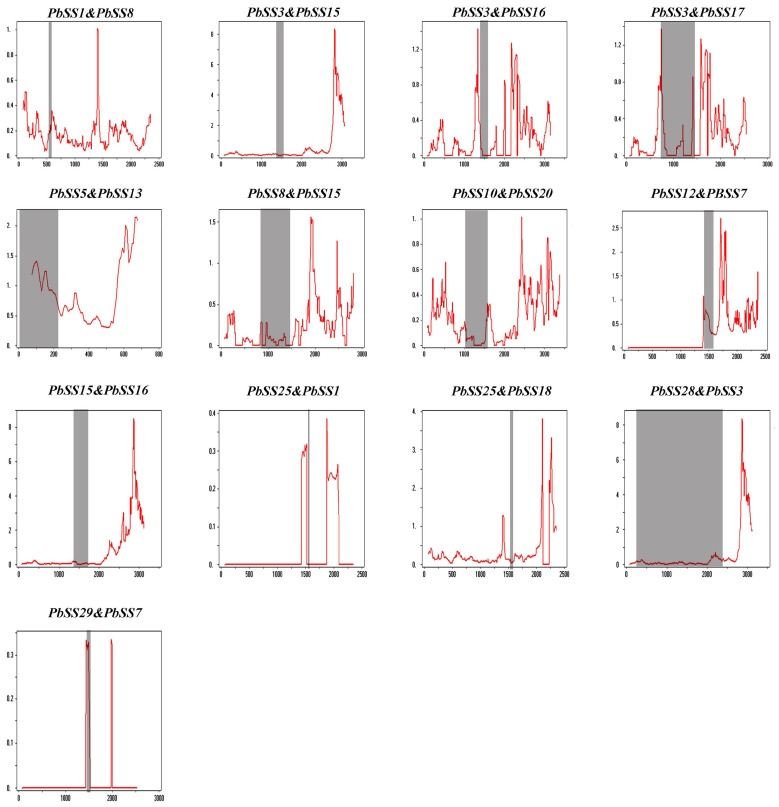
Sliding window plots of all duplicated *PbSSs* genes in pear. The window size is 150 bp, and the step size is 9 bp. The X-axis indicated the synonymous distance within each gene.

In general, we used Ks-values for estimating evolutionary data of duplication events (segmental duplication or whole genome duplication). Previous studies reported that the pear genome was created by two rounds of ancient whole-genome duplication events nearly at 140 million years ago (Ks ~ 1.5–1.8) and a recent whole-genome duplication approximately at 30–45 million years ago (Ks ~ 0.15–0.3) [21,22]. Subsequently, Ks values were calculated to analyze the segmental duplication events or whole genome duplication in the sucrose synthase gene family (Supplementary Table S3). We calculated Ks values of duplicated gene pairs, and the Ks mean value was 0.348731. These results suggested that duplications of sucrose synthase gene family may have been derived from the recent whole-genome duplication (Ks ~ 0.15–0.3). In addition, the Ka/Ks values are widely used to represent the gene selection pressure and evolution rate [23]. Ka/Ks > 1 suggests positive selection with accelerated evolution, Ka/Ks < 1 indicates purifying selection with the functional constraint and Ka/Ks = 1 represents that the genes are drifting naturally. We found that pear sucrose synthase gene family paralogs Ka/Ks ratios were less than 1, suggesting that their purifying/negative selection. Sliding Window analysis often used for detecting high Ka/Ks values that may represent high selection pressure at a single amino acid site or gene region. In order to investigate this in more depth, the delineated regions of diversifying and purifying selection in the sucrose synthase gene family, sliding window analysis of the Ka/Ks values between each paralog was performed. The sucrose synthase domain displayed strong purifying selection compared with the whole gene regions, as displayed in Figure 4. It was obvious that the sucrose synthase genes go through a strong purifying selection, especially the sucrose synthase domain in the pear. At the same interval, some parts of protein-coding genes undergo positive selection, suggesting the generation of novel gene function. In general, strong evolutionary constraints were involved in sucrose synthase gene evolution, which may contribute to the stability of gene function.

### 2.5. cis-Acting Element Analysis

**Figure 5:**
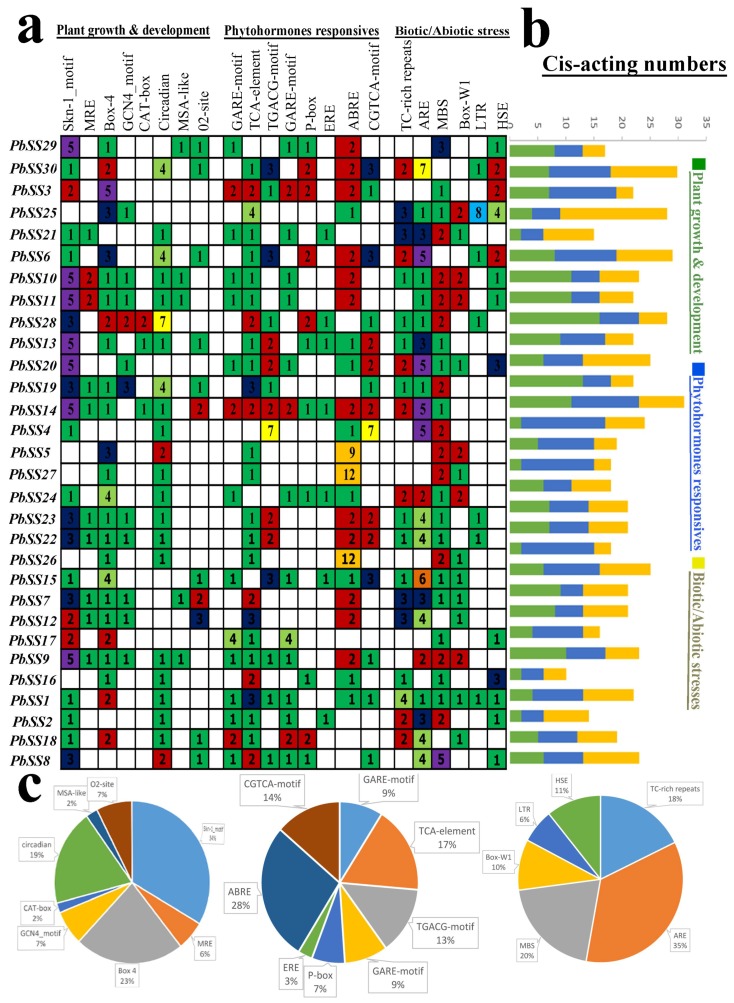
Investigation of *cis*-acting element numbers in all sucrose synthase genes of pear. (**a**) The different colors and numbers of the grid indicated the numbers of different promoter elements in these sucrose synthase genes. (**b**) The different colored histogram represented the sum of the *cis*-acting elements in each category. (**c**) Pie charts of different sizes indicated the ratio of each promoter element in each category, respectively.

Plant transcriptional mechanism contains two complimentary regulatory components: (1) *cis*-acting elements (2) *trans*-acting elements. *trans*-Acting factors are transcription factors or other DNA-binding proteins that bind to specific sequences in the *cis*-acting elements in order to increase or suppress the transcription of a given gene. *cis*-Acting elements are the DNA sequences in the coding or non-coding regions of the genome. *cis*-Regulatory elements play critical roles in regulatory networks control, including multi stimulus responsive genes, and determining the stress-responsive expression patterns or tissue-specific of genes were closely correlated with *cis*-elements in their promoter regions. In this study, we classified *cis*-acting elements into three classes, including biotic/abiotic stress responses, plant growth and development, and phytohormones responses in the promoter regions using the PlantCARE database. In the first category (growth and development), *cis*-acting elements extensively located in the promoter regions, containing Skn-1-motif and GCN4_motif that are essential for endosperm expression, CAT-box required for meristem expression, MRE and Box 4 for light responsiveness, circadian involved in circadian control, O_2_-site for zein metabolism regulation. We recognized the Skn-1-motifs covered the largest portion (34%) of the first category among these *cis*-acting elements, followed by Box-4 (23%) that are responsible for plant growth in response to light (Figure 5c). In the second category (phytohormone responsive), the TGA-element involved in auxin-response, GARE-motif and P-box for gibberellin-responsive elements, ERE for ethylene responsive ones. We detected the ABRE *cis*-acting elements related to ABA was the most common motif in the second category, 28% of the scanned hormone responsive motifs, followed by TCA-element that related to salicylic acid responsiveness. In the third category (biotic/abiotic stresses), we also identified a series of stresses-related elements, such as HSE involved in heat stress, ARE for anaerobic induction, Box-W1 for fungal elicitors, GC-motif for anoxia, and TC-rich repeats involved in stress responses. These results indicate that sucrose synthase gene family members have the potential for improving abiotic stress responses and might respond to abiotic stress.

### 2.6. Gene Expression of Sucrose Synthase Genes during Different Fruit Development Stages

**Figure 6:**
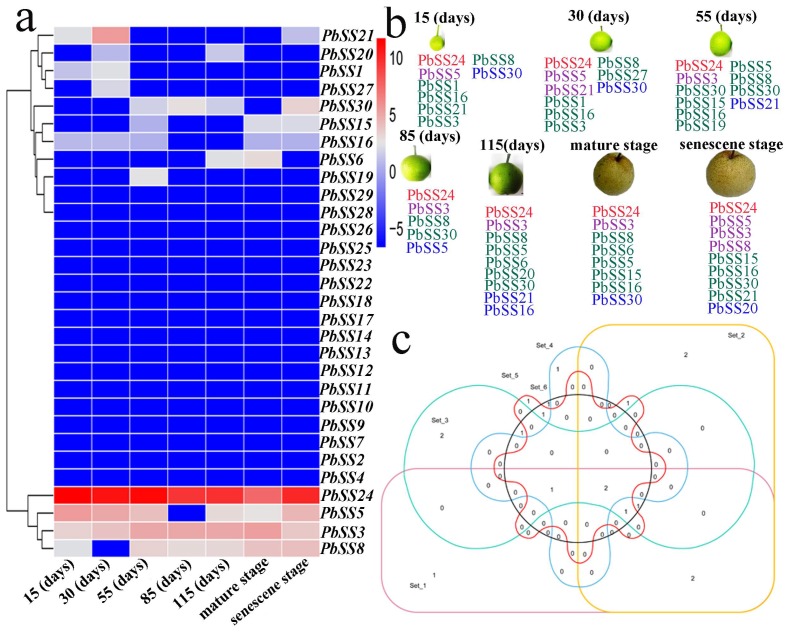
Expression pattern of *PbSSs* genes. (**a**) Organ-specific expression pattern of *PbSSs* genes in seven fruit development stages: 15, 30, 55, 85, 115 (DAF), mature stage and senescence stage. Blue and red indicated lower and higher transcript abundance, respectively. (**b**) Highly expressed *PbSSs* genes in pear fruit. According to the previous studies [24], blue, green, violet red, red indicated low (1–6.8 FPKM), mid-low (6.8–17.5 FPKM), mid-high (17.5–44.7 FPKM), and high (44.7–17,092 FPKM) expression, respectively. (**c**) Venn diagram of *PbSSs* genes in different fruit development stages.

**Figure 7:**
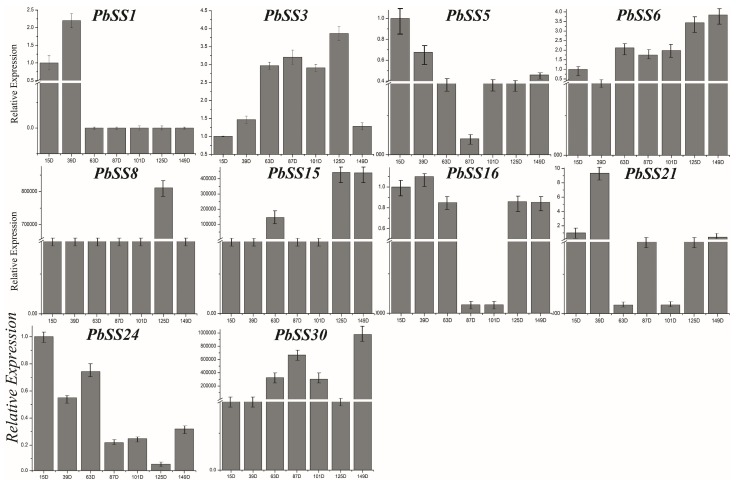
Expression patterns of *PbSSs* genes during different development stages, including 15, 39, 63, 22, 87, 101, 125 and 149 (DAF) as determined by qRT-PCR experiment. Mean values and standard deviations (SDs) indicated by error bars. ** significant difference (*p* < 0.01), * significant difference at *p* < 0.05.

For better understanding the dynamic gene expression profile of pear sucrose synthase gene family members, we carried out the analysis of gene expression patterns during seven fruit development stages (15D, 30D, 55D, 85D, 115D, mature stage, and senescence stage). The expression profile presented distinct stage-specific expression pattern for the *PbSS* genes, and further divided into three groups based on the expression pattern in seven developmental stages (Figure 6a). In the 1st group, four genes (*PbSS*24, *PbSS5*, *PbSS*3, and *PbSS*8) were widely expressed in all fruit development stages, indicating that these *PbSS* genes may have potential roles in the fruit development. Out of 30 genes, seventeen *PbSS* genes were classified into the second group, basically they were not expressed in these fruit development stages. In the 3rd group, the remaining eight genes showed similar lower expression in these fruit development stages but some genes expression specific to fruit development stages. We also observed that all homologous pairs have same expression pattern, such as *PbSS4* not expressed in all these development stages, while the expression pattern of its paralog (*PbSS23* and *PbSS22*) had also same expression pattern in all these fruit development stages, etc. (Figure 5b,c). To validate the authenticity of RNA-seq data, we selected 10 genes to investigate the expression pattern in seven fruit development stages. The qRT-PCR results were consistent with RNA-seq data and presented that these genes exhibited diverse expression patterns at 15, 39, 63, 87, 101, 125, and 149 days after blooming (Figure 7). The expression pattern of *PbSS*1, *PbSS*3, *PbSS*5, and *PbSS*16 was significantly increased at 39 DAF, suggesting that these genes may be involved in fruit enlargement stage. Similarly, *PbSS*21 and *PbSS*24 showed the expression pattern similar to RNA-Seq data, such as *PbSS21* expression was high at 39 DAF and mature stage. The results revealed that four genes (*PbSS*1, *PbSS*3, *PbSS*5, and *PbSS*16) exhibited similar expression patterns at 39 DAF, while all other genes exhibited variable expression patterns.

## 3. Discussion

Although the sucrose synthase gene family has been identified in various plants, such as citrus [3], tobacco [6], grape [25], populus [17] and peach [26], neither its function nor any comprehensive analysis of pear sucrose synthase gene family have been reported. In this study, we comprehensively analyzed the pear sucrose synthase gene family, including gene structure, analyses of phylogeny, promoter regions, chromosome localization, gene duplication, sequence features and expression pattern. A total of 30 sucrose synthase genes were identified from pear genome. The number of sucrose synthase genes in pear is thus higher than tobacco (14) [6], citrus (six) [3], grape (five) [25], *Arabidopsis* (six) [15], populus (seven) [17] and peach (six) [26]; these results showed that the sucrose synthase genes in different plants have been expanded to different degrees. Remarkably, the pear genome is larger in size (512 Mb) than peach (225 Mb) and grape (450), while smaller than the tobacco genome size (4.5 GB). These results suggested that the sucrose synthase gene family members may not be directly related to the genome size in different plants (Figure 2c).

In the plant evolution, gene duplication has played a very important role in the expansion of gene families as well as in generating novel genes. In order to gain further insights of the evolutionary history of a sucrose synthase gene family, we analyzed the tandem and segmental duplication in pear. In this study, two sucrose synthase genes of pear were located in tandem and 11 pear sucrose synthase genes were located in segmental duplication regions of pear chromosomes. These results imply that segmental duplications were the main driving forces for the expansion of pear sucrose synthase gene family members. Tandem duplication usually occurred in the rapidly evolving and large gene family, such as *NBS-LRR* gene family, while segmental duplication often occurred in the slowly evolving gene family, such as *MYB* gene family [27]. In addition, 10 *PbSS* genes could not be mapped on any chromosomes, which might be due to a high level of heterozygosity or the quality of pear genome. The genome replication and whole-genome duplication have occurred five billion years ago in Rosaceae species due to the number of chromosomes increased from nine to 17, which may have resulted in a large number of pear sucrose synthase genes as compared to other plants [28]. The *PbSS* duplication pair Ka/Ks ratios indicated that these genes have undergone purifying selection, while some parts of the CDS of *PbSSs* underwent positive selection, suggesting that new gene functions might have been acquired.

We conducted interspecific microsynteny analysis to identify the number of orthology genes, and maximum orthology was found between pear and yang mei (17) followed by strawberry (11) and peach (nine). These results were consistent with previous studies, and we can conclude that the divergence of yang mei, pear and peach occurred after the divergence of strawberry from the common ancestor of yang mei, pear, and peach [22,29,30]. These results offer a novel resource for the evolution study of sucrose synthase gene family among different species.

The RNA-Seq data expression profiles in different fruit development stages will contribute to study the tissue-specific and dynamic expression of sucrose synthase genes in pear. Although the detailed expression profiles of sucrose synthase genes have been studied in other plant species, such as *Arabidopsis* [13], rubber tree [9], and cotton [6], the possible role of pear sucrose synthase genes is still unclear. We presented the gene expression patterns of all sucrose synthase genes in pear. Among them, 30% sucrose synthase genes (such as *PbSS5*, *PbSS3*, and *PbSS24*) were highly expressed in seven fruit development stages, indicating that these genes have a potential role in the processes of fruit development in pear. The expression of several sucrose synthase genes was not observed in any fruit development stage, suggesting that they might be pseudogenes or might be expressed in other tissue-specific or under specific conditions. Previous studies reported that sucrose synthase genes play potential roles in the regulation of flowering, such as *vitis vinifera *VvSS4** [25] and *citrus paradise CitSus4* [3]. In the present study, we identified some *cis*-acting elements related to flowering in the *PbSS* genes promoter regions, such as circadian for circadian control elements, and GCN4_motif and Skn-1-motif required for endosperm expression. In the plant, many stress-related genes could generate stress responses, which were mediated and regulated by a variety of signaling pathways. In the present study, we identified a series of *cis*-acting elements (such as ERE, ABRE, HSF, LTR, ARE, and MBS) in the promoter regions of *PbSS* genes. Among these genes, at least one of the abiotic stress *cis*-elements were contained, implying that these genes might be contributing to responding the abiotic and biotic stresses. To better understand the function of sucrose synthase genes during different development stages of fruit. We carried out the qRT-PCR under different stages, including 15, 39, 63, 22, 87, 101, 125 and 149 days after flowering. We observed that the members of sucrose synthase gene family presented significantly differential expression profiles under different development stages. The pear sucrose synthase duplicate genes from recent whole-genome duplication have same expression patterns during different fruit development stages. This study about sucrose synthase genes will be helpful for improving resistant and fruit yield and quality of pear in the future.

In summary, the sequence of complete Chinese pear (*Pyrus bretschneideri*) genome provides the plant biology community with a wealth of new information for functional genomics. Benefitting from this genome sequence, a systematic analysis was performed including phylogenetic relationship, chromosomal location, gene structure, conserved motifs, microsynteny, promoter region, expression profile, and sliding window and qRT-PCR analysis of ten genes under different fruit development stages. A total of 30 sucrose synthase family genes were identified and divided into three groups according to phylogenetic analysis. Gene duplication of sucrose synthase family in pear indicated that segmental duplication contributed to the expansion of sucrose synthase genes in pear. Our experimental finding will be helpful for understanding the molecular basis of sucrose synthase genes in pear and these results can be used to engineer the pear fruit with enhancing stress resistance.

## 4. Materials and Methods

### 4.1. Plant Material

Forty-year-old pear *Pyrus bretschneideri* (var. Dangshan Su) plants from the High Technology Agricultural Research Park of Anhui Agricultural University (Hefei, Anhui, China) were used in this study. The pear samples were collected from similar developmental periods of plants grown towards the middle southern direction in April 2017. Healthy and uniform fruits (40) were collected on 19 April 15 days after flowering (DAF), 14 May (39 DAF), 30 May (63 DAF), 22 June (87 DAF), 18 July (101 DAF), 2 August (125 DAF) and 29 August (149 DAF) in 2017, respectively.

### 4.2. Identification of Sucrose Synthase Genes in Pear

To annotate and identify the sucrose synthase genes in pear genome, we used two different approaches. In the first approach, the known sequences of sucrose synthase genes from the TAIR database were used as queries to search for potential sucrose synthase genes in pear genome database by using BLASTP program (e-value < 1 × 10^−5^) (http://peargenome.njau.edu.cn/). In the second approach, the HMMER 3.0 software was used to identify for sucrose synthase genes in the pear genome, using HMM (hidden Markov model) profile of sucrose synthase domain (PF00862) from Pfam database as queries (e-value < 1 × 10^−3^) (Mistry et al., 2013). Subsequently, each putative BBX gene was further examined the presence of B-BOX domain by submitting them to SMART database (http://smart.embl-heidelberg.de/smart/set_mode.cgi?NORMAL=1) [31], Pfam (http://pfam.xfam.org/) [32] and InterProScan (http://www.ebi.ac.uk/interpro/ search/sequence-search) [33], respectively. The ExPASY website (ProtParam available online https://web.expasy.org/protparam/) was used to calculate the isoelectric point (pI) and molecular weight of all *PbSS* genes.

### 4.3. Sucrose Synthase Family Genes Physical Localization and Gene Duplications

To identify chromosomal location of all sucrose synthase genes, genome annotation data was collected and mapped with MapInspect software. Gene duplication pattern of sucrose synthase genes (*Pyrus bretschneideri*) was analyzed by using MCScanX software and BLASTP (1 × 10^−10^, identity > 80%) [34].

### 4.4. Phylogenetic Analysis, Motif Prediction and Gene Structure Analysis

The full-length sequence of sucrose synthase proteins in *Arabidopsis* was downloaded from the TAIR database, whereas the full-length sequence of sucrose synthase proteins from other ten species were downloaded from NCBI (Supplementary Table S2). A multiple alignment analysis of sucrose synthase family protein from 12 species performed with CLUSTAL_X software [35] and phylogenetic tree generated with MEGA 5.1 software using the neighbor-joining (NJ) method with bootstrap analysis (1000 replicates) [36]. The sucrose synthase family genes conserved motif was generated by using MEME online program (MEME, http://meme.sdsc.edu/meme/) [37]. All sucrose synthase genes GFF3 information file was used to determine gene structure by using GSDS website (http://gsds.cbi.pku.edu.cn/) [38].

### 4.5. Microsynteny Analysis and cis-Acting Element Analysis

Multiple Collinearity Scan toolkit (MCSscanX) was used to obtain the microsynteny between these four species *Pyrus bretschneideri*, *Fragaria vesca*, *Prunus persica*, and *Prunus mume*. To analyze the *cis*-acting elements, the Perl script was used to obtain the 1500 bp of genomic DNA sequence upstream of the initiation code (ATG). The PlantCARE software (http://bioinformatics.psb.ugent.be/webtools/plantcare/html/) was used to determine the presence of different *cis*-acting elements [39].

### 4.6. Calculation of Non-Synonymous (Ka) to Synonymous (Ks) Substitutions

The Ka (non-synonymous substitution rate)/Ks (synonymous substitution rate), Ka and Ks of duplicate gene pairs were calculated by using the DnaSP5.0 software based on a previous paper [21,40].

### 4.7. RNA-Seq Data Analysis

Public *P. bretschneideri* RNA-seq data were obtained from project number PRJNA309745 the NCBI database for subsequent expression analysis. The fastx_ toolkit was used to delete low quality base-calls (Q < 20). The clean reads after deleted/remove the low-quality base-calls (Q < 20) mapped to the reference genome using tophat2 software with default parameters and assembled using Cufflinks software [41,42] At the same time, the Cufflinks software was also used to detect the differentially expressed genes [42] The expression profiles were visualized using R software with our R script.

### 4.8. RNA Extraction, cDNA Synthesis and Quantitative Real-Time PCR (qRT-PCR)

Total RNA was extracted according to the manufacturer instructions of Tiangen (Beijing, China) plant RNA extraction kit. We synthesized first strand cDNA using gDNA Eraser (Takara, Japan) and each reaction used 1 μg of RNA. We designed and checked specific primer using Beacon Designer 7 software. The pear tubulin gene (forward primer: 5′-AGAACAAGAACTCGTCCTAC-3′; reverse primer: 5′-GAACTGCTCGCTCACTCTCC-3′) was used as reference gene [9]. The qRT-PCR was performed as follows: 98 °C for 2 min, 98 °C for 10 s, 60 °C for 10 s, and 40 cycles of 68 °C for 30 s, 95 °C for 15 s. The specificity of the reactions was verified by melting curve analysis. The 2^−ΔΔCT^ method was selected to calculate the relative gene expression levels [43].

## Supplementary Materials

Supplementary materials will be available online at http://www.mdpi.com/1420-3049/23/5/1144/s1. Table S1: The detailed information of PbSSs genes from Chinese pear. Table S2: List of sucrose synthase gene sequences used in this study. Table S3: The Ka, Ks and Ka/Ks values of PbSSs duplication gene pairs.

## Abbreviations

PbSSs	Pear Sucrose Synthase genes
Sus	Sucrose Synthase
Inv	Invertase cleavage
DAF	Days after flowering
Ka	Nonsynonymous
Ks	Synonymous
Mya	Million years
MEME	Multiple Em for Motif Elicitation
NJ	Neighbor-joining
qRT-PCR	Real-time PCR
Mb	Megabyte
Gb	Gigabyte
UDP	Uridine diphosphate
gDNA	Genomic DNA
CDS	Coding sequence
MW	Molecular weight
pI	Isoelectric point
