# Supplementary material for: The Sucrose Synthase Gene Family in Chinese Pear (Pyrus bretschneideri Rehd.): Structure, Expression, and Evolution
Source: Molecules. 2018 May 11;23(5):1144. doi: 10.3390/molecules23051144 (PMC6100409; doi:10.3390/molecules23051144)
Supplement: Supplementary file 1 [file molecules-23-01144-s001.pdf]

The Sucrose Synthase Gene Family in Chinese Pear (*Pyrus bretschneideri* Rehd.): Structure, Expression, and Evolution

Muhammad Abdullah [1],Yungpeng Cao [1], Xi Cheng [1], Dandan Meng [1], Yu Chen [1], Awais Shakoor [2], Junshan Gao [1] and Yongping Cai [1,*]

[1] School of Life Sciences, Anhui Agricultural University, Hefei 230036, China; abdullahpadana@hotmail.com (M.A.); xfcypeng@126.com (Y.C.); cxzp1114@163.com (X.C.); mdd5749@126.com (D.M.); jacyhao@163.com (Y.C.); gaojsh@ahau.edu.cn (J.G.)

[2] School of Resources and Environment, Anhui Agricultural University, Hefei 230036, China; awais.shakoor22@gmail.com

* Correspondence: swkx12@ahau.edu.cn; Tel.: +86-551-6578-6137; Fax: +86-551-6578-6340

Table S1. The detailed information of PbSSs genes from Chinese pear.

Gene Name	Gene ID	Chromosome	Start	End	Strand	Length (bp)	Molecular weight	Theoretical PI
PbSS1	Pbr035996.1	Chr2	16772840	16776299	+	801	91910.48	5.89
PbSS2	Pbr035997.1	Chr2	16782598	16786033	+	807	92685.32	5.92
PbSS3	Pbr003107.1	Chr2	21529677	21535125	+	1072	120330.5	6.25
PbSS4	Pbr022714.1	Chr3	1322262	1324033	+	183	20747.49	4.69
PbSS5	Pbr022715.1	Chr3	1325811	1328963	+	559	63581.31	7.25
PbSS6	Pbr008035.1	Chr5	3932753	3937176	+	818	91171.68	5.62
PbSS7	Pbr028302.1	Chr6	2843943	2847973	+	867	undefined	undefined
PbSS8	Pbr042506.1	Chr8	9179789	9184899	+	962	106746.1	6.06
PbSS9	Pbr033571.1	Chr9	252260	262865	+	1142	129417.5	8.64
PbSS10	Pbr008457.1	Chr9	484297	496090	-	1142	129417.5	8.64
PbSS11	Pbr008464.1	Chr9	651911	662128	+	1119	126822.5	8.66
PbSS12	Pbr031564.1	Chr11	13021557	13025667	+	815	91557.1	6.76
PbSS13	Pbr012029.1	Chr13	9093304	9095768	-	265	29773.02	5.91
PbSS14	Pbr014902.1	Chr13	12846483	12851084	-	800	91169.09	5.7
PbSS15	Pbr027868.1	Chr15	10137257	10142745	-	1054	117624	6.15
PbSS16	Pbr034361.1	Chr15	12427951	12433933	+	1066	119481.6	6.06
PbSS17	Pbr032090.1	Chr15	13956060	13960667	-	873	97214.54	5.94
PbSS18	Pbr037395.1	Chr15	17070500	17075187	-	837	96491.85	6.41
PbSS19	Pbr012642.1	Chr17	4602903	4606969	+	844	95523.44	6.81
PbSS20	Pbr012641.1	Chr17	4613998	4626884	+	1138	128711.8	8.53
PbSS21	Pbr003395.1	scaffold1142.0	15301	18735	+	807	92644.24	5.78
PbSS22	Pbr026341.1	scaffold424.0	208844	210615	+	183	20747.49	4.69
PbSS23	Pbr026338.1	scaffold424.0	167041	168812	-	183	20747.49	4.69

Table S1. *Cont.*

PbSS24	Pbr026337.1	scaffold424.0	165212	166512	-	63	7543.7	6.72
PbSS25	Pbr003394.1	scaffold1142.0	5534	9046	+	790	90749.18	5.97
PbSS26	Pbr026342.1	scaffold424.0	212581	215680	+	566	64281.07	7.62
PbSS27	Pbr026336.1	scaffold424.0	161976	165075	-	566	64281.07	7.62
PbSS28	Pbr009578.1	scaffold160.1.1	28772	34269	+	1054	117601	6.06
PbSS29	Pbr001616.1	scaffold1046.0	32129	36123	+	856	96122.99	6.48
PbSS30	Pbr001745.1	scaffold1051.0	77	3071	-	510	57267.35	6.78

Table S2. List of sucrose synthase gene sequences used in this study

**Taxon**	**Sequence name**	**Accession number**	**Protein sequence**	**Protein size**
A. Angiosperms				
Dicot plants				
Arabidopsis thaliana	AtSS1	At5g20830	MANAERMITRVHSQRERLNETLVSERNEVLALLSRVEAKGKGILQQN<br>QIIAEFEALPEQTRKKLEGGPFFDLLKSTQEAIVLPPWVALAVRPRPGV<br>WEYLRVNLHALVVEELQPAEFLHFKEELVDGVKNGNFTLELDFEPFN<br>ASIPRPTLHKYIGNGVDFLNRHLSAKLFHDKESLLPLLKFLRLHSHQGK<br>NLMLSEKIQNLNTLQHTLRKAEEYLAELKSETLYEEFEAKFEEIGLERG<br>WGDNAERVLDMIRLLLDLLEAPDPCTLETFLGRVPMVFNVVILSPHGY<br>FAQDNVLGYPDTGGQVVYILDQVRALEIEMLQRIKQQGLNIKPRILILT<br>RLLPDAVGTTCGERLERVYDSEYCDILRVPFRTEKGIVRKWISRFEVWP<br>YLETYTEDAAVELSKELNGKPDLIIGNYSDGNLVASLLAHKLGVTQCTI<br>AHALEKTKYPDSDIYWKKLDDKYHFSCQFTADIFAMNHTDFIITSTFQ<br>EIAGSKETVGQYESHTAFTLPGLYRVVHGIDVFDPKFNIVSPGADMSIY<br>FPYTEEKRRLTKFHSEIEELLYSDVENKEHLCVLKDKKKPILFTMARLD<br>RVKNLSGLVEWYGKNTRLRELANLVVVGGDRRKESKDNEEKAEMKK<br>MYDLIEEYKLNGQFRWISSQMDRVRNGELYRYICDTKGAFVQPALYE<br>AFGLTVVEAMTCGLPTFATCKGGPAEIIVHGKSGFHIDPYHGDQAAD<br>TLADFFTKCKEDPSHWDEISKGGLQRIEEKYTWQIYSQRLLTLTGVYGF<br>WKHVSNLDRLEARRYLEMFYALKYRPLAQAVPLAQDD	808
	AtSS2	At5g49190	MANAERVITRVHSQRERLDATLVAQKNEVFALLSRVEAKGKGILQHH<br>QIIAEFEAMPLETQKKLKGGAFFEFLRSAQEAIVLPPFVALAVRPRPGV<br>WEYVRVNLHDLVVEELQASEYLQFKEELVDGIKNGNFTLELDFEPFNA<br>AFPRPTLNKYIGDGVEFLNRHLSAKLFHDKESLHPLLKFLRLHSHEGK<br>TLMLNNRIQNLNTLQHNLRKAEEYLMELKPETLYSEFEHKFQEIGLER<br>GWGDTAERVLNMIRLLLDLLEAPDPCTLENFLGRIPMVFNVVILSPHG<br>YFAQDNVLGYPDTGGQVVYILDQVRALETEMLQRIKQQGLNITPRILII<br>TRLLPDAAGTTCGQRLEKVYGSQYCDILRVPFRTEKGIVRKWISRFEVW<br>PYLETFTEDVAAEISKELQGKPDLIIGNYSDGNLVASLLAHKLGVTQCT<br>IAHALEKTKYPDSDIYWKKLDEKYHFSCQFTADLIAMNHTDFIITSTFQ<br>EIAGSKDTVGQYESHRSFTLPGLYRVVHGIDVFDPKFNIVSPGADMSIY<br>FAYTEEKRRLTAFHLEIEELLYSDVENEEHLCVLKDKKKPIIFTMARLDR<br>VKNLSGLVEWYGKNTRLRELVNLVVVGGDRRKESQDNEEKAEMKK<br>MYELIEEYKLNGQFRWISSQMNRVRNGELYRYICDTKGAFVQPALYEA<br>FGLTVVEAMTCGLPTFATCNGGPAEIIVHGKSGFHIDPYHGDKAAESL<br>ADFFTKCKHDPSHWDQISLGGLERIQEKYTWQIYSQRLLTLTGVYGFW<br>KHVSNLDRLESRRYLEMFYALKYRPLAQAVPLAHEE	808

Table S2. *Cont.*

	AtSS3	At4g02280	MANPKLTRVLSTRDRVQDTLSAHRNELVALLSRYVDQGKGILQPHNL<br>IDELESVIGDDETKKSLSDGPFGEILKSAMEAIVVPPFVALAVRPRPGV<br>WEYVRVNVFELSVEQLTVSEYLRFKEELVDGPNSDPFCLELDFEPFNA<br>NVPRPSRSSSIGNGVQFLNRHLSSVMFRNKDCLEPLLDFLRVHKYKGH<br>PLMLNDRIQSISRLQIQLSKAEDHISKLSQETPFSEFEYALQGMGFEKG<br>WGDTAGRVLEMMHLLSDILQAPDPSSLEKFLGMVPMVFNVVILSPHG<br>YFGQANVLGLPDTGGQVVYILDQVRALETEMLLRIKRQGLDISPSILIV<br>TRLIPDAKGTTCNQRLERVSGTEHTHILRVPFRSEKGILRKWISRFDVW<br>PYLENYAQDAASEIVGELQGVPDFIIGNYSDGNLVASLMAHRMGVTQ<br>CTIAHALEKTKYPDSDIYWKDFDNKYHFSCQFTADLIAMNNADFIITS<br>TYQEIAGTKNTVGQYESHGAFTLPGLYRVVHGIDVFDPKFNIVSPGAD<br>MTIYFPYSEETRRLTALHGSIEEMLYSPDQTDEHVGTLSDRSKPILFSMA<br>RLDKVKNISGLVEMYSKNTKLRELVNLVVIAGNIDVNKSKDREEIVEIE<br>KMHNLMKNYKLDGQFRWITAQTNRARNGELYRYIADTRGAFAQPAF<br>YEAFGLTVVEAMTCGLPTFATCHGGPAEIIEHGLSGFHIDPYHPEQAG<br>NIMADFFERCKEDPNHWKKVSDAGLQRIYERYTWKIYSERLMTLAGV<br>YGFWKYVSKLERRETRRYLEMFYILKFRDLVKTVPSTADD	809
	AtSS4	At3g43190	MPTGRFETMREWVYDAISAQRNELLSLFSRYVAQGKGILQSHQLIDEF<br>LKTVKVDGTLEDLNKSPFMKVLQSAEEAIVLPPFVALAIRPRPGVREYV<br>RVNVYELSVDHLTVSEYLRFKEELVNGHANGDYLLELDFEPFNATLPR<br>PTRSSSIGNGVQFLNRHLSSIMFRNKESMEPLLEFLRTHKHDGRPMML<br>NDRIQNIPILQGALARAEEFLSKLPLATPYSEFEFELQGMGFERGWGDT<br>AQKVSEMVHLLLDILQAPDPSVLETFLGRIPMVFNVVILSPHGYFGQA<br>NVLGLPDTGGQVVYILDQVRALENEMLLRIQKQGLEVIPKILIVTRLLP<br>EAKGTTCNQRLERVSGTEHAHILRIPFRTEKGILRKWISRFDVWPYLET<br>FAEDASNEISAELQGVPNLIIGNYSDGNLVASLLASKLGVIQCNIAHAL<br>EKTKYPESDIYWRNHEDKYHFSSQFTADLIAMNNADFIITSTYQEIAGS<br>KNNVGQYESHTAFTMPGLYRVVHGIDVFDPKFNIVSPGADMTIYFPYS<br>DKERRLTALHESIEELLFSAEQNDEHVGLLSDQSKPIIFSMARLDRVKN<br>LTGLVECYAKNSKLRELANLVIVGGYIDENQSRDREEMAEIQKMHSLI<br>EQYDLHGEFRWIAAQMNRARNGELYRYIADTKGVFVQPAFYEAFGLT<br>VVESMTCALPTFATCHGGPAEIIENGVSGFHIDPYHPDQVAATLVSFFE<br>TCNTNPNHWVKISEGGLKRIYERYTWKKYSERLLTLAGVYAFWKHVS<br>KLERRETRRYLEMFYSLKFRDLANSIPLATDEN	807
	AtSS5	At5g37180	MEMTSGSLGNGIPEAMGQNRGNIKRCLEKYIENGRRVMKLNELMDE<br>MEIVINDVTQRRRVMEGDLGKILCFTQAVVIPPNVAFAVRGTPGNWQ<br>YVKVNSSNLSVEALSSTQYLKLKEFLFDENWANDENALEVDFGALDFT<br>LPWLSLSSSIGNGLSFVSSKLGGRLNDNPQSLVDYLLSLEHQGEKLMM<br>NETLNTARKLEMSLILADVFLSELPKDTPFQAFELRFKECGFEKGWGES<br>AGRVKETMRILSEILQAPDPQNIDRFFARVPRIFNVVIFSVHGYFGQTD<br>VLGLPDTGGQVVYILDQVKALEDELLQRINSQGLNFKPQILVVTRLIPD<br>AKKTKCNQELEPIFGTKYSNILRIPFVTENGILRRWVSRFDIYPYLERFTK<br>DATTKILDILEGKPDLIIGNYTDGNLVASLMANKLGITQATIAHALEKT<br>KYEDSDIKWKEFDPKYHFSSQFTADLISMNSADFIIASTYQEIAGSKERA<br>GQYESHMSFTVPGLYRVVSGINVFDPRFNIAAPGADDSIYFPFTAQDRR<br>FTKFYTSIDELLYSQSENDEHIGYLVDKKKPIIFSMARLDVVKNLTGLTE<br>WYAKNKRLRDLVNLVIVGGFFDASKSKDREEISEIKKMHSLIEKYQLK<br>GQFRWITAQTDRTRNGELYRSIADTRGAFVQPAHYEAFGLTVIEAMSC<br>GLVTFATNQGGPAEIIVDGVSGFHIDPSNGEESSDKIADFFEKSGMDPD<br>YWNMFSNEGLQRINECYTWKIYANKVINMGSTYSYWRHLNKDQKLA	836

Table S2. *Cont.*

			KQRYIHSFYNLQYRNLVKTIPILSDIPEPPPLPPKPLVKPSASKGSKRTQP RLSFRLFGA	
	AtSS6	At1g73370	MSSSSQAMLQKSDSIAEKMPDALKQSRYHMKRCFASFVGGGKKLMK REHLMNEIEKCIEDSRERSKILEGLFGYILTCTQEAAVVPPFVALAARPN PGFWEYVKVNSGDLTVDEITATDYLKLKESVFDESWSKDENALEIDFG AIDFTSPRLSLSSSIGKGADYISKFISSKLGGKSDKLEPLLNYLLRLNHHG ENLMINDDLNTVAKLQKSLMLAVIVVSTYSKHTPYETFAQRLKEMGFE KGWGDTAERVKETMIILSEVLEAPDNGKLDLLFSRLPTVFNVVIFSVHG YFGQQDVLGLPDTGGQVVYILDQVRALEEELLIRINQQGLGFKPQILV VTRLIPEARGTKCDQELEAIEGTKHSHILRVPFVTNKGVLRQWVSRFDI YPYLERFTQDATSKILQRLDCKPDLIIGNYTDGNLVASLMATKLGVTQ GTIAHALEKTKYEDSDAKWKELDPKYHFSCQFTADLIAMNVTDFIITST YQEIAGSKDRPGQYESHTAFTMPGLCRVVSGIDVFDPKFNIAAPGADQ SVYFPYTEKDKRFTKFHPSIQELLYNEKDNAEHMGYLADREKPIIFSMA RLDTVKNITGLVEWYGKDKRLREMANLVVVAGFFDMSKSNDREEKA EIKKMHDLIEKYKLKGKFRWIAAQTDRYRNSELYRCIADTKGVFVQPA LYEAFGLTVIEAMNCGLPTFATNQGGPAEIIVDGVSGFHIDPNNGDES VTKIGDFFSKCRSDGLYWDNISKGGLKRIYECYTWKIYAEKLLKMGSLY GFWRQVNEDQKKAKKRYIEMLYNLQFKQLTKKVTIPEDKPLPLRLASL RNLLPKKTTNLGAGSKQKEVTETEKTKQKSKDGQEQHDVKVGEREVR EGLLAADASERVKKVLESSEEKQKLEKMKIAYGQQHSQGASPVRNLF WSVVVCLYICYILKQRFFGANSAQEY	942
Pisum sativum	PsSS1	AJ012080	MATDRLTRVHSLRERLDETLTANRNEILALLSRIEAKGKGILQHHQVI AEFEEIPEENRQKLTDGAFGEVLRSTQEAIVLPPWVALAVRPRPGVWE YLRVNVHALVVENLQPAEFLKFKEELVDGSANGNFVLELDFEPFTASF PRPTLNKSIGNGVQFLNRHLSAKLFHDKESLHPLLEFLRLHSYKGKTL MLNDRIQNPDSLQHVLRKAEEYLGTVAPDTPYSEFEHRFQEIGLERGW GDTAERVLESIQLLLDLLEAPDPCTLETFLDRIPMVFNVVILSPHGYFAQ DDVLGYPDTGGQVVYILDQVRALESEMLNRIKKQGLDIVPRILIITRLLP DAVGTTCGQRLEKVYGTEHCHILRVPFRDQKGIVRKWISRFEVWPYLE TYTEDVAHELAKELQGKPDLIVGNYSDGNIVASLLAHKLGVTQCTIAH ALEKTKYPESDIYWKKFEEKYHFSCQFTADLFAMNHTDFIITSTFQEIA GSKDTVGQYESHTAFTLPGLYRVVHGIDVFDPKFNIVSPGADQTIYFPY TETSRRLTSFYPEIEELLYSTVENEEHICVLKDRSKPIIFTMARLDRVKNIT GLVEWYGKNAKLRELVNLVVVAGDRRKESKDLEEKAEMKKMYEHIE TYKLNGQFRWISSQMNRVRNGELYRVICDTKGAFVQPAVYEAFGLTV VEAMATGLPTFATLNGGPAEIIVHGKSGFHIDPYHGDRAADLLVEFFE KVKTDPSHWDKISQGGLQRIEEKYTWQIYSQRLLTLTGVYGFWKHVS NLDRLESRRYLEMFYALKYRKLAESVPLAVEE	806
	PsSS2	AJ001071	MSTHPKFTRVPSIRDRVQDTLSAHRNELISLLSRYVAQGKGILQPHNLI DELDNILGEDHATLDLKNGPFGQIINSAQEAIVLPPFVAIAVRPRPGV WEYVRVNVFELSVEQLSVSEYLSFKEELVEGKSNDNIILELDLEPFNASF PRPTRSSSIGNGVQFLNRHLSSNMFRNKDCLEPLLDFLRVHTYKGHAL MLNDRIQSISKLQSALVKAEDHLSKLAPDTLYSEFEYELQGTGFERGW GDTAARVLEMMHLLLDILQAPDPSTLETFLGRVPMVFNVVILSPHGFF GQANVLGLPDTGGQVVYILDQVRALESEMLVRIKKQGLDFTPRILIVTR LIPDAKGTTCNQRLERVSGTEYTHILRVPFRSEKGILRKWISRFDVWPFL ETFAEDVASEIAAELQCYPDFIIGNYSDGNLVASLLAYKMGVTQCTIA HALEKTKYPDSDIYWKKFEDKYHFSCQFTADLIAMNNADFIITSTYQEI AGTKNTIGQYESHTAFTLPGLYRVVHGIDVFDPKFNIVSPGADMTIYFP YSDKEKRLTALHSSIEKLLYGTEQTDEYIGSLTDRSKPIIFSMARLDRVK	809

Table S2. *Cont.*

			NITGLVESYAKNSKLRELVNLVVVAGYIDVKKSSDREEIEEIEKMHDLM<br>KQYNLNGEFRWITAQTNRARNGELYRYIADTKGAFVQPAFYEAFGLT<br>VVEAMTCGLPTFATNHGGPAEIIEHGVSGFHIDPYHPDQASELLVDFF<br>QRCKEDPNHWNKVSDGGLQRIYERYTWKIYSERLMTLAGVYSFWKYV<br>SKLERRETRRYLEMFYILKFRDLANSVPIAKG	
	PsSS3	AJ311496	MASLTHSTSLRQRFDETLTAHRNEILSLLSRIEAKGKGILQHHQIIAEFE<br>EIPEENRQKLVNGVFGEVLRSTQEAIVLVPFVALAVRPRPGVWEYLRV<br>DVHGLVVDELSAAEYLKFKEELVEGSSNENFVLELDFEPFNASIPKPTQ<br>NKSIGNGVEFLNRHLSAKLFHGKESLQPLLEFLRLHNHNGKTIMVND<br>RIQNLNSLQHVLRKAEDYLIKIAPETPYSEFEHKFQEIGLERGWGDTAE<br>RVVETIQLLLDLLDGPDPGTLETFLGRIPMVFNVVILSPHGYFAQDNVL<br>GYPDTGGQIVYILDQVRALEEEMLKRIKQQGLDITPRILIITRLLPDAVG<br>TTCGQRLEKVYNTEHCHILRVPFRTEKGIVRKWISRFEVWPYLETFSED<br>VANELAKELQGKPDLIVGNYSDGNIVASLLAHKLGVTQCTIAHALEK<br>TKYPESDIYWKKFDDKYHFSSQFTADLFAMNHTDFIITSTFQEIAGSKD<br>TVGQYESHTAFTLPGLYRVVHGIDVFDPKFNIVSPGADLSIYFPYTETER<br>RLTSFHPDIEELLYSTVENEEHICVLKDRSKPIIFTMARLDRVKNITGLVE<br>CYGKNARLRELVNLVVVAGDRRKESKDLEEIAEMKKMYGLIETYKLN<br>GQFRWISAQMDRIRNGELYRVICDTKGAFVQPAIYEAFGLTVIEAMSC<br>GLPTFATCNGGPAEIIVHGKSGYHIDPYHGDRAAETLVEFFEKSKADPT<br>YWDKISHGGLKRIHEKYTWQIYSDRLLTLTGVYGFWKHVTNLERRESK<br>RYLEMFYALKYSKLAESVPLAVEE	804
	PsSS4	AF079851	MATDRLTRVHSLRERLDETLTANRNEILALLSRIEAKGKGILQHHQVI<br>AEFEEIPEENRQKLTDGAFGEVLRSTQEAIVLPPWVALAVRPRPGVWE<br>YLRVNVHALVVENLQPAEFLKFKEELVDGSANGNFVLELDFEPFTASF<br>PRPTLNKSIGNGVQFLNRHLSAKLFHDKESLHPLLEFLRLHSYKGKTL<br>MLNDRIQNPDSLQHVLRKAEEYLGTVAPDTPYSEFEHRFQEIGLERGW<br>GDTAERVLESIQLLLDLLEAPDPCTLETFLDRIPMVFNVVILSPHGYFAQ<br>DDVLGYPDTGGQVVYILDQVRALESEMLNRIKKQGLDIVPRILIITRLLP<br>DAVGTTCGQRLEKVYGTEHCHILRVPFRDQKGIVRKWISRFEVWPYLE<br>TYTEDVAHELAKELQGKPDLIVGNYSDGNIVASLLAHKLGVTQCTIAH<br>ALEKTKYPESDIYWKKFEEKYHFSCQFTADLFAMNHTDFIITSTFQEIA<br>GSKDTVGQYESHTAFTLPGLYRVVHGIDVFDPKFNIVSPGADQTIYFPY<br>TETSRRLTSFYPEIEKLLYSTGGNEEHICVLKDRNKPIIFTMARLDRVKNI<br>TGLVEWYGKNAKLRELVNLVVVAGDRRKESKDLEEKAEMKKMYEHI<br>ETYKLNGQFRWISSQMNRVRNGELYRVICDTKGAFVQPAVYEAFGLT<br>VVEAMATGLPTFATLNGGPAEIIVHGKSGFHIDPYHGDRAADLLVEFF<br>EKVKTDPSHWDKISQGGLQRIEEKYTWQIYSQRLLTLTGVYGFWKHVS<br>NLDRLESRRYLEMFYALKYRKLAESVPLAVEE	806
Citrus unshiu	CuSS1	AB022092	MAERALTRVHSLRERLDETLSAHRNEILALLSRIEGKGKGILQNHQLIA<br>EFESISEENRKHLTEGAFGEVLRATQEAIVLAPWVALAVRPRPGVWEYI<br>RVNVHALVVEELLVAEYLHFKEELVDGGSNGNFVLELDFEPFNASFPR<br>PTLSKSIGNGVEFLNRHLSAKLFHDKESMHPLLEFLRVHCHKGKNMM<br>LNDRIQNLNSLQHVLRKAEEYLTTVVPETPFSELALRFQEIGLERGWG<br>DTAERALEMIQLLLDLLEAPDPCTLETFLGRIPMVFNVVILTPHGYFAQ<br>DDVVGYPDTGGQVVYILDQVRALEDEMLLRIKQQGLDITPQILIITRLL<br>PDAVGTTCGQRLEKVYGTKYSDILRVPFRTEKGVVRKWISRFEVWPYL<br>ETYTEDVAVEIAKELQGKPDLIIGNYSDGNIVASLLAHKLGVTQCTIAH<br>ALEKTKYPDSDIYWKNLDDKYHFSCQFTADLIAMNHTDFIITSTFQEIA<br>GSKDTVGQYESHTAFTLPGLYRVVHGIDVFDPKFNIVSPGADMSIYFPY<br>TEEKRRLKSFHPEIEELLYSDVENKEHLCVLKDRNKPILFTMARLDRVK	805

Table S2. *Cont.*

			NLTGLVEWYGKNAKLRELVNLVVVGGDRRKESKDLEEQAEMKKMYS LIDQNKLNGQFRWISSQMNRVRNGELYRYICETKGAFVQPALYEAFGL TVVEAMTCGLPTFATCKGGPAEIIVNGKSGYHIDPYHGEQAAEILVDF FEKCKADPSYWDKISLGGLKRIEEKYTWKIYSQRLLTLTGVYGFWKHV SNLDRLESRRYLEMFYALKYRKLAESVPLAVE	
	CuSS2	AB029401	MAERALTRVHSLRERLDETLSAHRNEILALLSRIEGKGKGILQNHQLIA EFESISEENRKHLTEGAFGEVLRATQEAIVLPPWVALAVRPRPGVWEYI RVNVHALVVEELLVAEYLHFKEELVDGGSNGNFVLELDFEPFNASFPR PTLSKSIGNGVEFLNRHLSAKLFHDKESMHPLLEFLRVHCHKGKNMM LNDRIQNLNSLQHVLRKAEEYLTTVAPETPFSELALRFQEIGLERGWG DTAERALEMIQLLLDLLEAPDPCTLETFLGRIPMVFNVVILTPHGYFAQ DDVLGYPDTGGQVIYILDQVRALEDEMLLRIKQQGLDITPQILIITRLLP DAVGTTCGQRLEKVYGTKYSDILRVPFRTEKGVVRKWISRFEVWPYLE TYTEDVAVEIAKELQGKPDLIIGNYSDGNIVASLLAHKLGVTQCTIAH ALEKTKYPDSDIYWKNLDDKYHFSCQFTADLIAMNHTDFIITSTFQEIA GSKDTVGQYESHTAFTLPGLYRVVHGIDVFDPKFNIVSPGADMSIYFPY TEEKRRLKSFHPEIEELLYSDVENKEHLCVLKDRNKPILFTMARLDRVK NLTGLVEWYGKNAKLRELVNLVVVGGDRRKESKDLEEQAEMKKMYS LIDQYKLNGQFRWISSQMNRVRNGELYRYICDTKGAFVQPALYEAFGL TVVEAMTCGLPTFATCKGGPAEIIVNGKSGYHIDPYHGEQAAEILVDF FEKCKADPSYWDKISLGGLKRIEEKYTWKIYSQRLLTLTGVYGFWKHV SNLDRLESRRYLEMFYALKYRKLAESVPLAVE	805
	CuSS3	AB022091	MAAPKLSRIPSIRERVEDTLSVHRNELVSLLSRYVAQGKGILQPHVLIDE LDNIFGDDEGRQNLRDGPFSEVIKSAQEAIVLPPFVAIAVRPRPGVWEY VRVNVYELSVEQLSVSEYLHFKEELVDAAFNERFVLELDFEPFNATFPR PNRSSSIGNGVQFLNRHLSSSMFRNKDCLEPLLDFLRAHKYKGHLLML NDRIQSISRLQSSLSKAEDHLSKLPPDTPFSQFEYVLQGMGFEKGWGDT AEHVLEMMHLLLDILQAPDPSTLEKFLGRLPMVFNVVILSPHGYFGQA NVLGLPDTGGQVVYILDQVRALENEMLLRIKRQGLDISPKILIVTRLIPD AKGTTCNQRLERVSGTEHTHILRVPFRSEKGILRQWISRFDVWPYLETF TEDVGSEITAELQGFPDFIIGNYSDGNLVASLLAYKMGITQCTIAHALE KTKYPDSDIYWKKFDEKYHFSCQFTADLIAMNNADFIITSTYQEIAGTK NTVGQYESHTAFTLPGLYRVVHGIDVFDPKFNIVSPGADMDIYFPYSEK QKRLTALHGSIEQLLFDPEQNDEHVGTLSDRSKPIVFSMARLDHVKN MTGLVECYGKNSRLRELVNLVVVAGYIDVNKSKDREEIAEIEKMHEL MKTYKLDGQFRWIAAQTNRARNGELYRYIADTKGAFVQPAFYEAFGL TVVEAMTCGLPTFATCHGGPAEIIEHGASGFHIDPYHPDQAAELMAD FFGKCKENPSHWKKISDGGLKRIYERYTWKIYSERLMTLAGVYGFWKY VSKLERRETRRYLEMFYILKFRDLVKSVPLASENQH	811
Citrus sinensis	CitSS1	Cs4g06850.1	MAERALTRVHSLRERLDETLSAHRNEILALLSRIEGKGKGILQNHQLIA EFESISEENRKHLTEGAFGEVLRATQEAIVLPPWVALAVRPRPGVWEYI RVNVHALVVEELLVAEYLHFKEELVDGGSNGNFVLELDFEPFNASFPR PTLSKSIGNGVEFLNRHLSAKLFHDKESMHPLLEFLRVHCHKGKNMM LNDRIQNLNSLQHVLRKAEEYLTTVVPETPFSELALRFQEIGLERGWG DTAERALEMIQLLLDLLEAPDPCTLETFLGRIPMVFNVVILTPHGYFAQ DDVLGYPDTGGQVVYILDQVRALEDEMLLRIKQQGLDITPQILIITRLLP DAVGTTCGQRLEKVYGTKYSDILRVPFRTEKGVVRKWISRFEVWPYLE TYTEDVAVEIAKELQGKPDLIIGNYSDGNIVASLLAHKLGVTQCTIAH ALEKTKYPDSDIYWKNLDDKYHFSCQFTADLIAMNHTDFIITSTFQEIA GSKDTVGQYESHTAFTLPGLYRVVHGIDVFDPKFNIVSPGADMSIYFPY TEEKRRLKSFHPEIEELLYSDVENKEHLCVLKDRNKPILFTMARLDRVK	805

Table S2. *Cont.*

			NLTGLVEWYGKNAKLRELVNLVVVGGDRRKESKDLEEQAEMKKMYS LIDQYKLNGQFRWISSQMNRVRNGELYRYICDTKGAFVQPALYEAFGL TVVEAMTCGLPTFATCKGGPAEIIVNGKSGYHIDPYHGEQAAEILVDF FEKCKADPSYWDKISLGGLKRIEEKYTWKIYSQRLLTLTGVYGFWKHV SNLDRLESRRYLEMFYALKYRKLAESVPLAVE	
	CitSS2	Cs4g06900.1	MFRFPPYFVFVCYSIERLGCGTYKRQQILSLLEAESNGAAIADVLNATQ EAAVSSPWVAFAVRTSPGVWCYIRVNVQTVDVEEISVSKYLLFKEEIVD GRKSNGNFAFEVDFEPFRALPHPTLSNSIGHGMEFLNRHMSAKLFND KESMQSLLEFLRVHSHMGKNMMLNEKIQDLGTLQSSLRMAEKYLSML APDTSYAEFEQKFQEIGLERGWGDNAEHVLGMIQLLLDLLQAPESSTL ETFLGKIPRVFNVVIFTPHGYFAQDNVLGYPDTGGQVVYILDQVRALE NEMLLRIKQQGLDITPRILIITRLLPDAVGTTCGQRVEKVYGTKYSDILR VPFRTEEGIVRKWISRFEVWPYLETFTEDVATEIIQELQCKPDLIIGNYSD GNIVASLLAHKLDVTQCTIAHALELTKYPDSDINWKKLDDKYHFSCQF TADLFAMNRTDFIITSTFQEIAGSKDTVGQYESHTAFSLPGLYRVVNGI DAFDPKFNIVSPGADMTIYFPYMEEKRRLKHFHSEIEELLYSPVENKEH LCVLKDSSKPILFTMARLDRVKNLTGLVEWYGKNAKLRELVNLVVVG GDRRKESKDLEEQAEMKKMYGLVDTYKLNGQFRWISSQMNRVRNGE LYRYICDTKGAFVQPALYEAFGLTVVEAMTCGLPTFATCNGGPAEIIV HGKSGFHIDPYKGDQAAGILVDFFEKCKVDPGHWDEISQGGLKRIQE KYTWKIYSERLLNLSGVYGFWKHLSKLDSREKNRYLEMFYSLMYRKQ VQTVPLAVDE	780
	CitSS3	Cs5g33470.1	MAAPKLSRIPSIRERVEDTLSVHRNELVSLLSRYVAQGKGILQPHVLIDE LDNIFGDDEGRQNLRDGPFSEVIKSAQEAIVLPPFVAIAVRPRPGVWEY VRVNVYELSVEQLSVSEYLHFKEELVDAAFNERFVLELDFEPFNATFPR PNRSSSIGNGVQFLNRHLSSSMFRNKDCLEPLLDFLRAHKYKGHLLML NDRIQSISRLQSSLSKAEDHLSKLPPDTPFSQFEYVLQGMGFEKGWGDT AEHVLEMMHLLLDILQAPDPSTLEKFLGRLPMVFNVVILSPHGYFGQA NVLGLPDTGGQVVYILDQVRALENEMLLRIKRQGLDISPKILIVTRLIPD AKGTTCNQRLERVSGTEHTHILRVPFRSEKGILRQWISRFDVWPYLETF TEDVGSEITAELQGFPDFIIGNYSDGNLVASLLAYKMGITQCTIAHALE KTKYPDSDIYWKKFDEKYHFSCQFTADLIAMNNADFIITSTYQEIAGTK NTVGQYESHTAFTLPGLYRVVHGIDVFDPKFNIVSPGADMDIYFPYSEK QKRLTALHGSIEQLLFDPEQNDEHVGTLSDRSKPIVFSMARLDHVKN MTGLVECYGKNSQLRELVNLVVVAGYIDVNKSKDREEIAEIEKMHEL MKTYKLDGQFRWIAAQTNRARNGELYRYIADTKGAFVQPAFYEAFGL TVVEAMTCGLPTFATCHGGPAEIIEHGASGFHIDPYHPDQAAELMAD FFGKCKENPSHWKKISDGGLKRIYERYTWKIYSERLMTLAGVYGFWKY VSKLERRETRRYLEMFYILKFRDLVKSVPLASENQH	811
	CitSS4	Cs5g16700.1	MSSSPSLKRSDTIADTMPDALRQSRYYMKKCFSRFVAKGKRLMKRHH LMDEVEKSIEDKIERGKVLEGLLGYILSSTQEAAVVPPNVAFAVRPNPG SWEYVKVNSEDLTVDGINVLEYLKFKETIFDQDWAKDENALELDFGA MDFSSPRLTLSSSIGNGVNYVSKFMSTRLSANSEKAKQFLDYLLALNHR GEQLMINDTLDTVDKLQAALIVAEVSISDLPKDTPYQEFQQRFKEWGF EKGWGNTAERVRETMRLFSEVLQAPDAAKLQVLFSRLPNMFNVVIFS PHGYFGQADVLGLPDTGGQVVYILDQVRALEEELLLRIKQQGLSVKPQ ILVVTRLIPNSKGTKCNQELEPIYDTKHSHILRIPFKTEQAILPQWVSRFD IYPYLGRFAQDATAKILDLMEGKPDLIIGNYSDGNLVASLMASKLGITQ ATIAHALEKSKYEDSDAKWKELDPKYHFSCQFTADLIAMNQTDFIITST YQEIAGSKDRPGQYESHTAFTMPGLCRVVSGINVFDPKFNIAAPGADQ SVYFPYTEKQKRLTSFHPDIEELLYSKEDNSEHIGYLADRKKPIIFSMARL	867

Table S2. *Cont.*

			DTVKNITGLTEWYGKNKRLRNMVNLVVVAGFFDPSKSHDREEIAEIK<br>KMHTLIEKYQLQGQFRWIAAQTDRYRNGELYRCIADTKGAFVQPALY<br>EAFGLTVIEAMNCGLPTFATNQGGPAEIIIDGVSGFHIDPNNGDESSNK<br>IADFFEKCKTDAGYWNQMSAAGRQRIYECYTWKIYANKVLNMGSIY<br>GFWRQINKEPKEAKQRYIQMFYSLLFRKLASNVPIKVPEPLQSAQTSPV<br>ESQQPAAATGIAKPQPPASAVIDKPNQQEKTAQQKKRHVRKTMTVI	
	CitSS5	Cs6g15930.1	MASATSLKRSDSIADNMPDALKQSRYHMKRCFVRYIEKGKRIMKLHD<br>LMDELNEVIDDEDVRTQVLEGLLGYILCSTQEAVVMPPHVAFAIRPNP<br>GFWEFVKVNSDDLSVEAITVTDFLKFKELVFDEDWAKDENALEVDFG<br>AYEFSLPQLTLSSSIGNGISFVSKFVTAKLSGRQDCAQPLVDYLLSLDHQ<br>GEKLMINDNLNTAEKLQMALIVAEVSLSTLPKDTPYQKFELRFKEWGF<br>EKGWGHTAERVRETMRSLSEVLQAPDPLHMEKFLSSLPILFNVVIFSPH<br>GYFGQADVLGLPDTGGQVVYILDQVKALEEELLLRIKQQGLYIKPQIV<br>VVTRLIPDARGTKCNQELEPIEGTKHSNILRVPFKTDKGILHRWVSRFD<br>VYPYLEGFAQDATTMILELLGGKPDLIIGNYSDGNLVASLMASKLGITQ<br>ATIAHALEKTKYEDSDVKWKELDPKYHFSCQFIADTIAMNATDFIIAST<br>FQEIAGSKDRPGQYESHTAFTLPGLCRVVKGIDVLDPKFNIAAPGADQ<br>SVYFPYTEKQRRLTKFHPEIEELLYNKEDNNEHIGYLADRKKPIIFSMAR<br>LDVVKNLTGLTEWYGKNKRLRNLVNLVIVGAFFDPSKSKDREETAEIK<br>KMHALMEKYQLKGQMRWIAAQSDRLRNGELYRCIADTKGAFVQPA<br>LYEAFGLTVIEAMNCGLPTFATNQGGPAEIIVDGVSGFHIDPYNGDESS<br>DKIADFFEACKVDPTYWNKFSTEGLKRINECYTWKIYANKMLNMGC<br>MYSFWKQLNKGQKLAKQRYIEMFYNLLFKNLVKNVPVPNEEAQQPM<br>SEPAVKPQHSLRQARSSTNMLHF	839
	CitSS6	Cs9g03980.1	MPHSKLDSMRDRVQDTLSVHRNELVSLLSRYAGKGKGILQRHHLTEE<br>MDDIVKEDEGMQKLSKSPFMKVLQSAQEAIILPPFVVLAVRPRPGVWE<br>YVRVNVYELSVDRLNVAEYLKSKEELVEGQSGDNYVLELDLEPFNATF<br>PRPTRSSSIGNGVQFLNRHLSSVMFRNKESLEPLLNFLRVHKHDGFVM<br>MLNDRIQSISKLQSALQRAEEYLSKFPPDTPYSEFEFEIQGMGFERGWG<br>DTAQRVSEMVHLLLDILQAPDPATLETFLGRIPMVFNVVIVSPHGYFG<br>QANVLGLPDTGGQVVYILDQVRALENEMLLRIQNQGLDVIPKILIVTR<br>LIPDAKGTTCNQRLERISGTEHTHILRVPFRTENGILRKWISRFDVWPYL<br>ETFAEDASNEIAAELQGVPDLIIGNYSDGNLVATLLSYKLGVTQCNIA<br>HALEKTKYPDSDLYWRKFEEKYHFSSQFTADLTAMNNADFIITSTYQEI<br>AGSKNNVGQYENHTAFTLPGLYRVVHGIDVFDPKFNIVSPGADMCIY<br>FPYSDKEKRLIALHGQIEDLLYDPKQNDEHVGILNDRSKPLIFSMARLD<br>GVKNLTGLVECYGKSSKLRELVNLVVVGGYMDVKNSRDREEMAEIEK<br>MHGLIKQYNLHGQFRWISAQMNRVRNGELYRYIADTRGVFVQPAFY<br>EAFGLTVVEAMTCGLPTFATCHGGPAEIIEHGVSGFHIDPYHPDQVAE<br>LMIEFFEKCYNDPSHWNKISDGGLKRIYERYTWKIYSERLLTLAGVYGF<br>WKYVSKLDRRETRRYLEMFYILKFRDLAKSVRLAVDEQH	808
Populus tomentosa	PtSS1	GU559727	MAERALTRVHSIRERVDETLKAHRNEIVALLTRIESKGKGILQHHQIVA<br>EFEAIPEDTRKTLAGGAFAEVLRSTQEAIVVPPWIALALRPRPGVWEYI<br>RLNVQALVVEELRVAEYLHFKEELVDGGCNGKFVLELDFEPFNASFPR<br>PTLSKYIGNGVEFLNRHLSAKLFHDKESLHPLLAFLKVHCHKGKNMM<br>LNDRIRNLDSLQYVLRKAEEFLSALKPDTPYSQFEHKFQEIGLERGWG<br>DTAERVLEMIRLLLDLLEAPDPCTLETFLGRIPMVFNVVIMSPHGYFAQ<br>DNVLGYPDTGGQVVYILDQVRALENEMLQRIKKQGLDITPRILIITRLL<br>PDAAGTTCGQRLEKVYGSEHCDILRVPFRDGKGMVRKWISRFEVWPY<br>LETFTEDVAAEIAKELQGKPDLIIGNYSDGNIVASLLAHKLGVTECTIA<br>HALEKTKYPDSDIYWKKFDEKYHFSCQFTADLFAMNHTDFIITSTFQEI	805

Table S2. *Cont.*

			AGSKDTVGQYESHTAFTLPGLYRVVHGIDVFDPKFNIVSPGADESIYFP YTDEKRRLTSFHPEIDELLYSPVENEEHLCVLKDRNKPILFTMARLDRV KNLSGLVEWYGKNTKLRELVNLVVVGGDRRKESKDLEEQAEMKKMY SHIEKYNLNGQFRWISSQMNRVRNGELYRYICDTKGAFVQPALYEAFG LTVVEAMTCGLPTFATCNGGPAEIIVHGKSGFHIDPYHGEQAAELLVD FFEKCKVDPAHWDKISHGGLQRIQEKYTWQIYSQRLLTLTGVYGFWK HVSNLDRLESRRYMEMFYALKYRKLAESVPLTKE	
	PtSS2	GU559728	MAALTRVQSIRERLDETLKTHRNEIVALLTRIEGKGKGILQHHQIIAEFE AIPEEIRKILAGGAFSEVLRSTQEAIVLPPWVALAVRPRPGVWEYVRVN VQALVVEELRVAEYLHFKEELVDGGSNGNFVLELDFEPFSASFPRPTLS KYIGNGVEFLNRHLSAKLFHDKESLHPLLAFLKVHCHKGKNMMLND RIQNLDSLQYVLRKAEEYLSSLKPETPYSQFEHKFQEIGLERGWGNTAE RVLQMIQLLLDLLEAPDPCTLETFLGRIPMVFNVVIMSPHGYFAQDNV LGYPDTGGQVVYILDQVRALENEMLLRIKQQGLDIIPRILIITRLLPDAV GTTCGQRLEKVYGSEHCDILRVPFRDEKGMVRKWISRFEVWPYLETYT EDVAAEIAKELQGKPDLIIGNYSDGNVVASLLAHKLGVTECTIAHALE KTKYPDSDIYWKKFDEKYHFSCQFTADLFAMNHTDFIITSTFQEIAGSK DTVGQYESHTAFTLPGLYRVVHGIDVFDPKFNIVSPGADESIYFPYTEE KLRLTSFHEEIEELLYSPVENDEHLCVLKDRSKPILFTMARLDRVKNLT GLVEWYGKNTKLRELANLVVVGGDRRKESKDIEEQAEMKKMYNHIE KYNLNGQFRWISSQMNRVRNGELYRYICDTKGAFVQPALYEAFGLTV VEAMTCGLPTFATCNGGPAEIIVHGKSGFHIDPYHGVQAAELLVDFFE KCKADPTYWDKISQGGLQRIQEKYTWKIYSQRLLTLTGVYGFWKHVS NLDHRESRRYLEMFYALKYRKLADSVPLTIE	803
Populus trichocarapa	PtrSS1	GU559729	MAERALTRVHSIRERVDETLKAHRNEIVALLTRIEGKGKGILQHHQIV AEFEAIPEDTRKTLAGGAFAEVLRSTQEAIVVPPWIALALRPRPGVWEY IRLNVQALVVEELRVAEYLHFKEELVDGGCNGNFVLELDFEPFNASFP RPTLSKYIGNGVEFLNRHLSAKLFHDKESLHPLLAFLKVHCHKGKNM MLNDRIRNLDSLQYVLRKAEEFLSTLKPDTPYSQFEHKFQEIGLERGW GDTAERVLEMIRLLLDLLEAPDPCTLETFLGRIPMVFNVVIMSPHGYFA QDNVLGYPDTGGQVVYILDQVRALENEMLQRIKKQGLDIIPRILIITRLL PDAVGTTCGQRLERVYGSEHCDILRVPFRDGKGMVRKWISRFEVWPY LETFTEDVAAEIAKELQGKPDLIIGNYSDGNIVASLLAHKLGVTECTIA HALEKTKYPDSDIYWKKFDEKYHFSCQFTADLFAMNHTDFIITSTFQEI AGSKDTVGQYESHTAFTLPGLYRVVHGIDVFDPKFNIVSPGADESIYFP YTDEKRRLTSFHPEIDELLYSPVENEEHLCVLKDRNKPILFTMARLDRV KNLSGLVEWYGKNTKLRELVNLVVVGGDRRKESKDLEEQAEMKKMY SHIEKYNLNGQFRWISSQMNRVRNGELYRYICDTKGAFVQPALYEAFG LTVVEAMTCGLPTFATCNGGPAEIIVNGKSGFHIDPYHGEKAAELLVD FFEKCKVDPAHWDKISHGGLQRIQEKYTWQIYSQRLLTLTGVYGFWK HVSNLDRLESRRYMEMFYALKYRKLAESVPLTKE	805
	PtrSS2	GU559730	MSVLTRVQSIRERLDETLKTHRNEIVALLTRIEGKGKGILQHHQIIAEFE AIPEEIRKILAGGAFSEVLRSTQEAIVLPPWVALAVRPRPGVWEYVRVN VQALVVEELRVAEYLHFKEELVDGGSNGNFVLELDFEPFSASFPRPTLS KYIGNGVEFLNRHLSAKLFHDKESLHPLLAFLKVHCHKGKNMMLND RIHNLDSLQYVLRKAEEYLSSLKPETPYSQFEHKFQEIGLERGWGNTAE RVLQMIQLLLDLLEAPDPCTLETFLGRIPMVFNVVIMSPHGYFAQDNV LGYPDTGGQVVYILDQVRALESEMLLRIKQQGLDITPRILIITRLLPDAV GTTCGQRLEKVYGSEHCDILRVPFRDEKGMVRKWISRFEVWPYLETYT EDVAAEIAKELQGKPDLIIGNYSDGNVVASLLAHKLGVTECTIAHALE KTKYPDSDIYWKKFDEKYHFSCQFTADLFAMNHTDFIITSTFQEIAGSK	803

Table S2. *Cont.*

			DTVGQYESHTAFTLPGLYRVVHGIDVFDPKFNIVSPGADESIYFPYTEK KLRLTSFHEEIEELLYSSVENDEHLCVLKDRNKPILFTMARLDRVKNLT GLVEWYGKNTKLRELANLVVVGGDRRKESKDIEEQAEMKKMYSHIEK YKLNGQFRWISSQMNRVRNGELYRYICDTKGAFVQPALYEAFGLTVV EAMTCGLPTFATCNGGPAEIIVHGKSGFHIDPYHGVQAAELLVDFFEK CKADPSYWDKISQGGLQRIQEKYTWKIYSQRLLTLTGVYGFWKHVSN LDHRESRRYLEMFYALKYRKLADSVPLTIE	
	PtrSS3	GU559731	MANPKLERIPSMRERVQDTLSANRNVLVSLLSRYVEQGKGILHPNNLI DELDNIVCDDAARLSLKDGPFSEVLKAAQEAIVLPPFVAVSIRPRPGV WEYVRVDVSQLNVEELTVSQYLRFKEELVDGPSNDPYVLELDFEPFNA AFPRPTRSSSIGNGVQYLNRHLSSNMFRNKDTLEPLLDFLRVHKYKGH ALMLNDRIKSVSRLQSALLKAEEYISKLPSETLYTEFEYTFQGMGFERG WGDTAARVLEMMHLLLDILQAPDPSTLETFLGRVPMVFNVVILSPHG YFGQANVLGLPDTGGQIVYILDQVRALENEMLLRIQQQGLDFKPKILI VTRLIPDSKGTSCNQRLERVSGTEHTHILRVPFRSEHGILRKWISRFDVW PYLETFAEDAASEIVAELQGIPDFIIGNYSDGNLVASLLAYKMGVTQCT IAHALEKTKYPDSDIYWKKFDDKYHFSCQFTADVLAMNNADFIITSTY QEIAGTKTTVGQYESHTAFTLPGLYRVVHGINVFDTKFNIVSPGADMD IYFPYSDKQKRLTTLHGSIEKMLYDSEQTDDWIGTLTDKSKPIIFSMARL DRVKNISGLVECYGKNARLRELVNLVVVAGYIDVKKSNDREEILEIEK MHELMKKYKLDGQFRWLTAQTNRARNGELYRYIADTKGAFVQPAFY EAFGLTVVEAMTCGLPTFATCHGGPAEIIEHGVSGFHMDPYYPDQAA EFMADFFEKCKDDPSYWKKISDAGLQRIYERYTWKIYSERLMTLAGVY GFWKYVSKLERRETRRYLEMFYILKFRDLVKTVPLSIEDWH	811
	PtrSS4	GU559732	MATLKRSDSIADNMPEALKQSRYHMKKCFAKYIEKGRRTMKLQQLLD EMENVIDDQVERTRVLEGLLGDIWFSIQEAVVNPPYVAFSIRPSPGFWE YVKVNSANLSVEGITVTDYLKFKEMIYDENWAKDANALEVDFGAFDF SVPHLTLSSSIGNGLGFVSKFVTSKLSGRLENAQPLVDYLLSLNRQGEK LMINETLGTVGKLQMALIVAEVYLSGLAKDTPYQNFEISFKEWGFEKG WGDTAERVKETMRCLSEVLQAPDPMNMEKFLSRLPTVFNVVIFSPHG YFGQADVLGLPDTGGQVVYILDQVKALEEELLLRIKQQGLNVKPQIVV ATRLIPDARGTTCNLEFEAIDGTKYSNILRVPFRVENRVLRQWVSRFDE VTTKILDLMEGKPDLIIGNYTDGNFAATLMAGKLGITQATIAHALEKT KYENSDVKWKELESKYHFPCQFMADIVAMNATDFIIASTYQEIAGSKD RTGQYESHAAFTLPGLCRVVSGVNVFDPKFNIAAPGADQSVYFPHTEK QSRFTQFNPDIEELLYSKVVNDEHIGYLEDKKKPIIFSMARLDTVKNLT GLTEWYGKNKRLRGLVNLVIVGGFFDPNKSKDREEMAEIKKMHELIE KYQLKGQIRWIAAQTDRKRNGELYRCIADTKGAFVQPALYEAFGLTVI EAMNCGLPTFATNQGGPSEIIVDGISGFHIDPKNGDESSNIIADFFEKCK VDPGHWNKYSLEGLKRINECYTWKIYANKLLNMGNVYSFWRQLNKE QKLAKQRYIQLFFNLKFRELVQSVPIPTEEAQTPASEPTARTQSSAR	815
	PtrSS5	GU559733	MATLKRSDSIADNMPEALKQSRYHMKRCFAKYIEKGRRTMKLQQLLD EMENVIDDQVERTRVLQGLLGDIWFSIQEAVVNPPYVALSIRPSPGFWE FVKVNSADLSVEGITATDYLKFKEMIYDENWAKDANALEVDFGAFDF SVPHLTLSSSIGNGLGFVSKFATSKLSGRLESAQPLVDYLLSLNHEGEKL MINETLSSVRKLRMALIVAEAYLSGLPKDTQYQNFETSFKAWGFEKGW GNTAERVKETMRCLSEVLQAPDPLNMENFFSRLPTVFNVVIFSPHGYF GQADVLGLPDTGGQVVYILDQVKALEDELLLRIEQQGLNIKPQIVVVT RLIPEARGTKCNQELESINGTKHSNILRVPFSIENKVLRQWVSRFDDVIT KLLDLMQRKPDLIIGNYTDGNLAATLMASKLGITQATIAHALEKTKYE NSDVKWKELDPKYHFSCQFMADTIAMNATDFIIASTYQEIAGSKDRPG	835

Table S2. *Cont.*

			QYESHASFTLPGLCRVVSGIDVFDPKFNIAAPGADQSVYFPYTEKQSRF TKFHPAIEELLYSKVVNDEHIGYLEDKKKPIIFSMARLDTVKNLTGLTE WYGKNKRLRGLVNLVIVGGFFDPNKSKDREEMAEITKMHGLIKKYRL NGQFRWIAAQTDRNRNGELYRCIADTKGAFVQPALYEAFGLTVIEAM NCGLPTFATNQGGPAEIIVDGISGFHIDPQNGDESSNIIADFFEKCKVD PGYWNKFAAEGLKRINECYTWKIYAKKLLNMGNMYSFWRQLNKEQ KLAKQRYIQMLYNLQFRRLVVWSLSCNQEAWIRTAIILCHLTLLNMFI HNVFYLLLSEFNFSVYDH	
	PtrSS6	GU559734	MASQTALQRSETITESMPEALRQSRYHMKKCFSRFVAPGKRLMKRQH LMDEVDESIQDKNERQKVLEGLLGYILSCTQEAAVIPPFVAFAVRPNP GFWEYVKVNAEDLSVEGISVSEYLQLKEMVFDEKWANNENALELDFG AMDFSTPRLTLSSSIGNGVNYMSKFMSSKLSGSSEAAKPLLDYLLALNH QGENLMINQTLDTVAKLQEALIVAEVVVSAFPKDTPYQDFQQRLREL GFETGWGDTAERVKETMRLLSESLQAPYPMKLQLLFSRIPNMFNIVIFS PHGYFGQSDVLGLPDTGGQVVYILDQVRALEEELLLKIKHQGLGVKPR ILVVTRLIPNAGGTKCNQEVEPIFGTQHSHIVRVPFKTEKGVLPQWVSR FDDAADKVLEHMDSKPDLIIGNYSDGNLVASLMARKLSITLGTIAHAL EKTKYEDSDVKWKELDAKYHFSCQFTADMIAMNSADFIITSTYQEIAG SNVRPGQYESHTAFTMPGLCRVVSGINVFDPKFNIASPGADQSVYFPY TEKQKRLTSFHPAIEELLYSNEDNHEHIGYLADRKKPIIFSMARLDTVK NITGLTEWFGKNTKLRNLVNLVVVAGFFDPSKSNDREEIAEIKKMHAL IEKYQLKGQFRWIAAQTDRYRNGELYRCIADTKGAFVQPALYEAFGLT VIEAMNCGLPTFATNQGGPAEILVDGISGFHIDPNNGDESSNKIADFFE KCKTDAEYWNKMSAAGLQRIYECYTWKIYANKVLNMGSVYGFWRQ TNKEQKLAKQRYIEAFYNLQFNNLVGYCGQLVL	800
	PtrSS7	GU559735	MASAPVLKRSETIAESMPDALRQSRYHMRICFSRFVAPGRRLMKRQHI MDEVDKSIQDKNERQKVLEGLLGYILSSTQEAAVVPPFVAFAVRPNPG FWEYVKVNAEDLSVDGISVSEYLQFKEMIFDEKWASNENALEVDFGA MDFSTPRLTLSSSIGNGLNYMSKFMSSKLRGNSDAAKPLLDYLLALDH QGENLMINQALDSVSKLQAALIVAEVVVSAFPKDAPYQDFQQSLKRL GFEKGWGDTAERVKETMRMLSESLQAPEPVKLELLFSRIPNVFNIVIFS PHGYFGQSDVLGLPDTGGQIVYILDQVRALEEELLLKIRQQGLSVKPQI LVITRLIPHAGGTKCNQEVEPIFGTKHSHIVRVPFKTEKGVLPQWVSRF DVYPYLERFAQDAADKVREHMDCKPDLLIGNYSDGNLVASLMAQKL GTTLGTIAHALEKTKYEDSDAKWKELDPKYHFSCQFTADMIAMNTAD FIITSTYQEIAGSKNRPGQYESHVAFTMPGLCRVVSGINVFDPKFNIASP GADQTVYFPYTEKQKRLTSFHPAIEELLYNNEDNNEHIGYLADKKKPII FSMARLDTVKNITGLTEWYGKNAKLRNLVNLVVVAGFFDPSKSNDRE EIAEIKKMHSLIEKYQLKGQFRWIAAQSDRYRNGELYRCIADTKGAFIQ PALYEAFGLTVIEAMNCGLPTFATNQGGPAEIIVDGISGFHIDPNNGDE SSNKIADFVEKCKTDAEYWNKMSATGLQRIYECYTWKIYANKVLNM GSVYGFWRQMNKEQKLLKQRYIEAFYNLQFRNLVGYFRQLVT	810
Glycine max	GmSS1	Glyma.02G2 40400	MASAPALKRTDSVVDNMPDALRQSRYHMKRCFAKYLEKGRRIMKLH HLMEEMELVIDDKSERSQVLEGILGFILSSTQEAVVDPPYVAFAIRPNPG VWEFVKVSSEDLSVEAITPTDYLKFKERVHDEKWATDENSFEADFGAF DSQIPLLTLSSSIGNGLEFTSKFLTSKLTGKLEKTQAIVDYLLTLNHQGES LMINDSLNSAAKLQMALVVADAFLSGLSKDTAYQNFELRFKEWGFER GWGDTAGRVKETMRTLSEVLQAPDPMNLEKFLSNLPIIFNVVIFSVHG YFGQADVLGLPDTGGQVVYILDQVKSLEAELLLRIRQQGLNVKPQILV VTRLIPDARGTKCHHELEPISDTKHSHILRVPFQTDKGILRQWISRFDIY PYLERFTQDATAKILEFMEGKPDLVIGNYTDGNLVASLMARKLGITQG	840

Table S2. *Cont.*

			TIAHALEKTKYEDSDVKWKELDPKYHFSCQFMADTVAMNASDFIITST YQEIAGSKDRPGQYESHAAFTLPGLCRVVSGINVFDPKFNIVAPGADQ SVYFPYTEKEKRLSQFHPAIEDLLFSKVDNIEHIGYLADRRKPIIFSMARL DVVKNLSGLVEWYGKNKRLRNLVNLVIVGGFFDPSKSKDREEMAEIK KMHDLIDKYQLKGQFRWIAAQTNRYRNGELYRCIADTRGAFVQPALY EAFGLTVIEAMNCGLPTFATNQGGPAEIIVDGVSGFHIDPLNGEESSNK IADFFEKCKVNQSQWNVISEAGLQRINECYTWKIYANKMVNMGNIYT FWRQVNKEQKEAKQRYIQMFYNLIFKNLVKTVPAPSDEPQQPVGKQP SLKSRSTGRSQSRLQRLFGN	
	GmSS2	Glyma.03G216300	MSTQPKLGRISSIRDRVEDTLSAHRNELISLLSRYVAQGKGILQPHNLID ELDNIPGDDEAIVDLKNGPFGEIVKSAKEAIVLPPFVAIAVRPRPGVWE YVRVNVSDLSVEQLSISEYLSFKEELVDGKINENFVLELDFEPFNATFPR PTRSASIGNGVQFLNRHLSSIMFRNKDSLQPLLDFLRAHKYKGHALMI NDRVQTISNLQSALAKTEDYLSKLASDTLYSEFEYVLQGMGFERGWGD TAERVLEMMHLLLDILQAPDPSTLETFLGRVPMVFNVVILSPHGYFGQ ANVLGLPDTGGQVVYILDQVRALENEMLLRIKKQGLDFTPRILIVTRLI PDAKGTTCNQRLERVSGTDHTHILRVPFRSESGTLRKWISRFDVWPYLE TYAEDVASEIAAELQGYPDFIIGNYSDGNLVASLLAYKMGVTQCTIAH ALEKTKYPDSDLYWKKFEDKYHFSCQFTADLIAMNNADFIITSTYQEIA GTKNTVGQYESHTGFTLPGLYRVVHGIDVFDPKFNIVSPGADMSIYFPY SEKQNRLTALHGSIEKLLFDPEQTDEYIGSLKDKSKPIIFSMARLDRVKN ITGLVECFGKNSKLRELVNLVVVAGYIDVKKSSDREEIAEIEKMHELMK KYNLNGDFRWIAAQTNRARNGELYRYIADTQGAFIQPAFYEAFGLTV VEAMTCGLPTFATCHGGPAEIIEHGISGFHIDPYHPDQASELLVEFFQK SKEDPDHWKKISNGGLQRIYERYTWKIYSERLMTLAGVYSFWKYVSKL ERRETRRYLEMFYILKFRDLANSVPLAKDDAS	812
	GmSS3	Glyma.09G073600	MANHPLTHSHSFRERFDETLTGHRNEILALLSRLEAKGKGILQHHQVV AEFEEIPEESRKKLQDGVFGEVLRSTQEAIVLPPFVALAVRPRPGVWEY LRVNVHMLVVDELRPAEYLRFKEELVEGSSNGNFVLELDFEPFNASFP RPTLNKSIGNGVEFLNRHLSAKLFHDKESMQPLLEFLRLHSYKGKTM MLNDKVQSLDSLQHVLRKAEEYLISVAPETPYSEFENRFREIGLERGWG DTAERVLEMIQLLLDLLEAPDPCTLETFLGRVPMVFNVVILSPHGYFAQ DNVLGYPDTGGQVVYILDQVRALENEMLNRIKKQGLDITPRILIITRLL PDAVGTTCGQRLERVYDTEYCDILRVPFRTEKGIVRKWISRFEVWPYLE TYTEDVALELAKELQAKPDLIVGNYSDGNIVASLLAHKLGVTQCTIAH ALEKTKYPESDIYWKKFEEKYHFSCQFTADLFAMNHTDFIITSTFQEIA GSKDTVGQYESHTAFTLPGLYRVVHGIDPFDPKFNIVSPGADMGIYFPY TETERRLTEFHSDIEELLYSSVENEEHICVLKDRNKPIIFTMARLDRVKNI TGLVEWYGKNARLRELVNLVVVAGDRRKESKDLEEKAEMKKMYGLI ETYKLNGQFRWISSQMNRVRNGELYRVICDTRGAFVQPAVYEAFGLT VVEAMTCGLPTFATCNGGPAEIIVHGKSGYHIDPYHGDHAAEILVEFF EKSKADPSHWDKISQGGLKRIHEKYTWQIYSDRLLTLTGVYGFWKHV TNLERRESKRYLEMFYALKYRKLVYFFHYYTIQWLHL	810
	GmSS4	Glyma.09G167000	MASTASNSALKRSDSITDSMPEALKQSRFHMKRCFARFVASGKRLMK QQHVMDDAEKTVEDKVERKKLLDGMLGYIFSCTQEAAVVPPYIAFAV RPNPGFWEYIKVNADDLQVEGIEAVEYLKYKEMIFDEKWANDENALE LDFGAIDFSTPRMVLSSSIGNGLNFTTKILTSRLSESSQNINPLLDYLLSL NYQGENLMIKDTLNTMPKLQQALKVAEAYVSALHKDTPYQKFEDRF KEWGFDKGWGNTAGRVKETMKLLSEVLESADPVKLESLFSRLPNMFN IVILSIHGYFGQADVLGLPDTGGQVVYILDQVRALEEELLHKIELQGLD VKPQILVVTRLIPDAKGTTCNQELEPVTHTKHSNILRVPFYTDKGMLH	921

Table S2. *Cont.*

			QWVSRFDIYPYLERFSQDATAKILELMEDKPDLIIGNYTDGNLVSSLMA SKLGVTQATIAHALEKTKYEDSDAKWMAFDEKYHFSCQFTADIISMN AADFIITSTYQEIAGSKQKPGQYETHTAFTMPGLCRAVSGINVFDPKFN IAAPGADQSVYFPSTEKEQRLIAFHPAIEELLFSKDDNEEHIGFLEDMK KPIIFSMARLDKVKNLSGLVEWYARNKRLRSLVNLVVVGGFFNPAKSK DREETEEIKKMHFLMKEYNLKGQFRWIAAQTDRYRNSELYRCISDSKG AFVQPALYEAFGLTVIEAMNCGLPTFATNQGGPAEIIVDEVSGFHIDPY NGDESSDKIADFFEKCKIDSEHWNRMSKAGLQRINECYTWKIYAKKV LNMGSIYGFWKRLNKEQKLAKERYNHMFYNLQFRNLAKQVPIPSERP QDPTQMPKPSAPAPSRRPAAKARPKKVSEHGIVGAPLTLLTAAATPKI KDHPTTSGEGVSARTATSEQSGGGGGLFGLRWLVSIISFLCAIHYLLKN LDRLFTREQ	
	GmSS5	Glyma.13G114000	MATDRLTRVHSLRERLDETLTANRNEILALLSRIEAKGKGILQHHQVI AEFEEIPEENRQKLTDGAFGEVLRSTQEAIVLPPWVALAVRPRPGVWE YLRVNVHALVVEELQPAEYLHFKEELVDGSSNGNFVLELDFEPFNAAF PRPTLNKSIGNGVQFLNRHLSAKLFHDKESLHPLLEFLRLHSVKGKTL MLNDRIQNPDALQHVLRKAEEYLGTVPPETPYSEFEHKFQEIGLERGW GDNAERVLESIQLLLDLLEAPDPCTLETFLGRIPMVFNVVILSPHGYFA QDNVLGYPDTGGQVVYILDQVRALENEMLHRIKQQGLDIVPRILIITRL LPDAVGTTCGQRLEKVFGTEHSHILRVPFRTEKGIVRKWISRFEVWPYL ETYTEDVAHELAKELQGKPDLIVGNYSDGNIVASLLAHKLGVTQCTIA HALEKTKYPESDIYWKKLEERYHFSCQFTADLFAMNHTDFIITSTFQEI AGSKDTVGQYESHTAFTLPGLYRVVHGIDVFDPKFNIVSPGADQTIYFP HTETSRRLTSFHPEIEELLYSSVENEEHICVLKDRSKPIIFTMARLDRVKN ITGLVEWYGKNAKLRELVNLVVVAGDRRKESKDLEEKAEMKKMYGLI ETYKLNGQFRWISSQMNRVRNGELYRVICDTRGAFVQPAVYEAFGLT VVEAMTCGLPTFATCNGGPAEIIVHGKSGFHIDPYHGDRAADLLVDFF EKCKLDPTHWDKISKAGLQRIEEKYTWQIYSQRLLTLTGVYGFWKHVS NLDRRESRRYLEMFYALKYRKLAESVPLAAE	805
	GmSS6	Glyma.14G209900	MASAPALKRTDSVVDNMPDALRQSRYHMKRCFAKYLGKGRRIMKLH HLMEEMELVIDDKSERSQVLEGILGFILSSTQEAVVDPPYVAFAIRPYPG VWEFVKVSSEDLSVEAITPTDYLKFKERVHDEKWATDENSFEADFGAF DFQIPQLTLSSSIGNGLQFTSKFLTSKLTGKLEKTQAIVDYLLTLNHQGE SLMINESLNSSAKLQMALVVADAFLSGLPKDTAYQNFELRFKEWGFER GWGDTAGRVKETMRTLSEVLQAPDPVNLEKFLSSLPIIFNVVIFSVHGY FGQADVLGLPDTGGQVVYILDQVKSLEAELLLRIKQQGLNVKPQILVV TRLIPDARGTKCHQELEPISDTKHSHILRVPFQTDKGILHQWISRFDIYP YLERFTQDATAKILEFMEGKPDLVIGNYTDGNLVASLMARKLGITQGT IAHALEKTKYEDSDVKWKELDPKYHFSCQFMADTVAMNASDFIITSTY QEIAGSKDRPGQYESHAAFTLPGLCRVVSGINVFDPKFNIAAPGADQS VYFPYTEKEKRLSQFHPAIEDLLFSKVDNIEHIGYLADRRKPIIFSMARL DVVKNLTGLVEWYGKNKRLRNLVNLVIVGGFFDPSKSKDREEMAEIK NMHDLIDKYQLKGQFRWIAAQTNRYRNGELYRCIADTRGAFVQPAL YEAFGLTVIEAMNCGLPTFATNQGGPAEIIVDGVSGFHIDPLNGDESS NKIADFFEKCKMNQSQWNVISAAGLQRINECYTWKIYANKMVNMG NIYTFWRQVNKEQKEAKQRYIQMFYNLIFKNLVKTVPVPSDEPQQPV GKQPSLKSRSTGRSHSRLQRLFGN	840

Table S2. *Cont.*

	GmSS7	Glyma.15G182600	MANHPLTHSHSFRERFDETLTGHRNEILALLSRLEAKGKGILQHHQVVAEFEEIPEESRKKLQGGVFGEVLRSTQEAIVLPPFVALAVRPRPGVWEYLRVNVHMLVVDELLPAEYLRFKEELVEGSSNGNFVLELDFEPFNASFPRPTLNKSIGNGVEFLNRHLSAKLFHDKESMQPLLEFLRLHSYKGKTMMLNDKVQSLDSLQHVLRKAEEYLTSVAPETPYSEFENKFREIGLERGWGDIAERVLEMIQLLLDLLEAPDPCTLETFLGRVPMVFNVVILSPHGYFAQDNVLGYPDTGGQVVYILDQVRALENEMLNRIKKQGLDITPRILIITRLLPDAVGTTCGQRLERVYDTEYCDILRVPFRTEKGIVRKWISRFEVWPYLETYTEDVALELAKELQAKPDLIVGNYSDGNIVASLLAHKLGVTQCTIAHALEKTKYPESDIYWKKFEEKYHFSCQFTADLFAMNHTDFIITSTFQEIAGSKDTVGQYESHTAFTLPGLYRVVHGIDPFDPKFNIVSPGADMSIYFPYTETERRLTEFHPDIEELLYSSVENEEHICVLKDRNKPIIFTMARLDRVKNITGLVEWYGKNARLRELVNLVVVAGDRRKESKDLEEKAEMKKMYGLIETYKLNGQFRWISSQMNRVRNGELYRVICDTRGAFVQPAVYEAFGLTVVEAMTCGLPTFATCNGGPAEIIVHGKSGYHIDPYHGDRAAEILVEFFEKSKADPSHWDKISQGGLKRIHEKYTWQIYSDRLLTLTGVYGFWKHVTNLERRESKRYLEMFYALKYRKLAESVPLAIEE	806
	GmSS8	Glyma.15G151000	MPATSVRERVLDTLSRYRNEFISLLSRYVAGGKGILQPHDLLYEVEKLLEEDEGMQKLKDSPFVKELESAKEAIVLPPFVSIALRPRPGVWEYVRVDAFELSVDNLSVAEYLRLKEELVDGQCTDKYVLELDFEPFNVTLPRPTRSSSIGDGVQFLNRHLSSFMFRSKESLEPLLAFLRTHRYDGHAMMLNDRIYNLSKLQSSLAKAEELLSRLLPNAPYSDFEYELQGLGFERGWGDTAERVSEMVHLLLEILQAPDPNTLESFLGRIPMVFNVVVVSPHGYFGQANILGLPDTGGQLVYILDQVRALENEMLIKIQKQGLDVSPKILIVTRLIPEAKGTTCNQRLERVSGTEHSYILRVPFRTKNGILRKWISRFDMWPYLETFAEDASHEIAGELQGIPDLIIGNCSDGNLVATLLSYKLGITQCNIAHALEKTKHPDSDIYWKKYEDKYHFTCQFTADLIAMNNADFIITSTYQEIAGSKNNVGQYESYTAFTLPGLYRVVHGIDVFDPKFNIVSPGADMCIYFPYSDRERRLTSLHGSIEKLVYGAEQNEEHIGLLNDRSKPIIFSMARIDPVKNITGLVECFGKSSKLRELVNLVVVGGYIDVQKSTDIEEMREIEKMHNLIEEYNLHGQFRWIKAQMNRARNGELYRYIADVKGAFVQPALYEAFGLTVVEAMTCGLPTFATCHGGPAEIIEHGVSGFHIEPHHPDHVAAILINFFEQCQSDPGYWNKISDAGLRRIHERYTWKIYSERLLTLAGVYGFWKHVSKLEKRETRRYLEMFYILKFRDLVKSIPLAVN	802
	GmSS9	Glyma.16G217200	MASTAPNSALKRSDSITDSMPEALKQSRFHMKRCFARFVASGKRLMKQQHVMDDVEKTVEDKAERKKFLDGMLGYIFSCTQEAAVVPPYVAFAVRPNPGFWEYIKVNADDLQVEGIEAVEYLKYKEMIFDEKWANDENALELDFGAIDFSTPQMVLSSSIGNGLNFTTKILTSRLSGSSQSINPLLDYLLSLNYQGENLMIKDTLNTMPKLQQALKVAEAYVSALNKDTAYQKFEDRFKEWGFDKGWGNTAGRVKETMKLLSEVLESADPVKLESLFSRLPNMFNIVILSIHGYFGQADVLGLPDTGGQVVYILDQVRALEEELLHKIELQGLDVKPQILVVTRLIPDAKGTTCNQELEPVTNTKHSNILRVPFYTDKGMLRQWVSRFDIYPYLERFSQDATAKIFDLMEDKPDLIIGNYTDGNLVSSLMASKLGVTQATIAHALEKTKYEDSDAKWMAFDEKYHFSCQFTADIISMNAADFIITSTYQEIAGSKQKPGQYETHTAFTMPGLCRAVSGINVFDPKFNIAAPGADQSVYFPSTAKEQRLTSFHPAIEELLYSKDDNEEHIGLLEDMKKPIIFSMARLDKVKNLSGLVEWYARNKRLRSLVNLVVVGGFFNPAKSKDREETEEIKKMHFLMKEYNLKGQFRWIAAQTDRYRNSELYRCISDTKGAFVQPALYEAFGLTVIEAMNCGLPTFATNQGGPAEIIVDGVSGFHIDPYNGDESSDKIADFFEKCKTDSQHWNRMSKAGLQRINECYTWKIYAKKVLNMGSIYGFWRRLNREQKLAKERYIHMFYNLQFRNLAKQVPIPSETP	920

Table S2. *Cont.*

			QDPTQMPKPSAPAPSRRSAAKARPKKVSEHWIVGAPLTLLTAAATPKI<br>KDHPTPSGEGVSEGTATSEQSGGGGLFGLRWLVPIIAFVCAIHYFLKNL<br>DRLFTREQ	
	GmSS10	Glyma.17G0<br>45800	MATDRLTRVHSLRERLDETLTANRNEILALLSRIEAKGKGILQHHQVI<br>AEFEEIPEENRQKLTDGAFGEVLRSTQEAIVLPPWVALAVRPRPGVWE<br>YLKVNVHALVVEELQPAEYLHFKEELVDGSSNGNFVLELDFEPFNAAF<br>PRPTLNKSIGNGVQFLNRHLSAKLFHDKESLHPLLEFLRLHSVKGKTL<br>MLNDRIQNPDALQHVLRKAEEYLGTVPPETPYSEFEHKFQEIGLERGW<br>GDNAERVLESIQLLLDLLEAPDPCTLETFLGRIPMVFNVVILSPHGYFA<br>QDNVLGYPDTGGQVVYILDQVRALENEMLHRIKQQGLDIVPRILIITRL<br>LPDAIGTTCGQRLEKVFGTEHSHILRVPFRTEKGIVRQWISRFEVWPYL<br>ETYTEDVAHELAKELQGKPDLIVGNYSDGNIVASLLAHKLGVTQCTIA<br>HALEKTKYPESDIYWKKLEERYHFSCQFTADLFAMNHTDFIITSTFQEI<br>AGSKDTVGQYESHTAFTLPGLYRVVHGIDVFDPKFNIVSPGADQTIYFP<br>PTETSRRLTSFHPEIEELLYSSVENEEHICVLKDRSKPIIFTMARLDRVKNI<br>TGLVEWYGKNAKLRELVNLVVVAGDRRKESKDLEEKAEMKKMYGLI<br>ETYKLNGQFRWISSQMNRVRNGELYRVICDTKGAFVQPAIYEAFGLTV<br>VEAMTCGLPTFATCNGGPAEIIVHGKSGFHIDPYHGDRAADLLVDFFE<br>KCKLDPTHWETISKAGLQRIEEKYTWQIYSQRLLTLTGVYGFWKHVSN<br>LDRRESRRYLEMFYALKYRKLAESVPLAVE	805
	GmSS11	Glyma.<br>19G212800	MSTQPKLGRIPSIRDRVEDTLSAHRNELISLLSRYVAQGRGILQPHNLID<br>ELDNIPGDDQAIVDLKNGPFGEIVKSAKEAIVLPPFVAIAVRPRPGVWE<br>YVRVNVSELSVEQLSVSEYLSFKEELVDGKINDNFVLELDFEPFNATFPR<br>PTRSASIGNGVQFLNRHLSSIMFRNKDSLQPLLDFLRAHKYKGHALML<br>NDRIQTISKLQSALAKAEDYLSKLAHDTLYSEFEYVLQGMGFERGWGD<br>TAERVLEMMHLLLDILQAPDPSTLETFLGRVPMVFNVAILSPHGYFGQ<br>ANVLGLPDTGGQVVYILDQVRALENEMLLRIKKQGLDFTPRILIVTRLI<br>PDAKGTTCNQRLERVSGTDHTHILRVPFRSESGTLRKWISRFDVWPYLE<br>TYAEDVASEIAAELQGYPDFIIGNYSDGNLVASLLAYKMGVTQCTIAH<br>ALEKTKYPDSDLYWKKFEDKYHFSCQFTADLIAMNNADFIITSTYQEIA<br>GTKNTVGQYESHAGFTLPGLYRVVHGIDVFDPKFNIVSPGADMSIYFP<br>YSEKQNRLTALHGSIEQLLFAPEQTDEYIGLLKDKSKPIIFSMARLDRVK<br>NITGLVESFGKNSKLRELVNLVIVAGYIDVKKSSDREEIAEIEKMHELM<br>KKYNLVGDFRWIAAQTNRARNGELYRYIADTQGAFVQPAFYEAFGLT<br>VVEAMNCGLPTFATCHGGPAEIIEHGISGFHIDPYHPDQASQLLVEFF<br>QKSKEDPSHWKKISDGGLQRIYERYTWKIYSERLMTLAGVYSFWKYVS<br>KLERRETRRYLEMFYILKFRDLANSVPLAKDDAS	812
Malus domestica	MdSS7	MDP000025<br>0070	MAERVLTRVQSLRERLDETLSAHRNEILALLSRIESKGKGFLQPHQLLA<br>EFEEIPEANRQKLLDGAFGEVLKNTQEVIVLPPWVALAVRPRPGVWEY<br>IRVNVHALVVEELRVAEYLQFKEELVDGSANGNFVLELDFEPFNASFP<br>RPTLSKSIGNGVEFLNRHLSAKLFHDKESLHPLLKFLRVHCYEGRNLM<br>LNNKIKDVNELQHVLRKAEDFLSAIAPETPYKDFEPKLQAIGLERGWG<br>DTAGRVLEMIELLLDLLEAPDPCTLEKFLDQIPMVFNVVILSPHGYFAQ<br>DNVLGYPDTGGQVVYILDQVRALEAEMLKRVKQQGLDIIPRIIILTRLL<br>PDAVGTTCGDRLEKVYGTEHSDILRVPFRDEKGAIVRRWISRFEVWPY<br>LETYAEDAIKELSKEMHGKPDLIIGNYSDGNIVASLMALKLGVTQCTIA<br>HALEKSKYPDSDLYWKKLDDSYHFSCQFTADLIAMNHTDFIITSTYQEI<br>AGSKETVGQYESHTAFTLPGLYRVVHGIDVFDPKFNIVSPGADMSVYF<br>PYSEKEKRLTNFHPEIEELLYSQVENKEHLCVLKDRNKPIIFTMARLDR<br>VKNITGLVEWYGKNAKLRELVNLVVVAGDRRKESKDNEEKAEMKK<br>MYELIDTYKLNGQFRWISSQMNRVRNGELYRYICDTKGAFVQPAVYE	807

Table S2. *Cont.*

			AFGLTVVEAMTCGLPTFATCKGGPAEIIVHGKSGYHIDPYHGDQAAEI<br>LVDFFEKSKADPSHWDKISQGGLQRIYEKYTWQIYSQRLLTLTGVYGF<br>WKHVSNLDRLESRRYLEMFYALKYSKLAASVPLAVEE	
	MdSS4	MDP000087<br>2262	MAERVLTRVQSLRERLDETLSTQRNDFLALLSRIESKGKGFLQPHQLLA<br>EFEAIPENNRQKLLDGAFGEVLKHTQEAIVLPPWVALAVRPRPGVWE<br>YIRVNVHALALEELCVAEYLQFKEELVDGSSNGNFVLELDFQPFNASF<br>PRPTLSKSIGNGVEFLNRHLSAKLFHDKESLHPLLEFLRVHCYEGRNM<br>MLNNRIKNVNELQRVLRKAEDFLSSIVPRTPYKEFEPKLQAIGLERGW<br>GDTAERVLEMIQLLLDLLEAPNPFTLEKFLGQIPMVFNVVILSPHGYFA<br>QDHVLGFPDTGGQVVYILDQVRALESEMLKRIKQQGLDITPRIIILTRLL<br>PDAVGTTCGDRLEKVYGCEHSDILRVPFRDKKGAIVRRWISRFEVWPY<br>LETYAGDAITELSKEMQGKPDLIIGNYSDGNIVASLMAHXLGVTQCTI<br>AHALEKSKYPDSDLYWKKLDENYHFSCQFTADLIAMNHTDFIITSTFQ<br>EIAGSKDTVGQYESHTAFTLPGLYRVVHGIDVFDPKFNIVSPGADMNV<br>YFPFSEKEKRLTHFHPEIEELLYGQVENIEHLCVLKDRNKPIIFTMARLD<br>RVKNITGLVEWYGKNAKLRELVNLVVVAGDRRNESKDNEEKAELKK<br>MYEXIDTYKLNGQLRWISSQMNRVRNGELYRYICDTRGAFVQPAVYE<br>AFGLTVIEAMTCGLPTFATCKGGPAEIIVNGKSGYHIDPYHGDQAAEI<br>XVDFFEKNKADPSHWDKISQGGLQRIYEKYTWQIYSERLLTLTGVYGF<br>WKDVSNLDRLESRRYLEMFYALKFRKLAASVPLAVEE	807
	MdSS2	MDP000016<br>0578	MAERALTRVQSLRERLDETLXTHRNEILALLSRIESKGKGFLQPHQLHA<br>EFEAIPENNRQKLLDGAFGEVLKHTQEAVVLPPWVAFAVRPRPGVWE<br>YIRVNVHALVLEELRVAEYLQFKEELVDGSSNGNFVLELDFQPFHASFP<br>RPTLSKSIGKGVEFLNRHLSAKLFHDKESLHPLLEFLRVHCYEGRNMM<br>LNNRIKNVNELERVLRKAEDFLSSXVPGTPYKEFEPKXQXIGLERGWG<br>DTAERVLEMIQLLLDLIEAPDPFTLEKFLGQIPMVFNVVILSPHGYFAQ<br>DHVLGFPDTGGQVVYILDQVRALESEMLKRIKQQGLDITPRIIILTRLLP<br>DAVGTTCGDRLEKVYGCEHSDILRVPFRDEKGXIVRRWISRFEVWPYL<br>QTYAEDAIKELSKEMQGKPDLIIGNYSDGNIVASLMAHKLGVTQCTIA<br>HALEKSKYPDSDLYWKKLDENYHFSCQFTADLIAMNHTDFIITSTFQEI<br>AGSKDTVGQYESHTAFTLPGLYRVVHGIDVFDPKFNIVSPGADKDVYF<br>PYSEKEKRLTHFHPEIEELLYGQVENIEQLCVLKDRNKPIIFTMARLDRV<br>KNITGLVEWYGKNAKLRELVNLLVVAGDRRNESKDNEEKAELKKMY<br>ELIDTYKLNGQLRWISSQMNRVRNGELYRYICDTRGAFVQPAVYEAFG<br>XTVIEAMTCGLPTFATCKGGPAEIIVNGISGYHIDPYHGDQAAEILVDF<br>FEKSKADPSHWDKISQGGLQRIYEKYTWQIYSERLLTLTGVYGFWKDV<br>SNLDRLECRRYLEMFYALKFRKLAASVPLAVE	806
	MdSS8	MDP000013<br>2527	MAERALTRVQSLRERLDETLXTHRNEILALLSRIESKGKGFLQPHQLHA<br>EFEAIPENNRQKLLDGAFGEVLKHTQEAVVLPPWVAFAVRPRPGVWE<br>YIRVNVHALVLEELRVAEYLQFKEELVDGSSNGNFVLELDFQPFHASFP<br>RPTLSKSIGKGVEFLNRHLSAKLFHDKESLHPLLEFLRVHCYEGRNMM<br>LNNRIKNVNELERVLRKAEDFLSSXVPGTPYKEFEPKXQXIGLERGWG<br>DTAERVLEMIQLLLDLIEAPDPFTLEKFLGQIPMVFNVVILSPHGYFAQ<br>DHVLGFPDTGGQVVYILDQVRALESEMLKRIKQQGLDITPRIIILTRLLP<br>DAVGTTCGDRLEKVYGCEHSDILRDAIXELSKEMQGKPDLIIGNYSDG<br>NIVASLMAHXLGVTQCTIAHALEKSKYPDSDLYWKKLDENYHFSCQF<br>TADLIAMNHTDFIITSTFQEIAGSKDTVGQYESHTAFTLPGLYRVVHGI<br>DVFDPKFNIVSPGADXXVYFPXSEKEKRLTHFHPEIEELLYGQVENIEXL<br>CVLKDRNKPIIFTMARLDRVKNITGLVEWYGKNAKLRELVNLVVVAG<br>DRRNESKDNEEKAELKKMYEXIDTYKLNGQLRWISSQMNRVRNGELY<br>RYICDTRGAFVQPAVYEAFGLTVIEAMTCGLPTFATCKGGPAEIIVNGK	778

Table S2. *Cont.*

			SGYHIDPYHGDQAAEIXVDFFEKNKADPSHWDKISQGGLQRIYEKYT WQIYSERLLTLTGVYGFWKDVSNLDRLESRRYLEMFYALKFRKLAASV PLAVEE	
	MdSS5	MDP0000126946	MANRPKFTRALSLRERVEDTLSDHRNELVALLSRYLDQGKRILQPHDL IDQLDIVIGDDEAKRQLKTGPFSEVLKSAQEAIILPPYVALAVRPRPGV WDYVRVNVYELSVEELTVSEYLRFKEELVDGESSDKYALELDFEPFNA AFPRPTRSSSIGNGVQFLNRHLSSIMFRNKESLEPLLDFLKAHKYKGHP LMLNDRIQSVSKLQSALAKAEDHLSKLQPETPYSEFEYLFQGMGFERG WGDTAVHVLEMMHLLLDILQAPDPSILETFLGRIPMMFNVVILSPHGY FGQANVLGLPDTGGQASQYSSFILVLFHYLFYLYSGGIVIFYXEIVYILDQ VRALEKEMLERIRLQGLDFTPRILIVTRLIPEAKGTTCNQRLERISGTEHT HILRVPFRSEKGILRKWISRFDVWPYLETFAEDAAGEIIAELQGYPDFIIG NYSDGNLVASLLAYKMGVTQCTIAHALEKTKYPDSDIYWKKFEEKYH FSTQFTADLIAMNNADFIITSTYQEIAGTKDTVGQYESHSSYTLPGQYR VVHGINVFDPKFNIVSPGADMAIYFPYSEKQKRLTSLHGSLEELLYNPD QNDVHIGTLSDRSKPIIFSMARLDQVKNMTGLVECYAKCSKLRDLANL VIVAGYIDAKKSQDREEIAEIEKMHNLMTEYKLDGQFRWISXQTNRVS NGELYRYIADTRGAFAQPAFYEAFGLTVVEAMTCGLPTFATVHGGPA EIIEHGVSGFHIDPYHPEKAAALMADFFQRCKEDPSYWNTISDAGLQRI YEKYTWKIYSERLMTLAGVYGFWKYVSKLERRETRRYLEMFYILKFRDL AKSVPEAIDDAH	841
	MdSS1	MDP0000252802	MAERALTRVQSLRERLDETLFTHRNEILALLSRIESKGKGFLQPHQLHA EFEAIPENNRQKLLDGAFGEVLKHTQEAVVLPPWVAFAVRPRPGVWE YIRVNVHALVLEELRVAEYLQFKEELVDGSSNGNFVLELDFQPFHASFP RPTLSKSIGKGVEFLNRHLSAKLFHDKESLHPLLEFLRVHCYEGRNMM LNNRIKNVNELERVLRKAEDFLSSVVPGTPYKEFEPKMQVIGLERGWG DTAERVLEMIQLLLDLIEAPDPFTLEKFLGQIPMVFNVVILSPHGYFAQ DHVLGFPDTGGQVVYILDQVRALESEMLKRIKQQGLDITPRIIILTRLLP DAVGTTCGDRLEKVYGCEHSDILRVPFRDEKGAIVRRWISRFEVWPYL QTYAEDAIKELSKEMQGKPDLIIGNYSDGNIVASLMAHKLGVTQCTIA HALEKSKYPDSDLYWKKLDENYHFSCQFTADLIAMNHTDFIITSTFQEI AGSKDTVGQYESHTAFTLPGLYRVVHGIDVFDPKFNIVSPGADKDVYF PYSEKEKRLTHFHPEIEELLYGQVENIEQLCVLKDRNKPIIFTMARLDRV KNITGLVEWYGKNAKLRELVNLLVVAGDRRNESKDNEEKAELKKMY ELIDTYKLNGQLRWISSQMNRVRNGELYRYICDTRGAFVQPAVYEAFG LT	679
	MdSS6	MDP0000212593	MASTSPPSKRSDTIAETMPEALRESRFHMKKCFASFGTGKRLMKPQHI MEELEKSIEDRHERSKVLEGLLGYILSRTQEAAVVPPYVAFAVRPNPGF SEFVKVNADDLTVDGISATEYLKFKEMIFDESWANDENALELDFGAID FSTPRMTLPSSIGNGLNFVLKSISSRLSTHASCSDYAKSLLDYLLRLNYH GENLMINESLDTVAKLQTALIQAEVLVSTLPKTTPFPSFEQRLKVLGFE KGWGDTAERVGETMRLLSEVFQAPDSVMLESLFSRLPNTFNIVIFSPHG YFGQSDVLGLPDTGGQVVYILDQVRALEEELLLRIKQQGLAVKPQILV VTRLIPDARGTKCNQELEAIIDTKHSHILRVPFRTDKGVLHQWDATSKI LQHMECKPDLIIGNYSDGNLVASLIASKLGITQGTIAHALEKTKYEDSD AKWKEFDPKYHFSCQFTADIISMNCADFVITSTFQEIAGGKDRPGQYES HTAFTMPGLYRVVSGIDVFDPKFNIAAPGADQSVYFPYSEKQRRFTKF QPAIEELLYTKEENDEHIGFLADKKKPIIFSMARLDTVKNLTGLVEWFG KNKRLRNSVNLVIVGGFFDPSKSKDREEIAEIKKLHALVQAYQLSGQFR WIAAQTDRYRNGELYRCIADTKGAFVQPALYEAFGLTVIEAMNCGLP TFATNQGGPAEIIVDGVSGFHIDPNNGDESSNKIADFFEKCKTDGEYW	883

Table S2. *Cont.*

			KKMSAAGLQRINECYTWKIYANKMLNMGSTYGFWRQLRDDQKLAK<br>ETYIHMFYNLLFRKLAKNVAVPSVGYEKPAPRAVTAAVDQPTTAAAS<br>KPPQPPAAPTSAVPQLTPRVRDEGRELSQPRSRSRARCLWNCCCVILGF<br>LIIXYYKIRNMYN	
	MdSS3	MDP000013<br>8004	MASAAAIKGSESIADNMPDARRQSRYHMKRCFAKYXEKGRRIIKLHH<br>LMSEMETVIDDKAERTQVLEGVLGYILCSTQEAVVIPPHVVFSIRPNPG<br>YWEFVKVSSEDLSIEGITVRDFLKYKETLYDENWSNDENALEVDFRAID<br>FSTPHLTLSSSIGNGINYVTKFTTSKLAGKLENAQPLVDYLLSLNHQGE<br>QLILNETLNTASKLQAALIVTDVHLSALPKDTPFQNFELRFKEWGFEK<br>GWGDTAERTKETMGILAEVLQAPDPLIMDRFFSRLPTIFNVVIFSPHGY<br>FGQADVLGLPDTGGQVVYILDQVKALEEELLVRIKQQGLTVKPQILVV<br>TRLIPEARGTKCNQELEPINGTKYSNILRVPFRTEKGILRRWDATAKILD<br>LMEGKPDLIIGNYTDGNLVASLMANKLGITQATIAHALEKTKYEDSDI<br>NWKXLDPKYHFSCQFLADTISMNATDFVIASTYQEIAGSKDRPGQYES<br>HTAFTXPGLCRVVSGINVFDPKFNIAAPGADQSVYFPYAEKQKRLTSF<br>HPAIEELLFSKEDXNEHIGFLVDRKKPIIFSMARLDIVKNITGLVEWYGK<br>NKRLXNLVNLVVVGGFFDPSKSKDREEIAEIKKMHTLIEKYXLRGQIR<br>WIAAQTDRNRNGELYRCIADTRGAFVQPALYEAFGLTVIEAMNCGLP<br>TFATNQGGPAEIIVDGISGFHIDPNNGDEASNKIADFFEKSKTDAAYW<br>DRISKAGLQRIYECYTWKIYANKVLNMGSTYTFWRQLNKEQKQAKQR<br>YIQMFFSLQYRNLVKNVPIPSDETEQPVVPKPTAKPKTTLRHVQLS	819
	MdSS9	MDP000027<br>7711	SNDEHVLEVDFRAIDFSTPHLTLSSSVGNGIDYVTKFTTSRLAGKLENA<br>QPLADYLLSLNHQGEQLILNETLNTASKLQAALIVTEVYLSDLPKDTPF<br>QNFELSFKEWGFEKGWGDTAERTKETMKILLEVLQAPDPLNMDRFFS<br>RLPTIFNVVIFSPHGYFGQADVLGLPDTGGQVVYILDQVKAMEEELTL<br>RIKQQGLTVKPQILVVTRLIPEARGTKCNQELEPINGTKYSNILRVPFRT<br>EKGILRRWVSRFDIYPYLELFTQACFPSIFKFILLECSIIDFVPYDATAKIL<br>NLMEGKPDLIIGNYTDGNLVASLMANKLGITQATIAHALEKTKYEDS<br>DINWKELDPKYHFSCQFLADTISMNATDFVIASTYQEIAGSKDRPGQY<br>ESHTAFTLPGLCRVVSGINVFDPKFNIAAPGADQSVYFPYSDKXKRLTS<br>FYPAIEELLFSKEDXSEHLGFLVDRKKPIIFSMARLDIVKNITGLVEWYG<br>KNKRLRNLVNLVVVGGFFDPSKSKDREEIAEIKKMHTLIEKYQLRGQIR<br>WIAAQTDRNRNGELYRCIADTRGAFVQPALYEAFGLTVIEAMNCGLP<br>TFATNQGGPAEIIVDGVSGFHIDPNNGDEASKKIADFFENSKTDVAYW<br>DRFSKAGLQRIYECYTWKIYANKVLNMGSTYTFWRQLNKEQKQAKQ<br>RYIQMFFNLQYRNXVKNVPVPXDEPEQPQTTSRHHKALTVSIAKVVEV<br>VHHYDKDCIPLKYADNMLAYIQSSKTNKTCIRRLTIPKQMKSPVYIYY<br>QLDHFYQNHRRYVKSRSDKQLRSKSNENKTDDCAPERYTTKGVIVPC<br>GLVAWSLFNDTYKFSVNNKQLGVSKKDITWKSDQKNKFGSDVYPKN<br>FQSEGLIGGAKLNSSIPLSEQEDLMVWMRTAALPTFRKLYGRIEVDLEA<br>DAVVTVTIENNYNTYSFRGNKKLVLSTASWIGGKNYLLGVAYLTVGG<br>LCLFLALAFLLLYLIKPRYVNYFTGHTVCNDQLKLDSILDNYGKFTLVF<br>LLKWINSSTKPIFSGLLSLYIEHNRQCCIYAQAXTEMCLKLFRPFCAGLL<br>GIYRTCLGTGVHQEDTYTRSPSMHLSIRFDLFWDNLFCH	1102
	MdSS10	MDP000019<br>5934	MASAAAIKRSDSIADTMPDALRQSRYHMKRCFAKYIEQGRRIMKLHH<br>LMSEMETVIDDKAERTQVLGGVLGYILCSTQEAVVVPPHVVFSIRLNP<br>GYWEFVKVSSEDLSVEAITVRDFLKYKEALYDEKWSNDEHVLEVDFRA<br>IDFSTPHLTLSSSIGNGIEYVTKFTTSKLAGKLENAQPLVDYLLXLNHQG<br>EQLILNETLNTASKLQAALIVTEVYLSTLPKDTPFQNFELRFKEWGFEK<br>GWGDTAERTKETMKILSEVLQAPDPLNMDRFFSRLPIIFNVVIFSPHGY<br>FGQADVLGLPDTGGQVVYILDQVKAMEEELTLRIKQQGLTVKPQILVV	502

Table S2. *Cont.*

			TRLIPEARGTKCNQELEPIYGTKYSNILRVPFRTEKGILRRWDATAKILD<br>LMEGKPDLIIGNYTDGNLVASLMANKLGITQATIAHALEKTKYENSDI<br>NWKELDPKYHFSCQFLADTISMNATDFVIASTYQEIAGSKDRPGQYES<br>HTAFTLPGLCRVVSGIN	
Solanum tuberosum	StSS1	M18745	MAERVLTRVHSLRERVDATLAAHRNEILLFLSRIESHGKGILKPHELLA<br>EFDAIRQDDKNKLNEHAFEELLKSTQEAIVLPPWVALAIRLRPGVWEY<br>IRVNVNALVVEELSVPEYLQFKEELVDGASNGNFVLELDFEPFTASFPK<br>PTLTKSIGNGVEFLNRHLSAKMFHDKESMTPLLEFLRAHHYKGKTMM<br>LNDRIQNSNTLQNVLRKAEEYLIMLPPETPYFEFEHKFQEIGLEKGWG<br>DTAERVLEMVCMLLDLLEAPDSCTLEKFLGRIPMVFNVVILSPHGYFA<br>QENVLGYPDTGGQVVYILDQVPALEREMLKRIKEQGLDIIPRILIVTRLL<br>PDAVGTTCGQRIEKVYGAEHSHILRVPFRTEKGIVRKWISRFEVWPYM<br>ETFIEDVAKEISAELQAKPDLIIGNYSEGNLAASLLAHKLGVTQCTIAH<br>ALEKTKYPDSDIYWKKFDEKYHFSSQFTADLIAMNHTDFIITSTFQEIAG<br>SKDTVGQYESHMAFTMPGLYRVVHGINVFDPKFNIVSPGADINLYFSY<br>SETEKRLTAFHPEIDELLYSDVENDEHLCVLKDRTKPILFTMARLDRVK<br>NLTGLVEWYAKNPRLRGLVNLVVVGGDRRKESKDLEEQAEMKKMYE<br>LIETHNLNGQFRWISSQMNRVRNGELYRYIADTKGAFVQPAFYEAFGL<br>TVVEAMTCGLPTFATNHGGPAEIIVHGKSGFHIDPYHGEQAADLLAD<br>FFEKCKKDPSHWETISMGGLKRIEEKYTWQIYSESLLTLAAVYGFWKH<br>VSKLDRLEIRRYLEMFYALKYRKMAEAVPLAAE	805
	StSS2	AY205084	MAERVLTRVHSLRERLDATLAAHRNEILLFLSRIESHGKGILKPHQLLA<br>EFESIHKEDKDKLNDHAFEEVLKSTQEAIVLPPWVALAIRLRPGVWEY<br>VRVNVNALIVEELTVPEFLQFKEELVNGTSNDNFVLELDFEPFTASFPK<br>PTLTKSIGNGVEFLNRHLSAKMFHDKESMTPLLEFLRVHHYKGKTMM<br>LNDRIQNLYTLQKVLRKAEEYLTTLSPETSYSAFEHKFQEIGLERGWGD<br>TAERVLEMICMLLDLLEAPDSCTLEKFLSRIPMVFNVVILSPHGYFAQE<br>NVLGYPDTGGQVVYILDQVPALEREMLKRIKEQGLDIKPRILIVTRLLP<br>DAVGTTCGQRLEKVFGTEHSHILRVPFRTEKGIVRKWISRFEVWPYME<br>TFIEDVGKEITAELQAKPDLIIGNYSEGNLAASLLAHKLGVTQCTIAHA<br>LEKTKYPDSDIYLNKFDEKYHFSAQFTADLIAMNHTDFIITSTFQEIAGS<br>KDTVGQYESHMAFTMPGLYRVVHGIDVFDPKFNIVSPGADVNLYFPY<br>SEKEKRLTTFHPEIEDLLFSDVENEEHLCVLKDRNKPIIFTMARLDRVK<br>NLTGLVEWYAKNPRLRELVNLVVVGGDRRKESKDLEEQAEMKKMYE<br>LIKTHNLNGQFRWISSQMNRVRNGELYRYIADTRGAFVQPAFYEAFGL<br>TVVEAMSCGLPTFATNQGGPAEIIVHGKSGFQIDPYHGEQAADLLADF<br>FEKCKVDPSHWEAISEGGLKRIQEKYTWQIYSDRLLTLAAVYGFWKHV<br>SKLDRLEIRRYLEMFYALKFRKLAQLVPLAVE	805
	StSS3	STU24088	MAERVLTRVHSLRERLDATLAAHRNEILLFLSRIESHGKGILKPHQLLA<br>EFESIHKEDKDKLNDHAFEEVLKSTQEAIVLPPWVALAIRLRPGVWEY<br>VRVNVNALIVEELTVPEFLQFKEELVNGTSNDNFVLELDFEPFTASFPK<br>PTLTKSIGNGVEFLNRHLSAKMFHDKESMTPLLEFLRVHHYKGKTMM<br>LNDRIQNLYTLQKVLRKAEEYLTTLSPETSYSAFEHKFQEIGLERGWGD<br>TAERVLEMICMLLDLLEAPDSCTLEKFLGRIPMVFNVVILSPHGYFAQE<br>NVLGYPDTGGQVVYILDQVPALEREMLKRIKEQGLDIKPRILIVTRLLP<br>DAVGTTCGQRLEKVFGTEHSHILRVPFRTEKGIVRKWISRFEVWPYME<br>TFIEDVGKEITAELQAKPDLIIGNYSEGNLAASLLAHKLGVTQCTIAHA<br>LEKTKYPDSDIYLNKFDEKYHFSAQFTADLIAMNHTDFIITSTFQEIAGS<br>KDTVGQYESHMAFTMPGLYRVVHGIDVFDPKFNIVSPGADVNLYFPY<br>SEKEKRLTTFHPEIEDLLFSDVENEEHLCVLKDRNKPIIFTMARLDRVK<br>NLTGLVEWYAKNPRLRELVNLVVVGGDRRKESKDLEEQAEMKKMYE	805

Table S2. *Cont.*

			LIKTHNLNGQFRWISSQMNRVRNGELYRYIADTRGAFVQPAFYEAFGL<br>TVVEAMSCGLPTFATNQGGPAEIIVHGKSGFQIDPYHGEQAADLLADF<br>FEKCKVDPSHWEAISEGGLKRIQEKYTWQIYSDRLLTLAAVYGFWKHV<br>SKLDRLEIRRYLEMFYALKFRKLAQLVPLAVE	
	StSS4	STU24087	MAERVLTRVHSLRERVDATLAAHRNEILLFLSRIESHGKGILKPHELLA<br>EFDAIRQDDKNKLNEHAFEELLKSTQEAIVLPPWVALAIRLRPGVWEY<br>IRVNVNALVVEELSVPEYLQFKEELVDGASNGNFVLELDFEPFTASFPK<br>PTLTKSIGNGVEFLNRHLSAKMFHDKESMTPLLEFLRAHHYKGKTMM<br>LNDRIQNSNTLQNVLRKAEEYLIMLPPDTPYFEFEHKFQEIGLEKGWG<br>DTAERVLEMVCMLLDLLEAPDSCTLEKFLGRIPMVFNVVILSPHGYFA<br>QENVLGYPDTGGQVVYILDQVPALEREMLKRIKEQGLDIIPRILIVTRLL<br>PDAVGTTCGQRIEKVYGAEHSHILRVPFRTEKGIVRKWISRFEVWPYM<br>ETFIEDVAKEISAELQAKPDLIIGNYSEGNLAASLLAHKLGVTQCTIAH<br>ALEKTKYPDSDIYWKKFDEKYHFSSQFTADLIAMNHTDFIITSTFQEIAG<br>SKDTVGQYESHMAFTMPGLYRVVHGINVFDPKFNIVSPGADINLYFSY<br>SETEKRLTAFHPEIDELLYSDVENDEHLCVLKDRTKPILFTMARLDRVK<br>NLTGLVEWYAKNPRLRGLVNLVVVGGDRRKESKDLEEQAEMKKMYE<br>LIETHNLNGQFRWISSQMNRVRNGELYRYIADTKGAFVQPAFYEAFGL<br>TVVEAMTCGLPTFATNHGGPAEIIVHGKSGFHIDPYHGEQAADLLAD<br>FFEKCKREPSHWETISTGGLKRIQEKYTWQIYSERLLTLAAVYGFWKHV<br>SKLDRLEIRRYLEMFYALKYRKMAEAVPLAAE	805
	StSS5	AJ537575	MAERVLTRVHSLRERVDATLAAHRNEILLFLSRIESHGKGILKPHELLA<br>EFDAIRQDDKNKLNEHAFEELLKSTQEAIVLPPWVALAIRLRPGVWEY<br>IRVNVNALVVEELSVPEYLQFKEELVDGASNGNFVLELDFEPFTASFPK<br>PTLTKSIGNGVEFLNRHLSAKMFHDKESMTPLLEFLRAHHYKGKTMM<br>LNDRIQNSNTLQNVLRKAEEYLIMLSPDTPYFEFEHKFQEIGLEKGWG<br>DTAERVLEMVCMLLDLLEAPDSCTLEKFLGRIPMVFNVVILSPHGYFA<br>QENVLGYPDTGGQVVYILDQVPALEREMLKRIKEQGLDIIPRILIVTRLL<br>PDAVGTTCGQRIEKVYGAEHSHILRVPFRTEKGIVRKWISRFEVWPYM<br>ETFIEDVAKEISAELQAKPDLIIGNYSEGNLAASLLAHKLGVTQCTIAH<br>ALEKTKYPDSDIYWKKFDEKYHFSSQFTADLIAMNHTDFIITSTFQEIAG<br>SKDTVGQYESHMAFTMPGLYRVVHGINVFDPKFNIVSPGADINLYFSY<br>SETEKRLTAFHPEIDELLYSDVENDEHLCVLKDRTKPILFTMARLDRVK<br>NLTGLVEWYAKNPRLRGLVNLVVVGGDRRKESKDLEEQAEMKKMYE<br>LIETHNLNGQFRWISSQMNRVRNGELYRYIADTKGAFVQPAFYEAFGL<br>TVVEAMTCGLPTFATNHGGPAEIIVHGKSGFHIDPYHGEQAADLLAD<br>FFEKCKKEPSHWETISTGGLKRIQEKYTWQIYSERLLTLAAVYGFWKH<br>VSKLDRLEIRRYLEMFYALKYRKMAEAVPLAAE	805
Lycopersicon esculentum	LeSS1	NP_001300839.1	MAERVLTRVHRLRERVDATLCAHRNEILLFLSRIESHGKGILKPHELLA<br>EFDAIRQDDKDKLNEHAFEELLKSTQEAIVLPPWVALAIRLRPGVWEY<br>VRVNVNALVVEELSVPEYLQFKEELVDGASNGNFVLELDFEPFTASFP<br>KPTLTKSIGNGVEFLNRHLSAKMFHDKESMAPLLEFLRAHHYKGKTM<br>MLNDRIHNSNTLQNVLRKAEEYLIMLPPETPFFEFEHKFQEIGLEKGW<br>GDTAERVLEMVCMLLDLLEAPDSCTLEKFLGRIPMVFNVVILSPHGYF<br>AQENVLGYPDTGGQVVYILDQVPALEREMLKRIKEQGLDIIPRILIVTRL<br>LPDAVGTTCGQRLEKVYGTEHSHILRVPFGTEKGIVRKWISRFEVWPY<br>METFIEDVAKEISAELQAKPDLIIGNYSEGNLAASLLAHKLGVTQCTIA<br>HALEKTKYPDSDIYWKKFDEKYHFSSQFTADLIAMNHTDFIITSTFQEI<br>AGSKDTVGQYESHMAFTMPGLYRVVHGINVFDPKFNIVSPGADINLYF<br>PYSESEKRLTAFHPEIDELLYSDVENDEHLCVLKDRTKPILFTMARLDR<br>VKNLTGLVEWYAKNPRLRGLVNLVVVGGDRRKESKDLEEQAEMKK	805

Table S2. *Cont.*

			MYELIETHNLNGQFRWISSQMNRVRNGELYRYIADTKGAFVQPAFYE AFGLTVVEAMTCGLPTFATNHGGPAEIIVHGKSGFHIDPYHGEQAAD LLADFFEKCKKEPSHWETISTGGLKRIQEKYTWQIYSERLLTLAAVYGF WKHVSKLDRLEIRRYLEMFYALKYRKMAEAVPLAAE	
	LeSS2	NP_001234804.2	MAERVLTRVHRLRERVDATLAAHRNEILLFLSRIESHGKGILKPHELLA EFDAIRQDDKDKLNEHAFEELLKSTQEAIVLPPWVALAIRLRPGVWEY VRVNVNALVVEELSVPEYLQFKEELVDGASNGNFVLELDFEPFTASFP KPTLTKSIGNGVEFLNRHLSAKMFHDKESMAPLLEFLRAHHYKGKTM MLNDRIHNSNTLQNVLRKAEEYLIMLPPETPFFEFEHKFQEIGLEKGW GDTAERVLEMVCMLLDLLEAPDSCTLEKFLGRIPMVFNVVILSPHGYL AQENVLGYPDTGGQVVYILDQVPALEREMLKRIKEQGLDIIPRILIVTRL LPDAVGTTCGQRLEKVYGTEHSHILRVPFRTEKGIVRKWISRFEVWPY METFIEDVAKEISAELQAKPDLIIGNYSEGNLAASLLAHKLGVTQCTIA HALEKTKYPDSDIYWKKFDEKYHFSSQFTADLIAMNHTDFIITSTFQEI AGSKDTVGQYESHMAFTMPGLYRVVHGINVFDPKFNIVSPGADINLYF PYSESEKRLTAFHPEIDELLYSDVENDDHLCVLKDRTKPILFTMARLDR VKNLTGLVEWYAKNPRLRGLVNLVVVGGDRRKESKDLEEQAEMKK MYELIETHNLNGQFRWISSQMNRVRNGELYRYIADTKGAFVQPAFYE AFGLTVVEAMTCGLPTFATNHGGPAEIIVHGKSGFHIDPYHGEQAAD LLADFFEKCKKEPSHWETISTGGLKRIQEKYTWQIYSERLLTLAAVYGF WKHVSKLDRLEIRRYLEMFYALKYRKMAEAVPLAAE	805
	LeSS3	NP_001300840.1	MAQRVLTRVHSLRERLDATLDAHRNEILLFLSRIESHGKGILKPHQLLA EFESIQKEDKDKLNDHAFEEVLKSTQEAIVLPPWVALAIRLRPGVWEY VRVNVNALSVEELTVPEFLQFKEELVNGTSSDNFVLELDFEPFTASFPK PTLTKSIGNGVEFLNRHLSAKMFHDKESMTPLLEFLRVHHYNGKSMM LNDRIQNLYTLQKVLRKAEEYLTTLSPETSYSSFEHKFQEIGLERGWGD TAERVLEMICMLLDLLEAPDSCTLEKFLSRIPMVFNVVIPSPHGYFAQE NVLGYPDTGGQVVYILDQVPALEREMLKRIKEQGLDIKPRILIVTRLLP DAVGTTCGQRLEKVFGTEHSHILRVPFRTEKGIVRKWISRFEVWPYME TFIEDVGKEITAELQAKPDLIIGNYSEGNLAASLLAHKLGVTQCTIAHA LEKTKYPDSDIYLNKFDEKYHFSAQFTADLIAMNHTDFIITSTFQEIAGS KDTVGQYESHMAFTMPGLYRVVHGIDVFDPKFNIVSPGADVNLYFPY SEKEKRLTTFHPEIEDLLFSDVENEEHLCVLKDRNKPIIFTMARLDRVK NLTGLVEWYAKNPRLRELVNLVVVGGDRRKESKDLEEQAEMKKMYE LIKTHNLNGQFRWISSQMNRVRNGELYRYIADTRGAFVQPAFYEAFGL TVVEAMSCGLPTFATNQGGPAEIIVHGKSGFQIDPYHGEQAADLLAEF FEKCKVDPSHWEAISKGGLKRIQEKYTWQIYSDRLLTLAAVYGFWKH VSKLDRLEIRRYLEMFYALKFRKLAELVPLAVE	805
	LeSS4	NP_001300841.1	MAQRVLTRVHSLRERLDATLDAHRNEILLFLSRIESHGKGILKPHQLLA EFESIQKEDKDKLNDHAFEEVLKSTQEAIVLPPWVALAIRLRPGVWEY VRVNVNALSVEELTVPEFLQFKEELVNGTSSDNFVLELDFEPFTASFPK PTLTKSIGNGVEFLNRHLSAKMFHDKESMTPLLEFLRVHHYNGKSMM LNDRIQNLYTLQKVLRKAEEYLTTLSPETSYSSFEHKFQEIGLERGWGD TAERVLEMICMLLDLLEAPDSCTLEKFLSRIPMVFNVVILSPHGYFAQE NVLGYPDTGGQVVYILDQVPALEREMLKRIKEQGLDIKPRILIVTRLLP DAVGTTCGQRLEKVFGTEHSHILRVPFRTEKGIVRKWISRFEVWPYME TFIEDVGKEITAELQAKPDLIIGNYSEGNLAASLLAHKLGVTQCTIAHA LEKTKYPDSDIYLNKFDEKYHFSAQFTADLIAMNHTDFIITSTFQEIAGS KDTVGQYESHMAFTMPGLYRVVHGIDVFDPKFNIVSPGADVNLYFPY SEKEKRLTTFHPEIEDLLFSDVENEEHLCVLKDRNKPIIFTMARLDRVK NLTGLVEWYAKNPRLRELVNLVVVGGDRRKESKDLEEQAEMKKMYE	805

Table S2. *Cont.*

			LIKTHNLNGQFRWISSQMNRVRNGELYRYIADTRGAFVQPAFYEAFGL TVVEAMSCGLPTFATNQGGPAEIIVHGKSGFQIDPYHGEQAADLLAEF FEKCKVDPSHWEAISKGGLKRIQEKYTWQIYSDRLLTLAAVYGFWKH VSKLDRLEIRRYLEMFYALKFRKLAELVPLAVE	
Pyrus bretschneideri Redh.	PbSS1	Pbr035996.1	MAERVLTRVQSLRERLDETLSTQRNDFLALLSRIESKGKGFLQPHQLLA EFEAIPENNRQKLLDGAFGEEAIVLPPWVALAVRPRPGVWEYIRVNVH ALALEELCVAEYLQLKEELVDRSSNGNFVLELDFQPFNASFPRPTLSKSI GNGVQFLNRHLSAKLFHDKESLHPLLEFLRVHCYEGRNMMLNNRIK NVNELQRVLRKAEDFLSSIVPRTPYKEFEPKLQAIGLERGWGDTAERVL EMIQLLLDLLEAPNPFTLEKFLGQIPMVFNVVILSPHGYFAQDHVLGFP DTGGQVVYILDQVRALESEMLKRIKLQGLDITPRIIILTRLLPDAVGTTC GDRLEKVYGCEHSDILRVPFRDEKGAIVRRWISRFEVWPYLETYAEDAI KELSKEMQGKPDLIIGNYSDGNIVASLMAHKLGVTQCTIAHALEKSKY PDSDLYWKKLDENYHFSCQFTADLIAMNHTDFIITSTFQEIAGSKDTVG QYESHTAFTLPGLYRVVHGIDVFDPKFNIVSPGADMSVYFPYSEKEKRL THFHPEIEELLYSQVENIEQICVLKDRNKPIIFTMARLDRVKNITGLVEW YGKNAKLRELVNLVVAAGDHRNESKDNEEKAELKKMYELIETYKLN GQLRWISSQMNRVRNGELYRYICDTRGAFVQPAVYEAFGLTVIEAMT CGLPTFATCKGGPAEIIVNGKSGYHIDPYQGDQAAEILVDFFEKSKADP SHWDKISQGGLQRIYEKYTWQIYSERLLTLTGVYGFWKDVSNLDRLEN RRYLEMFYALKFRKLAASVPLAVEE	801
	PbSS2	Pbr035997.1	MAERVLTRVQSLRERLDQTLSTQRNEILALLSRIESKGKGFLQPHQLHA EFEAIPENNRQKLLDGAFGEVLKHTQEAVVLPPWVALAVRPRPGVWE YIRVNVHALTLEELRVAEYLQFKEELVDGSSNGNFVLELDFQPFNASFP RPTLSKSIGNGVEFLNRHLSAKLFHDKESLHPLLEFLQVHCYEGRNMM LNNRIKNVNELQHVLRKAEDFLSSIVPRTPYKEFEPKLQAIGLERGWG DTAERVLEMIQLLLDLLEAPDPFTLEKFLGQIPMVFNVVILSPHGYFAQ DHVLGFPDTGGQVVYILDQVRALESEMLKRIKQQGLDITPRIIILTRLLP DAVGTTCGDRLEKVYGCEHSDILRVPFRDEKGAIVRRWISRFEVWPYL ETYAEDAIKELSKEMQGKPDLIIGNYSDGNIVASLMAHKLGVTQCTIA HALEKSKYPDSDLYWKKLDENYHFSCQFTADLIAMNHTDFIITSTFQEI AGTKDTVGQYESHTAFTLPGLYRVVHGIDVFDPKFNIVSPGADMSVYF PYSEKEKRLTHFHPEIEELLYSQVENIEQICVLKDRNKPIIFTMARLDRV KNITGLVEWYGKNAKLRELVNLLVVAGDRRNESKDIEEKAELKKMYE LIDTYKLNGQLRWISSQMNRVRNGELYRYICDTRGAFVQPAVYEAFGL TVIEAMTCGLPTFATCMGGPAEIIVNGKSGYHIDPYHGDQAAEILVDF FEKSKADPSHWDKISQGGLQRIYEKYTWQIYSERLLTLTGVYGFWKDV STLDRRESRRYLEMFYALKYRKLAASVPLAVEE	807
	PbSS3	Pbr003107.1	MAGNDWINSYLEAILDVGPGLVDDAKSSLLLRERGHFSPTRYFVEEVIT GFDETDLHRSWVRASATRSPQERNTRLENLCWRIWNLARQKKQLEGE ESQRKAKRRLERERGRREATADMSEDLSEGEKGDTVSDMSAHGDSHR GRLPRINSVDAMETWISQQKGKKLYIVLIRHELNFNGLSFVVYKYSCSLH GLIRGEQMELGRDSDTGGQVKYVVELARALGTTPGVYRVDLLTRQVSS PDVDWSYGEPTEMLTPINAEVFEEEMGESSGSYIIRIPFGPKDKYIPKEEL WPHIPEFVDGALNHIMQMSKVLGEQIGGGKPVWPVAIHGHYADAGD SAALLSGALNVPMLFTGHSLGRDKLEQLLKQGRLSRDEINKTYKIMRR IEAEELALDASEIVITSTRQEIEEQWRLYNGFDPILERKLRARLRRNVSC YGRFMPRMVVIPPGMEFHHIVPHDGDMDAETEANEDHPTSPDPPIWS EIMRFFTNPRKPMILALARPDPKKNITTLVKAFGECRPLRELANLTLIM GNRDGIDEMSSTSASLLLSVLKLIDKYDLYGQVAYPKHHKQSDVPDIY RLAAKTKGVFINPAFIEPFGLTLIEAAAHGLPIVATKNGGPVDIHQVLD	1072

Table S2. *Cont.*

			NGLLIDPHDQQSIADALLKLVADKQLWARCRQNGLKNIHLFSWPEH CKTYLSRIASCKQRQPQWQRSEDGGDTSESESASDSLRDIHDLSLNLKF SMDGEKSGTSVNDSLDSEGNTADRRSKIESAVLAWSKGISRDTRRAGF SEKADHNNAGKFPVLRRRKHLIVISVDCETVTELNEITKNIFEATGKEK AEGSVGFILSTSLTIIEIRSFLVFGGLSPKDFDAYICNSGSDLYYPSINSEDR PFVVDFYYHSHIEYRWGGEGLRKTLVRWATSVNDKKTGSEAIVSAAD QLSTDYCYAFEVQAPGKLPPVKELRKVLRIQALRCHVIYCQNGTRVNV IPVLASRSQALRYLYLRWGVDLSKVVVLTGECGDTDYEGLLGGLHQSV VLKGVGRNAISQLHNNRNYPLTDVLALDTSNIVQTSEGCGSDDIRASL EKLGVLKN	
	PbSS4	Pbr022714.1	MSILTPQAPPTPILYKYPISFCHVSYFPLSLSTSPPALSEHRDEEEDDDDD RRKKIESWGIALSSLAPYVDRGKGILQPHDLIDQLEHVIGDDEAKQHL KTGAFSKVLKAAQEAIILPPFVALVVRPRPGVWEYVRVNVYELSVEELT VSEYLSFKEELVDGESSDKYVLELDFRPFNATFVE	183
	PbSS5	Pbr022715.1	MLVFFPATCVQISWNGFRASLFHVLEMINLLLDILQAPDPSILETFLGRI PMVFNVIILSPHGYFGQANVLGLPDTGGQAFLIVYILDQVHALENEML ERIRRQGLDFTPRILIVTRLIPDAKGTTCNQRLEKITGTEYTHILRVPFRS EKGILRKWISRFDVLSLFGDLCSGEITAELQGYPDFIIGNYSDGNLVASM LAYKMGVTQFTADLIAMNNADFIVTSTYQEIAGTEVSRKNTVGQYES HSCFTLLRQYRVVHGINVFDPKFNIMSPGAVMTIYFPYSEKQKRLTSLH GSLEELLYDPDQNDVHIGTLSDRSKPIIFSMARLNQVKNMTGLVECYK KCSKLRDLVNLVVIAGYIDAKKSRDREEIAEIEKMHNLMKEYKLNGQF RWISSQTNRVSTGELYHYIADKRGAFAQPAFYEAFGLTVVEAMTCSPP TLATVHGGPPQIIEHGVSGFHIDPYHPEKAAALLADFFQRCKEDPSYW NTISDGGLQRIYEKCTWKIYSGRLMTLAGVYGFWKYVSKLERRETRQY LEMFHILKFRDLAKSVSRAVDDAH	559
	PbSS6	Pbr008035.1	MCWRIWHLARKKKQIAWDDARRLVKRRLDREQGRHDAEDDLSELSE GEKEKGDVSSAEPTVKDILRSKSDMPVWSDDVNKSRHLYIVLISMHGL VRGENMELGRDSDTGGQVKYVIELARALANTKGVYRVDLLTRQITSSE VDSSYGEPNEMLICPPDGSGSCGAYIVRLPCGPCYKYIPKESLWPHIPEF VDGALGHIANMARALGEEVNGGKPTWPYVIHGHYADAGEVAAHLS GALNVPMVLTGHSLSRNKFEQLLKQGRLTKEDINATYKIMRRIEGEEL GLDSAEMVVTSTRQEIEEQWGLYDGFDLKLERKLRVRRRRGVSCLGRY MPRMVVIPPGMDFSYVTAHDSEGDGDLKSLIGSDRGQSKRHLPPIWSE VMRFFTNPHKPTILALSRPDPKKNTLILGNRDDIEEMSNSSSVVLTTVL RLIDKYDLYGQVAYPKHHKQLDVPDIYRLVAKTKGVFINPALVEPFGL TIIEAAAYGLPVVATKNGGPVDILKALNNGLLVDPHDQKAIEEALLKA ITPITEEPLNESLKDVEDLSLRFSVEGDFKHNGELDAATRQRELIEAITR MSSSTSNVGATYGPGRRQSLFVIAIDCYDQNGDGTQVFQEILVNVKKA AGLGYGQGRVGIVLLTGSSLQDIMKAFKGCQVNIEDFDVLVCKSGSEM YYPWRDLAADADYETHIEYRWPGENVRSMVPRLARLEVGAEDDVVE YARSSSSRYLSVRWAIDLLKVVVFVGEEGDTDNEDLLTGLHKTLVPRG SVEYGSEKLIHGKDSLKREDVVPQDSPNIALVESYQAHEISAALEALGI KCF	818
	PbSS7	Pbr028302.1	MASTSSAVKRSENIAETMPDALRESHFHMKKCFASCTQEAAVVPPYV AFAVRPNPGFSEFVKVNADDLAVDGISATQYLKFKEMIFDESWANDE NALEIDFGTIDFSTPRMTLPSSIGNGLNFVLKLISSRLSTHASCSDYAKPL LDYLLPLNYHGENLMINESLDTVEKLQTALIRAEVLVSTLPKTTPFPNF EQRFKVLGFEKGWGDTAERVGETMRLLSEVLQAPDSVKLASLFSRLPN TFNIVIFSPHGYFGQSNVLGLPDTGGQVVYILDQVRALEEELLLRIKQQ GLAVKPQILVVTRLIPDARGTKCNQELEAVIDTKHSHILRVPFRTDKGV	867

Table S2. *Cont.*

			LRQWVSRFDIYPYLETFAQDATAKILRHMECKPDLIIGNYSDGNLVASL IASKLGITQGTIAHALEKTKYEDSDAKWKEFDPKYHFSCQFTADIISMN CADFVITSTFQEIAGGKDRPGQYESHTAFTMPGLYRVVSGIDVFDPKFN IAAPGADQSVYFPXXXXXXXVYFPYSEKQKRFTKFQPVIEELLYTKEEN DEHIGFLADQKKPIIFSMARLDTVKNLTGLVEWFGKNKRLRNLVNLVI VGGFFDPSKSKDREEIAEIKKLHALVQEYQLSGQFRWIAAQTDRYRNG ELYRCIADTKGAFVQPALYEAFGLTVIEAMNCGLPTFATNQGGPAEIIV DSVSGFHIDPNNGDESSNKIADFFEKCKTDGEYWKKMSAAGLQRINE CYTWKIYANKVLNMGSTYGFWRQLRDAQKLAKETYIHMFYNLLFRK LAKNVAVPSDGYEQPAPKAGIAAVDQRTPASKPPQPEAAPTLAIPQLT PRERDEGGELSQPRSHSRARCLWNCCCVILGLLIILYYKIRNMYN	
	PbSS8	Pbr042506.1	MIEGEEAQRVARRRLERERGRREATEDMSEDLSEGEKGDTVGDISAHG GDSTRGRMKRINSTDAMENWAGQQKEKKFYMVLHGLIRGENMELG RDSDTGGQVKYVVELARALGSMPGVYRVDLLTRQVAAPDVDWSYGE PTEMLNPLNTENSKEELGESSGAYIVRIPFGPRDKYAPKELLWPHIPEFV DGALTHIIQMSKALGEQIGGGQPVWPVAIHGHYADAGDSAALLSGAL NVPMVFTGHSLGRDKLEQLLKQGRQSREEINTTYKIMRRIEAEELTLD ASEIVITSTRQEIESQWRLYDGFDPILERKLRARIKRGVSCHGRFMPRMV VIPPGMEFHHIIPPDGDGDGEGERHDDSSISPDPPIWSEIMRFFTNPRKP MILALARADPKKNITTLVKAFGECRPLRELANLTLIMGNRDDIDEMPS TNASVLLSILKLIDRYDLYGHVAYPKHHKQSDVPDIYRLAAKTKGVFI NPAFIEPFGLTLIEAAAHGLPIVATQNGGPVDIHRVLDNGLLVDPHDQ RSIADALLKLVSDKQLWARCRQNGLKNIHLFSWPEHCKTYLTRITSCK PRQPQWQRNDADFDNSEPDSPNDSLRDIQDISLNLKLSLDGDKTEGSA ALDNALEAEDCAAGGKTKEQNAVLTLSKGVCQKAGATEKADNSSG AGKFLAFRKRKYVCVIAVDCDTTSEFTEIIEKVTEAAGKDRDAGPIGFIL STALGISEIHSLLISGGLSPSQFDAFICNSGGELYYPSSSSEDSPSGLPFVV DIDYRSHIEYRWGAEGLRKTLVRWVANFNEKKGRETVTEDVSASTNH CYAYKVKDPELIPPVKELRKLMRIQALRCHVIYSLNGTRLNVIPVLASR SQALRYLYVRWGLNLSTAVVFVGESGDTDYEGLLGGLHKTVILKGVGI GARKLHANRNYPLEHVFPNDSPNMAQSEGCSENDIRASLVKLGILKR	962
	PbSS9	Pbr033571.1	MASGAAIKRSESIADNMPDALRQSRYHMKRCFAKYIEKGRRIIKLHHL LSEMETVIDDKAERTQVLEGVLGYILCSTQEAVVIPPHVVFSIRPNPGY WEFVKVSSEDLSVVGITLRDFLKYKETLYDENWSNDENALEVDFRAID FSTPHLTLSSSIGNGINYVTKFATSKLAGKLENAQPLVDYLLSLNHQGE QLILNETLNTASKLQATLIVTEVYLSAVPKDTPFQNFELRFKEWGFEKG WGDTAERTKETMRILSEVLQAPDPLIMDRFFSRLPTIFNVVIFSPHGYFG QADVLGLPDTGGQVVYILDQVKALEEELLVRIKQQGLTVKPQILVVTR LIPEARGTKCNQELEPINGTKYSNILRVPFRTEKGILRRWDATAKILDL MEGKPDLIIGNYTDGNLVASLMANKLGITQATIAHALEKTKYEDSDIN WKELDPKYHFSCQFLADTISMNATDFVIASTYQEIAGSKDRPGQYESH TAFTLPGLCRVVSGISVFDPKFNIAAPGADQSVYFPYAEKQKRLTSFHP AIEELLFSKEDNNEHIGFLEDRKKPIIFSMARLDIVKNITGLVEWYGKN KRLRNLVNLVVVGGFFDPSKSKDREEIAEIKKMHTLIEKYQLRDQIRWI AAQTDRNRNGELYRCIADTRGAFVQPALYEAFGLTVIEAMNCGLPTF ATNQGGPAEIIVDGISGFHIDPNNGDEASNKIADFFEKSKTDAAYWDR FSKAGLQRIYECYTWKIYANKVLNMGSTYTFWRQLNKEQKQAKQRYI HMFFNLQYRNLVKNVPIPSDEPEQPVVPKPTAKPKTTLRHVQLNSQFT QQELPACKPILTPAWVILIFVAIGIVFLPIGFASLFASEHVVEIVNHYDRD CVPPNYMDNKLAYIQSSKTNKTCIRRVTIPKQMKSPVYIYYQLDHFYQ NHRRYVKSRSDGQLRSKSNEKKTDTCAPERYTANGAIVPCGLVAWSL	1142

Table S2. *Cont.*

			FNDTYKFSVNNKQLGVSKKDITWKSDPKKKFGSDVYPKNFQSGGLIG GAKLNSSIPLSEQEDLMVWMRTSTLPTFRKLYGKIEADLEADDIVTVTI ENNYNTYIFGGNKKLVLSTASWIGGKNCLLGIAYLTVGGLCLFLAISFL LLYLFKPRPLGDTSYLSWNRSAPGGLIY	
	PbSS10	Pbr008457.1	MASGAAIKRSESIADNMPDALRQSRYHMKRCFAKYIEKGRRIIKLHHL LSEMETVIDDKAERTQVLEGVLGYILCSTQEAVVIPPHVVFSIRPNPGY WEFVKVSSEDLSVVGITLRDFLKYKETLYDENWSNDENALEVDFRAID FSTPHLTLSSSIGNGINYVTKFATSKLAGKLENAQPLVDYLLSLNHQGE QLILNETLNTASKLQATLIVTEVYLSAVPKDTPFQNFELRFKEWGFEKG WGDTAERTKETMRILSEVLQAPDPLIMDRFFSRLPTIFNVVIFSPHGYFG QADVLGLPDTGGQVVYILDQVKALEEELLVRIKQQGLTVKPQILVVTR LIPEARGTKCNQELEPINGTKYSNILRVPFRTEKGILRRWDATAKILDL MEGKPDLIIGNYTDGNLVASLMANKLGITQATIAHALEKTKYEDSDIN WKELDPKYHFSCQFLADTISMNATDFVIASTYQEIAGSKDRPGQYESH TAFTLPGLCRVVSGISVFDPKFNIAAPGADQSVYFPYAEKQKRLTSFHP AIEELLFSKEDNNEHIGFLEDRKKPIIFSMARLDIVKNITGLVEWYGKN KRLRNLVNLVVVGGFFDPSKSKDREEIAEIKKMHTLIEKYQLRDQIRWI AAQTDRNRNGELYRCIADTRGAFVQPALYEAFGLTVIEAMNCGLPTF ATNQGGPAEIIVDGISGFHIDPNNGDEASNKIADFFEKSKTDAAYWDR FSKAGLQRIYECYTWKIYANKVLNMGSTYTFWRQLNKEQKQAKQRYI HMFFNLQYRNLVKNVPIPSDEPEQPVVPKPTAKPKTTLRHVQLNSQFT QQELPACKPILTPAWVILIFVAIGIVFLPIGFASLFASEHVVEIVNHYDRD CVPPNYMDNKLAYIQSSKTNKTCIRRVTIPKQMKSPVYIYYQLDHFYQ NHRRYVKSRSDGQLRSKSNEKKTDTCAPERYTANGAIVPCGLVAWSL FNDTYKFSVNNKQLGVSKKDITWKSDPKKKFGSDVYPKNFQSGGLIG GAKLNSSIPLSEQEDLMVWMRTSTLPTFRKLYGKIEADLEADDIVTVTI ENNYNTYIFGGNKKLVLSTASWIGGKNCLLGIAYLTVGGLCLFLAISFL LLYLFKPRPLGDTSYLSWNRSAPGGLIY	1142
	PbSS11	Pbr008464.1	MASGAAIKRSESIADNMPDALRQSRYHMKRCFAKYIEKGRRIIKLHHL LSEMETVIDDKAERTQVLEGVLGYILCSTQEAVVIPPHVVFSIRPNPGY WEFVKVSSEDLSVVGITLRDFLKYKETLYDENWSNDENALEVDFRAID FSTPHLTLSSSIGNGINYVTKFATSKLAGKLENAQPLVDYLLSLNHQGE QLILNETLNTASKLQATLIVTEVYLSAVPKDTPFQNFELRFKEWGFEKG WGDTAERTKETMRILSEVLQAPDPLIMDRFFSRLPTIFNVVIFSPHGYFG QADVLGLPDTGGQVVYILDQVKALEEELLVRIKQQGLTVKPQILVVTR LIPEARGTKCNQELEPINGTKYSNILRVPFRTEKGILRRWDATAKILDL MEGKPDLIIGNYTDGNLVASLMANKLGITQATIAHALEKTKYEDSDIN WKELDPKYHFSCQFLADTISMNATDFVIASTYQEIAGSKDRPGQYESH TAFTLPGLCRVVSGISVFDPKFNIAAPGADQSVYFPYAEKQKRLTSFHP AIEELLFSKEDNNEHIGFLEDRKKPIIFSMARLDIVKNITGLVEWYGKN KRLRNLVNLVVVGGFFDPSKSKDREEIAEIKKMHTLIEKYQLRDQIRWI AAQTDRNRNGELYRCIADTRGAFVQPALYEAFGLTVIEAMNCGLPTF ATNQGGPAEIIVDGISGFHIDPNNGDEASNKIADFFEKSKTDAAYWDR FSKAGLQRIYECYTWKIYANKVLNMGSTYTFWRQLNKEQKQAKQRYI HMFFNLQYRNLVKNVPIPSDEPEQPVVPKPTAKPKTTLRHVILIFVAIGI VFLPIGFASLFASEHVVEIVNHYDRDCVPPNYMDNKLAYIQSSKTNKT CIRRVTIPKQMKSPVYIYYQLDHFYQNHRRYVKSRSDGQLRSKSNEKK TDTCAPERYTANGAIVPCGLVAWSLFNDTYKFSVNNKQLGVSKKDIT WKSDPKKKFGSDVYPKNFQSGGLIGGAKLNSSIPLSEQEDLMVWMRT STLPTFRKLYGKIEADLEADDIVTVTIENNYNTYIFGGNKKLVLSTASWI	1119

Table S2. *Cont.*

			GGKNCLLGIAYLTVGGLCLFLAISFLLLYLFKPRPLGDTSYLSWNRSAP<br>GGLIY	
	PbSS12	Pbr031564.1	MASTSSAVKRSENIAETMPDALRESHFHMKKCFARLIKPQHIMEELEK<br>SIEDRHERSKVLEGLLGYILSCTQEAAVVPPYVAFAVRPNPGFSEFVKV<br>NADDLAVDGISATQYLKFKEMIFDESWANDENALEIDFGTIDFSTPRM<br>TLPSSIGNGLNFVLKLISSRLSTHASCSDYAKPLLDYLLPLNYHGENLMI<br>NESLDTVEKLQTALIRAEVLVSTLPKTTPFPNFEQRFKVLGFEKGWGDT<br>AERVGETMRLLSEVLQAPDSVKLASLFSRLPNTFNIVIFSPHGYFGQSN<br>VLGLPDTGGQVVYILDQVRALEEELLLRIKQQGLAVKPQILVVTRLIPD<br>ARGTKCNQELEAIIDTKHSHILRVPFRTDKGVLRQWVSRFDIYPYLETF<br>AQDATAKILRHMECKPDLIIGNYSDGNLVASLIASKLGITQGTIAHALE<br>KTKYEDSDAKWKEFDPKYHFSCQFTADIISMNCADFVITSTFQEIAGGG<br>FLADQKKPIIFSMARLDTVKNLTGLVEWFGKNKRLRNLVNLVIVGGFF<br>DPSKSKDREEIAEIKKLHALVQEYQLSGQFRWIAAQTDRYRNGELYRCI<br>ADTKGAFVQPALYEAFGLTVIEAMNCGLPTFATNQGGPAEIIVDGVSG<br>FHIDPNNGDESSNKIADFFEKCKTDGEYWKKMSAAGLQRINECYTWK<br>IYANKVLNMGSTYGFWRQLRDAQKLATETYIHMFYNLLFRKLAKNV<br>AVPSDGYKQPAPKAVTAAVDQRTPASKPPQPEAAPTLAIPQLTPRERD<br>EGGELSQPRSPSRARCLWNCCCVILGLLIILYYKIRNMYN	815
	PbSS13	Pbr012029.1	MRNTIQDTLTAHRNELVLLLSRYVARGNGILLSHQMINELENVIMKGE<br>GMQKLKDSSFSKVLQSALEAIVLAPFVAFALRPRPGVWDLKMKLSWV<br>QRYVCDLEPFNAHFPRPTRSSSIGNGVQFLNRHLSSVIFRSKESLESLLD<br>FLRTHKYDGYDASNEIAAELQGVPDLIIGNYSDGNLVAILKNNVGQYE<br>SYTASTLPGPYQIVHGIDVFDPKFNIVSPGADMCIYFPYSDKERRLTALH<br>GSIEELLYGAEQKDEHIGLAIE	265
	PbSS14	Pbr014902.1	MRNTIQETLAAHRNELVSLLSRYVARGNGILQPHQMINELESVIMEDE<br>GMQKLKDSSFSKVLQSAQEAIVLAPFVAFALRPRPGVWEYVRVNEYEL<br>SVDHLSVAEYLRFKEELMDGECNDKYVLELDLEPFNASFPRPTRSSSIG<br>NGVQFLNRHLSSVMFRNKESLEPLLDFLRTHKHDGHAMMLNDRIQSI<br>PRLQSALAKAEEYLSKFPATTPYSEFEFDLQGMGFERGWGDTAQRVSE<br>MVHLLLEILQAPDPSTLENFLGRIPMVFNVVIVSPHGYFGQANVLGLP<br>DTGGQVVYILDQVRALESEMLLRIQNQGLDVIPKILIVTRLIPDAKGTT<br>CNQRLERVSGTEYTHILRVPFRTENGILRKWISRFDVWPYLETFAEDAS<br>NEIAAELQGVPDLIIGNYSDGNLVATLLSYKLGITQCNIAHALEKTKYP<br>DSDIFWKKHEDKYHFSSQFTADLIAMNNADFIITSTYQEIAGSKNNVG<br>QYESHTAFTLPGLYRVVHGIDVFDPKFNIVSPGADMCIYFPCSDKEKRL<br>TALHGSIEELLYGAEQNDEHIGLLSDSSKPIVFSMARLDRVKNLTGLVE<br>CYAKSAKLREMVNLVVVGGYLDVKNSRDREEIAEVEKMHDLIKKYNL<br>SGQFRWIAAQMNRARNGELYRYIADTKGVFVQPAFYEAFGLTVVEA<br>MTCGLPTFATCHGGPAEIIEHGSSGFHVDPYNPDQVAELLIDFFDQCQ<br>KDPGHWEKISQAGLKRIYERYTWKIYSERLLTLAGVYGFWKHVSKLER<br>RETRRYLEMFYILKYRNLVKSIPLAVEEQH	800
	PbSS15	Pbr027868.1	MAGNDWVNSYLEAILDVGPGLDDAKSSLLLRERGRFSPTRYFVEEVIT<br>RYDETDLHRSWVRAAATARSPEERNTRLENMCWRIWNLARKKKQIE<br>GEEAQRVARHRLERERGRREATEDMSEDLSEGEKGDTVGDISAHGGD<br>STRGGRMKRINSTDAVENWASQQKEQKFYMVLHGLIRGENMELGRD<br>SDTGGQVKYVVELARALGSMPGVYRVDLLTRQVAAPDVDWSYGEPT<br>EMLNPLNTENSKEELGESSGAYIVRIPFGPRDKYVPKELLWPHIPEFVD<br>GALTHILQMSKALGEQIGGGQPVWPVAIHGHYADAGDSAALLSGAL<br>NVPMVFTGHSLGRDKLEQLLKQGRQSREEINTTYKIMRRIEAEELTLD<br>ASEIVITSTRQEIDSQWRLYDGFDPILERKLRARIKRGVSCHGRFMPRM	1054

Table S2. *Cont.*

			VVIPPGMEFHHIIPHDGDGDGEGERHDDSSTSPDPPIWSEIMRFFTNPR KPMILALARADPKKNITTLVKAFGECRPLRELANLTLIMGNRDDIDEM SSTNASVLLSILKLIDRYDLYGHVAYPKHHKQSDVPDIYRLAAKTKGVF INPAFIEPFGLTLIEAAAHGLPIVATQNGGPVDIHRVLDNGLLVDPHD QQSIADALLKLVSDKQLWARCRQNGLKNIHLFSWPEHCKTYLTRITSC KPRQPQWQRNEADFDNSQHDSPSDSLRDIQDISLNLKLSLDGDKTEGS AALDNALETEDRAAGGKIKEQNAVLTLSKGVCEKAGSTEKADNSSGA GKFPAFRKRKYVYVIAVDCDTTSEFTEIIEKVTEAAEKKKDAGPIGFILS TALGISEIHTLLVSGGLSPSQFDAFICNSGAELYYPSSSSEDSPSGLPFVVD LDYRSHIEYRWGAEGLRKTLVRWVANFNEKKGSETVTEDVSASTNHC YAYKVKDPALIPPVTELRRLMRIQALRCHVIYSQNGTRLNVIPVLASRS QALRYLYVRWGLNLSTAVVFVGESGDTDYEGLLGGLHKTVILKGVNS GARKLHANRNYPLEHVFPDDSPNMAQSEGCSQNDIRASLVKLGVLKR	
	PbSS16	Pbr034361.1	MAGNDWINSYLEAILDVGPGLVDDAKSSLLLRERGHFSPTRYFVEEVIT GFDETDLHRSWVRAAATRSPQERNTRLENLCWRIWNLARQKKQLEG EEAQRMAKRRLERERGRREATADMSEDLSEGEKGDLVSDISAHGDSH RSKLPRINSVDAMETWISQQKGKKLYIVLIRHELNFSFYSCSLHGLIRGE QMELGRDSDTGGQVKYVVELARALGTTPGVYRVDLLTRQVSSPDVD WSYGEPTEMLTPINTEGFEEEMGESSGSYIIRIPFGPKDKYIPKEELWPHI PEFVDGALNHIMQMSKVLGEQVGGGKPVWPVAIHGHYADAGDSAA LLSGALNVPMLFTGHSLGRDKLEQLLKQGRLSRDEINKTYKIMRRIEAE ELALDASEIVITSTRQEIEEQWRLYDGFDPILERKLRARIRRNVSCYGRS MPRMVIIPPGMEFHHIVPHDSDMDAETEANEDHPTSPDPPIWSEIMRF FTNPRKPMILALARPDPKKNITTLVKAFGECRPLRELANLTLIMGNRD GIDEMSSTSASLLLSVLKLIDKYDLYGQVAYPKHHKQSDVPEIYRLAAK TKGVFINPAFIEPFGLTLIEAAAHGLPIVATKNGGPVDIHQVLDNGLLI DPHDQQSIADALLKLVADKQLWARCRQNGLKNIHLFSWPEHCKTYL SRIASVKQRQPHWQRSEDGGDTSESDSPSDSLRDIQDLSLNLKFSMDGE KSGTSVNDSLDSEGNTADRSCKIESAVLAWSKGISRDTRKAGFSEKAD HNSAGKFPVLRRRKHLIVISVDCETITELNEITKNIFEAMGKEKAEGSV GFILSTSLTIIEIRSFLVSGGLNPNDFDAYICNSGSDLYYPSINSEERPFVV DFYYHSHIEYRWGGEGLRKTLVRWATSINDKKTGSEEIVSAADQLSTD YCYAFKVQTPGKLPSVKELRKVLRIQALRCHVIYCQNGTRVNVIPVLA SRSQALRYLYLRWGVDLSKVVVVAGECGDTDYEGLLGGLHKSVVLKG VASNAISQLHTNRNYPLSDVLALDTPNIVQTSEGCGSDDIRASLEKLGV LKN	1066
	PbSS17	Pbr032090.1	MELGRDSDTGGQVKYVVELARALGTTPGVYRVDLLTRQVSSPDVDWS YGEPTEMLTPINTEGFEEEMGESSGSYIIRIPFGPKDKYIPKEELWPHIPEF VDGALNHIMQMSKVLGEQVGGGKPVWPVAIHGHYADAGDSAALLS GALNVPMLFTGHSLGRDKLEQLLKQGRLSRDEINKTYKIMRRIEAEEL ALDASEIVITSTRQEIEEQWRLYDGFDPILERKLRARIRRNVSCYGRSMP RMVIIPPGMEFHHIVPHDSDMDAETEANEDHPTSPDPPIWSEIMRFFT NPRKPMILALARPDPKKNITTLVKAFGECRPLRELANLTLIMGNRDGI DEMSSTSASLLLSVLKLIDKYDLYGQVAYPKHHKQSDVPEIYRLAAKT KGVFINPAFIEPFGLTLIEAAAHGLPIVATKNGGPVDIHQVLDNGLLID PHDQQSIADALLKLVADKQLWARCRQNGLKNIHLFSWPEHCKTYLS RIASVKQRQPHWQRSEDGGDTSESDSPSDSLRDIQDLSLNLKFSMDGE KSGTSVNDSLDSEGNTADRSCKIESAVLAWSKGISRDTRKAGFSEKAD HNSAGKFPVLRRRKHLIVISVDCETITELNEITKNIFEAMGKEKAEGSV GFILSTSLTIIEIRSFLVSGGLNPNDFDAYICNSGSDLYYPSINSEERPFVV DFYYHSHIEYRWGGEGLRKTLVRWATSINDKKTGSEEIVSAADQLSTD	873

Table S2. *Cont.*

			YCYAFKVQTPGKLPSVKELRKVLRIQALRCHVIYCQNGTRVNVIPVLA<br>SRSQALRYLYLRWGVDLSKVVVVAGECGDTDYEGLLGGLHKSVVLKG<br>VASNAISQLHTNRNYPLSDVLALDTPNIVQTSEGCGSDDIRASLEKLGV<br>LKN	
	PbSS18	Pbr037395.1	MAERVLTRVQSLRERLDETLSAHRNEILALLSRIEKKGKGFLQPHQLLA<br>EFEEIPETNRRKLLDGAFGEVLKNTQEVIVLPPWVALAVRPRPGVWEYI<br>RVNVHALVFEELRVAEYLQFKEELVDGSANGNFVLELDFEPFNASFPR<br>PTLSKSIGNGVEFLNRHLSAKLFHDKESLHPLLKFLRVHCYEGRNLML<br>NNKINDVNELQHVLRKSEDFLSAIAPETPYKEFEPKLQAIGLERGWGD<br>TAGRVLEMMQLLLDLLEAPDPCTLEKFLDQIPMVFNVVILSPHGYFAQ<br>DNVLGYPDTGGQVVYILDQVRALEAEMLKRVKQQGLDIIPRIIILTRLL<br>PDAVGTTCGDRLEKVYGTEHSDILRVPFRDEKGAIVRRWISRFEVWPY<br>LETYAEDAIKELSKEMHGKPDLIIGNYSDGNIVASLMALKLGVTQCTIA<br>HALEKSKYPDSDLYWKKLDDSYHFSCQFTADLIAMNHTDFIITSTYQEI<br>AGSKETVGQYESHTAFTLPGLYRVVHGIDVFDPKFNIVSPGADMSVYF<br>PYTEKEKRLTNFHPEIEELLYSQVENKEHLCVLKDRNKPIIFTMARLDR<br>VKNITGLVEWYGKNAKLRELVNLVVVAGDRRKESKDNEEKDEMKK<br>MYELIDTYKLNGQFRWISSQMNRVRNGELYRYICDTKGAFVQPAVYE<br>AFGLTVVEAMTCGLPTFATCKGGPAEIIVHGKSGYHIDPYHGDQAAEI<br>LVDFFEKSKADPSHWDKISQAGLQRIYEKYTWQIYSQRLLTLTGVYGF<br>WKHVSKLDRLESRRYLEMFYALKYSKLESWRAGSPAAIWRCFTPSSTA<br>SWRRLFLLLWRSERERFPK	837
	PbSS19	Pbr012642.1	MASAEAIKRSDSIADTMPDALRQSRYHMKRCFAKYIEQGRRIMKLHH<br>LMSEMEAVIDDKDERTQVLGGVLGYILCSTQEAVVVPPHVVFSIRLDP<br>GYWEFVKVSSEDLAVEAITVREFLKYKEALCDEKWSNDEHVLEVDFR<br>AIDFSTPHLTLSSSIGNGIDYVTKFTTSKLAGKLDNAQPLVDYLVSLHH<br>HGEQLILNETLNTASKLQAALIVTEVYLSALPKDTPFQNFELRFKEWGF<br>EKGWGDTAERTQETMKILSEVLQAPDPLNMDRFLSRLPTIFNVVILSPH<br>GYFGQADVLGLPDTGGQVVYILDQVKAMEEELTLRIKQQGLTVKPQIL<br>VVTRLIPEARGTKCNQELEPINGTKYSNILRVPFRTEKGILRRWDATAKI<br>LYLMEGKPDLIIGNYTDGNLVASLMANKLGITQATIAHALEKTKYEDS<br>DVNWKELDPKYHFSCQFLADTISMNATDFVIASTYQEIAGSKDRPGQY<br>ESHTAFTLPGLCRVVSGINVFDPKFNIAAPGADQSVYFPYSEKQKRLTS<br>FYPAIEELLFSKEDNSEHLGFLVDRKKPIIFSMARLDIVKNITGLVEWYG<br>KNKRLRNLVNLVVVGGFFDPSKSKDREEIAEIKKMHTLIEKYQLRGQIR<br>WIAAQADRNRNGELYRCIADTRGAFVQPALYEAFGLTVIEAMNCGLP<br>TFATNQGGPAEIIVDEVSGFHIDPNNGDEASNKIADFFEKSKTDAAYW<br>DRFSNAGLQRIYECYTWKIYANKVLNMGSTYTFWRQLNKEQKQAKQ<br>RYIQMFFNLQYRNLVKNVPVPSDEPEQPAAPKPTAKPQTTLRHLKALT<br>VSIAKVKSKIATNGLFSIFSLKPA	844
	PbSS20	Pbr012641.1	MASAAAVKRSDSIADTMPDALRQSRYHMKRCFAKYIEKGRRIMKLHH<br>LMSEMETVIDDKAEMTQVLEGVLGYILCSTQEAVVVPPHVVFSIRLNP<br>GYWEFVKVSSEDLSVEAITVRDFLKYKEALYDDKWSNDEHGLEVDFR<br>AIDFSTPHLTLSSSIGNGIHYVTKFMTSKLAGKLENAQPLVDYLLSLNH<br>QGEQLILNETLNTALKLQAALIVTEVYLSTLPKDTPFQNFELRFKEWGF<br>EKGWGDTAERTRETMKILSEVLQAPDPLNMDRFFSRLPTIFNVVIFSPH<br>GYFGQADVLGLPDTGGQVVYILDQVKAMEEELTLRIKQQGLTVKPQIL<br>VVTRLIPEARGTKCNQELEQINGTKYSNILRDATAKILYLMEGKPDLIIG<br>NYTDGNLVASLMANKLGITQATIAHALEKTKYEDSDINWKELDPKYH<br>FSCQFLADTISMNATDFVIASTYQEIAGSKDRPGQYESHTAFTLPGLCR<br>VVSGINVFDPKFNIAAPGADQSVYFPYSEKQKRLTSFSPAIEELLFSKED	1138

Table S2. *Cont.*

			NSEHLGFLVDRKKPIIFSMARLDIVKNITGLVEWYGKNKRLRNLVNLV<br>VVGGFFDPSKSKDREEIAEIKKMHTLIEKYQLRGQIRWIAAQTDRNRN<br>GELYRCIADTRGAFVQPALYEAFGLTVIEAMNCGLPTFATNQGGPAEII<br>VDEVSGFHIDPNNGDEASNKIADFFEKSKTDAAYWDSFSNAGLQRIYE<br>CYTWKIYANKVLNMGSTYTFWRQLNKEQKQAKQRYIQMFFNLQYRN<br>LVKNVPVPSDEPEQPAAPKSIAKPQTTSRHLKALTVSIAKVENSKFTQQ<br>ELPACKPILTPASVILIFVAIGIVFIPIGLASLFASERVVEIVHHYDKDCIPL<br>KYADNMLAYIQSSKTNKTCVRKLTIPKQMKSPVYIYYQLDHFYQNHR<br>RYVKSRSDKQLRSKSYENTTDDCAPERYTAKGVIVPCGLVAWSLFNDT<br>YKFSVNNKQLGVSKKDITWKSDQKNKFGSDVYPKNFQSGGLIGGAKL<br>NSSIPLSEQEDLMVWMRTAALPTFRKLYGRIEVDLEADAVITVTIENNY<br>NTYSFRGNKKLVLSTASWIGGKNYLLGVAYLTVGGLCLFLALAFLLLY<br>LIKPRPLGDISYLSWNRSASGGHIY	
	PbSS21	Pbr003395.1	MAERVLTRVQSLRERLDETLSTQRNEILALLSRIESKGKGFLQPHQLHA<br>EFEAIPENNRQKLLDGAFGEVLKHTQEAVVLPPWVALAVRPRPGVWE<br>YIRVNVHALTLEELRVAEYLQFKEELVDGSSNGNFVLELDFQPFNASFP<br>RPTLSKSIGNGVEFLNRHLSAKLFHDKESLHPLLEFLQVHCYEGRNMM<br>LNNRIKNVNELQHVLRKAEDFLTSIAPRTPYKEFEPKLQAMGLERGW<br>GDTAERVLEMIQLLLDLLEAPDPFTLEKFLGQIPMVFNVVILSPHGYFA<br>QDHVLGFPDTGGQVVYILDQVRALESEMLKRIKQQGLDITPRIIILTRLL<br>PDAVGTTCGDRLEKVYGCEHSDILRVPFRDEKGAIVRRWISRFEVWPY<br>LETYAEDAIKELSKEMQGKPDLIIGNYSDGNIVASLMAHKLGVTQCTI<br>AHALEKSKYPDSDLYWKKLDENYHFSCQFTADLIAMNHTDFIVTSTFQ<br>EIAGTKDTVGQYESHTAFTLPGLYRVVHGIDVFDPKFNIVSPGADMDV<br>YFPYSEKEKRLTHFHPEIEELLYGQVENIEQLCVLKDRNKPIIFTMARLD<br>RVKNITGLVEWYGKNAKLRELVNLLVVAGDRRNESKDIEEKAELKKM<br>YELIDTYKLNGQLRWISSQMNRVRNGELYRYICDTRGAFVQPAVYEAF<br>GLTVIEAMTCGLPTFATCMGGPAEIIVNGKSGYHIDPYHGDQAAEILV<br>DFFEKSKADPSHWDKISQGGLQRIYEKYTWQIYSERLLTLTGVYGFWK<br>DVSNLDRLESRRYLEMFYALKYRKLAASVPLAVEE	807
	PbSS22	Pbr026341.1	MSILTPQAPPTPILYKYPISFCHVSYFPLSLSTSPPALSEHRDEEEDDDDD<br>RRKKIESWGIALSSLAPYVDRGKGILQPHDLIDQLEHVIGDDEAKQHL<br>KTGAFSKVLKAAQEAIILPPFVALVVRPRPGVWEYVRVNVYELSVEELT<br>VSEYLSFKEELVDGESSDKYVLELDFRPFNATFVE	183
	PbSS23	Pbr026338.1	MSILTPQAPPTPILYKYPISFCHVSYFPLSLSTSPPALSEHRDEEEDDDDD<br>RRKKIESWGIALSSLAPYVDRGKGILQPHDLIDQLEHVIGDDEAKQHL<br>KTGAFSKVLKAAQEAIILPPFVALVVRPRPGVWEYVRVNVYELSVEELT<br>VSEYLSFKEELVDGESSDKYVLELDFRPFNATFVE	183
	PbSS24	Pbr026337.1	MFRNKESLKPLLDFLRAHKYKEQPLMLNERIQSESKLQSALAKAEDHL<br>SKLQLETPYSEFEYM	63
	PbSS25	Pbr003394.1	MAERVLTRVQSLRERLDETLSTQRNDFLALLSRIESKGKGFLQPHQLLA<br>EFEAIPENNRQKLLDGAFGEEAIVLPPWVALAVRPRPGVWEYIRVNVH<br>ALALEELCVAEYLQLKEELVDRSSNGNFVLELDFQPFNASFPRPTLSKSI<br>GNGVQFLNRHLSAKLFHDKESLHPLLEFLRVHCYEGRNMMLNNRIK<br>NVNELQRVLRKAEDFLSSIVPRTPYKEFEPKLQAIGLERGWGDTAERVL<br>EMIQLLLDLLEAPNPFTLEKFLGQIPMVFNVVILSPHGYFAQDHVLGFP<br>DTGGQVVYILDQVRALESEMLKRIKQQGLDITPRIIILTRLLPDAVGTTC<br>GDRLEKVYGCEHSDILRVPFRDEKGAIVRRWISRFEVWPYLETYAEDAI<br>KELSKEMQGKPDLIIGNYSDGNIVASLMAHKLGVTQCTIAHALEKSKY<br>PDSDLYWKKLDENYHFSCQFTADLIAMNHTDFIITSTFQEIAGTKDTV<br>GQYESHTAFTLPGLYRVVHGIDVFDPKFNIVSPGADMSVYFPYSEKEKR	790

Table S2. *Cont.*

			LTHFHPEIEELLYSQVENIEQICVLKDRNKPIIFTMARLDRVKNITGLVE WYGKNAKLRELVNLVVAAGDRRNESKDNEEKAELKKMYELIETYKL NGQLRWISSQMNRVRNGYDLWTAHICHLQGCVIEAMTCGLPTFATC KGGPAEIIVNGKSGYHIDPYQGDQAAEILVDFFEKSKADPSHWEKISQ GGLQRIYEKYTWQIYSERLLTLTGVYGFWKDVSNLDRLENRRYLEMFY ALKFRKLAASVPLAVEE	
	PbSS26	Pbr026342.1	MLVFFPATCVQISWNGFRASLFHVLEMINLLLDILQAPDPSILETFLGRI PMVFNVIILSPHGYFGQANVLGLPDTGGQAFLIVYILDQVHALENEML ERIRRQGLDFTPRILIVTRLIPDAKGTTCNQRLEKITGTEYTHILRVPFRS EKGILRKWISRFDVLSLFGDLCSGEITAELQGYPDFIIGNYSDGNLVASM LAYKMGVTQFTADLIAMNNADFIVTSTYQGIAGTEVSRYIGTSYSKNT VGQYESHSCFTLLRQYRVVHGINVFDPKFNIMSPGAVMTIYFPYSEKQ KRLTSLHGSLEELLYDPDQNDVHIGTLSDRSKPIIFSMARLNQVKNMT GLVECYKKCSKLRDLVNLVVIAGYIDAKKSRDREEIAEIEKMHNLMKE YKLNGQFRWISSQTNRVSTGELYHYIADKRGAFAQPAFYEAFGLTVVE AMTCSPPTLATVHGGPPQIIEHGVSGFHIDPYHPEKAAALLADFFQRC KEDPSYWNTISDGGLQRIYEKCTWKIYSGRLMTLAGVYGFWKYVSKLE RRETRQYLEMFHILKFRDLAKSVSRAVDDAH	566
	PbSS27	Pbr026336.1	MLVFFPATCVQISWNGFRASLFHVLEMINLLLDILQAPDPSILETFLGRI PMVFNVIILSPHGYFGQANVLGLPDTGGQAFLIVYILDQVHALENEML ERIRRQGLDFTPRILIVTRLIPDAKGTTCNQRLEKITGTEYTHILRVPFRS EKGILRKWISRFDVLSLFGDLCSGEITAELQGYPDFIIGNYSDGNLVASM LAYKMGVTQFTADLIAMNNADFIVTSTYQGIAGTEVSRYIGTSYSKNT VGQYESHSCFTLLRQYRVVHGINVFDPKFNIMSPGAVMTIYFPYSEKQ KRLTSLHGSLEELLYDPDQNDVHIGTLSDRSKPIIFSMARLNQVKNMT GLVECYKKCSKLRDLVNLVVIAGYIDAKKSRDREEIAEIEKMHNLMKE YKLNGQFRWISSQTNRVSTGELYHYIADKRGAFAQPAFYEAFGLTVVE AMTCSPPTLATVHGGPPQIIEHGVSGFHIDPYHPEKAAALLADFFQRC KEDPSYWNTISDGGLQRIYEKCTWKIYSGRLMTLAGVYGFWKYVSKLE RRETRQYLEMFHILKFRDLAKSVSRAVDDAH	566
	PbSS28	Pbr009578.1	MAGNDWVNSYLEAILDVGPGLDDAKSSLLLRERGRFSPTRYFVEEVIT RYDETDLHRSWVRAAATARSPEERNTRLENMCWRIWNLARKKKQIE GEEAQRVARHRLERERGRREATEDMSEDLSEGEKGDTVGDISAHGGD STRGGRMKRINSTDAVENWTSQQKEQKFYMVLHGLIRGENMELGCD SDTGGQVKYVVELARALGSMPGVYRVDLLTRQVAAPDVDWSYGEPT EMLNPLNTENSKEELGESSGAYIVRIPFGPRDKYVPKELLWPHIPEFVD GALTHILQMSKALGEQIGGGQPVWPVAIHGHYADAGDSAALLSGAL NVPMVFTGHSLGRDKLEQLLKQGRQSREEINTTYKIMRRIEAEELTLD ASEIVITSTRQEIDSQWRLYDGFDPILERKLRARIKRGVSCHGRFMPRM VVIPPGMEFHHIIPHDGDGDGEGERHDDSSTSPDPPIWSEIMRFFTNPR KPMILALARADPKKNITTLVKAFGECRPLRELANLTLIMGNRDDIDEM SSTNASVLLSILKLIDRYDLYGHVAYPKHHKQSDVPDIYRLAAKTKGVF INPAFIEPFGLTLIEAAAHGLPIVVTQNGGPVDIHRVLDNGLLVDPHD QQSIADALLKLVSDKQLWARCRQNGLKNIHLFSWPEHCKTYLTRITSC KPRQPQWQRNEADFDNSQHDSPSDSLRDIQDISLNLKLSLDGDKTEGS AALDNALETEDRAAGGKIKEQNAVLTLSKGVCEKAGSTEKADNSSGA GKFPAFRKRKYVYVIAVDCDTTSEFTEIIEKVTEAAEKNKDAGPIGFIVS TALGISEIHTLLVSGGLSPSQFDAFICNSGAELYYPSSSEDSPSGLPFVVD LDYRSHIEYRWGAEGLRKTLVRWVANFNEKKGSETVTEDVSASTNHC YAYKVKDPALIPPVTELRRLMRIQALRCHVIYSQNGTRLNVIPVLASRS	1054

Table S2. *Cont.*

			QALRYLYVRWGLNLSTAVVFVGESGDTDYEGLLGGLHKTVILKGVNS<br>GARKLHANRNYPLEHVFPDDSPNMAQSEGCSQNDIRASLVKLGVLKR	
	PbSS29	Pbr001616.1	MASTSSAVKRSENIAETMPDALRESHFHMKKCFASCTQEAAVVPPYV<br>AFAVRPNPGFSEFVKVNADDLAVDGISATQYLKFKEMIFDESWANDE<br>NALEIDFGTIDFSTPRMTLPSSIGNGLNFVLKLISSRLSTHASCSDYAKPL<br>LDYLLPLNYHGENLMINESLDTVEKLQTALIRAEVLVSTLPKTTPFPNF<br>EQRFKVLGFEKGWGDTAERVGETMRLLSEVLQAPDSVKLASLFSRLPN<br>TFNIVIFSPHGYFGQSNVLGLPDTGGQVVYILDQVRALEEELLLRIKQQ<br>GLAVKPQILVVTRLIPDARGTKCNQELEAIIDTKHSHILRVPFRTDKGV<br>LRQWVSRFDIYPYLETFAQDATAKILRHMECKPDLIIGNYSDGNLVASL<br>IASKLGITQGTIAHALEKTKYEDSDAKWKEFDPKYHFSCQFTADIISMN<br>CADFVITSTFQEIAGGKDRPGQYESHTAFTMPGLYRVVSGIDVFDPKFN<br>IAAPGADQSVYFPSSEKQKRFTKFQPVIEELLYTKEENDEHIGFLADQK<br>KPIIFSMARLDTVKNLTGLVEWFGKNKRLRNLVNLVIVGGFFDPSKSK<br>DREEIAEIKKLHALVQEYQLSGQFRWIAAQTDRYRNGELYRCIADTKG<br>AFVQPALYEAFGLTVIEAMNCGLPTFATNQGGPAEIIVDGVSGFHIDP<br>NNGDESSNKIADFFEKCKTDGEYWKKMSAAGLQRINECYTWKIYAN<br>KVLNMGSTYGFWRQLRDAQKLATETYIHMFYNLLFRKLAKNVAVPS<br>DGYKQPAPKAVTAAVDQRTPASKPPQPEAAPTLAIPQLTPRERDEGGE<br>LSQPRSPSRARCLWNCCCVILGLLIILYYKIRNMYN	856
	PbSS30	Pbr001745.1	MGMVVQVIATRNTRERSNRLENMCWRIWHLARKKKQIAWDDARRL<br>VKRRLDREQGRHDAEDDLSELSEGEKEKGDVSSAEPTVKDVLRSKSD<br>MPVWSDDVNKSRHLYIVLISMHGLVRGENMELGRDSDTGGQVKYVIE<br>LARALANTKGVYRVDLLTRQITSSEVDSSYGEPNEMLICPPDGSGSCGA<br>YIVRLPCGPCDKYIPKESFWPHTPEFVDGALSHIANMARALGEEVNGG<br>KPTWPYVIHGHYADGGEVAAHLSGALNVPMVLTGHSLGRNKFEQLL<br>KQGRLTKEDINATYKIMRRIEGEELGLDSAEMVVTSTRQEIEEQWGLY<br>DGFDLKLERKLRVRRRRGVSCLGRYMPRMVVIPPGMDFSYVTAHDSE<br>GDGDLISLIGSDRGQSKRHLPPIWSETLILGNRDDIEEMSNGSSVVLTTV<br>LRLIDKYDLYGQVAYPKHHKQLDVPDIYRLAAKTKGVFINPALVEPFG<br>LTIIEAAAYGLPVVATKNGGPVDILKLPHYFHKQR	510
Prunus persica	PPSUS1	ppa001535m	MAERALTRVQSLRERLDVTLSAHRNEIVALLSRIVNKGKGFMQPHELV<br>AEFEAIPESNRQKLLDGAFGEVLTHTQEVIVLPPWVALAVRPRPGVWE<br>YIRVNVDALVVEELQVPEYLHFKEELVDGSANGNFVLELDFEPFNASF<br>PRPTLSKSIGNGVEFLNRHLSAKLFHDKESMHPLLEFLRVHCYKGKNM<br>MLNDRIHNVNELQHVLRKAEDYLSTIAPETPYKQFEDKLQKLGLERG<br>WGDTAERVLEMIQLLLDLLEAPDPCTLEKFLGQIPMVFNVVILSPHGY<br>FAQDNVLGYPDTGGQVVYILDQVRALETEMLKRIKQQGLDITPRIIILT<br>RLLPDAVGTTCGERLEKVYNTEYSHILRVPFRTEKGIVRRWISRFEVWP<br>YLETYAEDAIQELSKELHGKPDLIIGNYSDGNIVASLMAHKLGVTQCTI<br>AHALEKTKYPDSDLYWKKLDDKYHFSSQFTADLIAMNHTDFIITSTFQ<br>EIAGSKDTVGQYESHTAFTLPGLYRVVHGIDVFDPKFNIVSPGADMSIY<br>FPYSEKEKRLTSFHPEIEELLYSQVENKEHLCVLKDRNKPIIFTMARLDR<br>VKNITGLVEWYGKNAKLRELVNLVVVAGDRRKESKDNEEKAEMKK<br>MYELIDTYKLNGQFRWISSQMNRVRNGELYRVIADTKGAFVQPAVYE<br>AFGLTVVEAMTCGLPTFATCKGGPAEIIVHGKSGYHIDPYHGDQAADI<br>LVDFFEKSRADPSHWDKISQGGLQRIYEKYTWQIYSERLLTLTGVYGF<br>WKHVSNLDRLESRRYLEMFYALKYRKLAESVPRAEEE	806
	PPSUS2	ppa001573m	MRKSIEDTLAAHRNELVSLLSRYVDRGNGILQPHQMINELENVIAEDE<br>GMQKLKDSPFSIVLQSAQEAIVQTPFVALALRPRPGVWEYVRVNVYEL<br>SVDLLSVAEYLWFKEELLDGECNDKYVLELDLEPFNATFPRPTRSSSIG	800

Table S2. *Cont.*

			NGVQFLNRHLSSIMFRNKESLEPLLDFLRTHKHDGHAMMLNDRIQSIP RLQSALAKAEEYLSKLLATTPYSDFEFDLQGMGFERGWGDTAQRVSE MVHLLLEILQAPDPSTLETFLGRIPMVFNVVIVSPHGYFGQANVLGLPD TGGQVVYILDQVRALENEMLLRIQNQGLDVIPKILIVTRLIPDAKGTTC NQRLERISGTEHTHILRVPFRTENGIMRKWISRFDVWPYLETFAEDASN EIAAELQGVPDLIIGNYSDGNLVATLLSNKLGISQCNIAHALEKTKYPD SDIYWKKHEDKYHFSSQFTADLIAMNNADFIITSTYQEIAGSKNNVGQ YESHTAFTLPGLYRVVHGIDVFDPKFNIVSPGADMCIYFPYSEKERRLT ALHGSIEELLYGAEQNEEHIGILSDRSKPIVFSMARLDRVKNLTGLVEC YGKSTKLRELVNLVVVGGYIDAKNSRDREEVAEIEKMHDLVKKYNLS GQFRWIAAQMNRARNGELYRYIADTKGVFVQPAFYEAFGLTVVEAM TCGLPTFATCHGGPAEIIEHGISGFHIDPYHQDQVAALLIDFFDQCQKH PGYWEKISEAGLKRIYERYTWKIYSERLLNLAGVYGFWKHVSKLERRET RRYLDMFYILKYRNLVKSIPLAVDEQH	
	PPSUS3	ppa002723m	MFRNKDSLEPLLDFLRAHKYKGHALMLNDRIQSIPKLQSVLGKAEDY LSKLPPETPSSEFEYVLQGMGFERGWGDNAVHVLEMMHLLLDILQAP DPSILETFLGRVPMVFNVVILSPHGYFGQANVLGLPDTGGQIVYILDQV RALENEMLHRIQRQGLDIAPRILIVTRLIPDSKGTTCNQRLERVSGTEHT HILRVPFRSEKGVLRKWISRFDVWPYLETFAEDAASELTAELQGYPDFII GNYSDGNLVASLLAYKMGVTQCTIAHALEKTKYPDSDIYWKKFEDKY HFSSQFTADLIAMNNADFIITSTYQEIAGTKNTVGQYESHSSFTLPGQY RVVHGIDVFDPKFNIVSPGADMAIYFPYSEKTKRLTSLHGSIEELLYDPV QNDVHIGTLDDRSKPLLFSMARLDRVKNISGLVECYAKNPKLRDLVN LVVVAGYIDVKKSKDREEIEEIEKMHGLLKEYKLNGQFRWISAQNNRA RNGELYRYIADTRGAFVQPALYEAFGLTVVEAMTCGLPTFATCHGGP AEIIEHGKSGFHIDPYHPEQAGALMADFFERCKEDPSYWSTFSDAGLK RIYERYTWKIYSERLMTLAGVYGFWKYVSKLERRETRRYLEMFYILKFR DLASSVPSASDE	604
	PPSUS4	ppa001845m	MGFSTYILVSLIFRCFMQEVCAGEEAIVLPPFVALAFRPRPGVWEYVRV NVYELSVDHLTVAEYLQFKEELIDGECNDNYVLELDFEPFNAAFPRPT RSSSIGNGVQYLNRHLSSIMFSKKESLEPLLDFLRTHKHDGHAMMLND RIHSILRLQSALAKAEEYLSKFPPTTPYSEFQFDLQGMGFERGWGDTAQ RVSEMVHLLLEILQAPDPSTLESFLGRIPMVFNVVIVSPHGYFGQANVL GLPDTGGQVVYILDQVRALENEMLLRIQNQGLGVIPKILIVTRLIPDAK GTTCNQRLERVSSTEHTHILRVPFRTKNGILRKWLSRFDVWPYLETFAE DASNEIAAVFQGVPDLIIGNYSDGNLVATLLSYKLGITQCNIAHALEKT KHPDFDIYWKKHEDKYHFSSQFTADLIAMNNADFIITSTYQEIAGSKN NVGQYESHTAFTLPGLYRVVHGIDVFDPKFNIVSPGADMCIYFPYSEKE RRLTALHGSIEELLYGAEQNEEHIGILSDRSKRIVFSMARLDRVKNLTG LVEFYAKSTKLRELVNLVVVGGYIDVKNCRDREEMAEIEKMHGLIKKY NLSGQFRWIVTQMNHARNGELYRYVADTKGVFVQPAFYEAFGLTVV EAMSCGLPTFATCHGGPAEIIEHGISGFHIDPYHPDQVAALLVDFFDQ CQKDPGYWERISETGLKRIFERYTWKIYSERLLNLAGVYGFWKHVSKL ERRETRRYLEMFYILTYRNLVSSVILPSMTQ	757
	PPSUS5	ppa017606m	MASGAAIKRSESIAESMPEALRQSRYHMKRCFAKYIEKGKRIMKLPHL MSEMETVIDDKVERNQVLEGVLGYILCSTQEAVVIPPFVVFAIRPNPGY WEFVKVSSEDLSVESITVRDYLKFKETLYDEKWSNDENTLEVDFRAIDF STPHLTLSSSIGNGLNFVSKFTSSKLAGRLENAQPLVDYLLSLNHEGEN LILNENLNTASKLQTALIVTEVYLSALPKDMPYQNFELRFKEWGFEKG WGDTAERTKETMKLLSEVLQAPDPLNLERFFSRLPIIFNVVIFSPHGYFG QADVLGLPDTGGQVVYILDQVQALEEELLLRIKQQGLTVKPQILVVTR	833

Table S2. *Cont.*

			LIPEAKGTKCNQELEPINGTKYSNILRVPFRTEKGILRRWVSRFDIYPYL ELFAQDASAKVLDIMEGKPDLIIGNYSDGNLVASLMASKLGITQATIA HALEKTKYEDSDIKWKELDPKYHFSCQFLADTISMNATDFVIASTYQEI AGSKDRPGQYESHTAFTLPGLCRVVSGINVFDPKFNIAAPGADQSVYF PYTEKQKRLTSFHPAIEELLYSKEDNSEHIGFLADRKKPIIFSMARLDTV KNITGLVEWYGKNKRLRNLVNLAVVGGFFDPSKSKDREEIAEIKKMH TLIEKYQLRGQIRWIAAQTDRNRNGELYRCIADTRGAFVQPALYEAFG LTVIEAMNCGLPTFATNQGGPAEIIVDGISGFHIDPNNGDEASNKIADF FEKSKTDATYWDRFSKAGLQRIYECYTWKIYANKVLNMGSTYTFWRQ LNKEQKQAKQRYIQMFFNLQYRNLVKNVPIPSDEAEQPVPKPTAKSQ PTPRHVNLN	
	PPSUS6	ppa001135m	MASTPALKRSDTIAETMPDALRQSRYHMKKCFARFVAMGKRLMKMQ HVMEELEKSIEDKHERSKVLEGLLGYIISSTQEAAVVPPYVAFAVRPNP GFWEFVKVNADDLAVDGITASEYLKFKEMIFDDNWANDENALEIDFG GIDFATPRMKLPSSIGNGLNFILKLISSRLATANSSDYAKPLLDYLSQLH YHGENLMINETLNTVAKLQTALIQAEVVVSTLPKDTPFPNFEHRLKEL GFEKGWGDTAERVGETMKMLSEVLQAPDSAKLECFFSRLPNTFNIVIF SPHGYFGQSDVLGLPDTGGQVVYILDQVRALEEELLLRIKQQGLAVKP QILVVTRLIPDARGTKCNQELEAIINTKHSHILRVPFRTHRGILRQWLSR FDIYPYLETFAQDATAKILERMECKPDLIIGNYSDGNLVASLTAGKLGIT QGTIAHALEKTKYEDSDAKWKEFDPKYHFSCQFTADIISMNSADFVITS TFQEIAGGKDRPGQYESHTAFTMPGLYRVVSGIDVFDPKFNIAAPGAD QSVYFPCSEKQRRFTKFHPAIEELLYNKAENDEHIGYLADKKKPIIFSM ARLDTVKNLSGLVEWFGKNKRLRSLVNLVIVGGFFDPSKSKDREEIAEI KKVHALVQEYRLTGQFRWIAAQTDRYRNGELYRCIADTRGAFVQPAL YEAFGLTVIEAMNCGLPTFATNQGGPAEIIVDGVSGFHIDPNNGDESS NKIADFFEKCKTDGEYWNKMSAAGLQRIYECYTWKIYANKVLNMGS TYGFWRQLRKEQKLANQTYIHMFYSLLFRNLARNVGVPSDGFEQPTA KAITAAGQPTPVAPPTSPIPQLITPTPRERDEGLEEKQKQQQLGEPRSPT ARCILNCCCVIIGFLILVYYKIRNMYNYN	898
Monocot plants				
Oryza sativa	OsSS1	Os03g0401300	MGEAAGDRVLSRLHSVRERIGDSLSAHPNELVAVFTRLVNLGKGMLQ AHQIIAEYNNAISEADREKLKDGAFEDVLRSAQEGIVISPWVALAIRPR PGVWEYVRVNVSELAVELLTVPEYLQFKEQLVEEGTNNNFVLELDFEP FNASFPRPSLSKSIGNGVQFLNRHLSSKLFHDKESMYPLLNFLRAHNYK GMTMMLNDRIRSLSALQGALRKAEEHLSGLSADTPYSEFHHRFQELG LEKGWGDCAKRSQETIHLLLDLLEAPDPSTLEKFLGTIPMVFNVVIMSP HGYFAQANVLGYPDTGGQVVYILDQVRAMENEMLLRIKQQGLNITP RILIVTRLLPDATGTTCGQRLEKVLGTEHTHILRVPFRTENGIVRKWISR FEVWPYLETFTDDVAHEIAGELQANPDLIIGNYSDGNLVACLLAHKM GVTHCTIAHALEKTKYPNSDLYWKKFEDHYHFSCQFTTDLIAMNHAD FIITSTFQEIAGNKDTVGQYESHMAFTMPGLYRVVHGIDVFDPKFNIVS PGADMSIYFPYSESRKRLTSLHPEIEELLYSEVDNNEHKFMLKDRNKPII FSMARLDRVKNLTGLVELYGRNPRLQELVNLVVVCGDHGNPSKDKE EQAEFKKMFDLIEQYNLNGHIRWISAQMNRVRNGELYRYICDTKGAF VQPAFYEAFGLTVVESMTCGLPTFATAYGGPAEIIVNGVSGFHIDPYQG DKASALLVEFFEKCQEDPSHWTKISQGGLQRIEEKYTWKLYSERLMTL TGVYGFWKYVSNLERRETRRYLEMLYALKYRTMASTVPLAVEGEPSN K	816

Table S2. *Cont.*

	OsSS2	Os06g0194900	MAAKLARLHSLRERLGATFSSHPNELIALFSRYVNQGKGMLQRHQLLAEFDALIEADKEKYAPFEDILRAAQEAIVLPPWVALAIRPRPGVWDYIRVNVSELAVEELSVSEYLAFKEQLVDGHTNSNFVLELDFEPFNASFPRPSMSKSIGNGVQFLNRHLSSKLFQDKESLYPLLNFLKAHNHKGTTMMLNDRIQSLRGLQSSLRKAEEYLMGIPQDTPYSEFNHRFQELGLEKGWGDCAKRVLDTIHLLLDLLEAPDPANLEKFLGTIPMMFNVVILSPHGYFAQSNVLGYPDTGGQVVYILDQVRALENEMLLRIKQQGLDITPKILIVTRLLPDAVGTTCGQRVEKVIGTEHTDILRVPFRSENGILRKWISRFDVWPFLETYTEDVANEIMREMQAKPDLIIGNYSDGNLVATLLAHKLGVTQCTIAHALEKTKYPNSDIYLDKFDSQYHFSCQFTADLIAMNHTDFIITSTFQEIAGSKDTVGQYESHIAFTLPGLYRVVHGIDVFDPKFNIVSPGADMSVYFPYTEADKRLTAFHPEIEELLYSEVENDEHKFVLKDKNKPIIFSMARLDRVKNMTGLVEMYGKNAHLRDLANLVIVCGDHGNQSKDREEQAEFKKMYGLIDQYKLKGHIRWISAQMNRVRNGELYRYICDTKGVFVQPAFYEAFGLTVIEAMTCGLPTIATCHGGPAEIIVDGVSGLHIDPYHSDKAADILVNFFEKCKQDSTYWDNISQGGLQRIYEKYTWKLYSERLMTLTGVYGFWKYVSNLERRETRRYIEMFYALKYRSLASAVPLAVDGESTSK	808
	OsSS3	Os07g0616800	MGETTGERALTRLHSMRERIGDSLSAHTNELVAVFSRLVNQGKGMLQPHQIIAEYNAAIPEGEREKLKDSALEDVLRGAQEAIVIPPWIALAIRPRPGVWEYLRINVSQLGVEELSVPEYLQFKEQLVDGSTQNNFVLELDFEPFNASFPRPSLSKSIGNGVQFLNRHLSSKLFHDKESMYPLLNFLRAHNYKGMTMMLNDRIRSLDALQGALRKAEKHLAGITADTPYSEFHHRFQELGLEKGWGDCAQRVRETIHLLLDLLEAPEPSALEKFLGTIPMVFNVVILSPHGYFAQANVLGYPDTGGQVVYILDQVRAMENEMLLRIKQQGLNITPRILIVTRLLPDAHGTTCGQRLEKVLGTEHTHILRVPFRTENGTVRKWISRFEVWPYLETYTDDVAHEISGELQATPDLIIGNYSDGNLVACLLAHKLGVTHCTIAHALEKTKYPNSDLYWKKFEDHYHFSCQFTADLIAMNHADFIITSTFQEIAGNKETVGQYESHMAFTMPGLYRVVHGIDVFDPKFNIVSPGADMSIYFPFTESQKRLTSLHLEIEELLFSDVENTEHKFVLKDKKKPIIFSMARLDHVKNLTGLVELYGRNPRLQELVNLVVVCGDHGKESKDKEEQAEFKKMFNLIEQYNLNGHIRWISAQMNRVRNGELYRYICDMRGAFVQPALYEAFGLTVIEAMTCGLPTFATAYGGPAEIIVHGVSGYHIDPYQNDKASALLVEFFEKCQEDPNHWIKISQGGLQRIEEKYTWKLYSERLMTLSGVYGFWKYVTNLDRRETRRYLEMLYALKYRKMATTVPLAIEGEASTK	816
	OsSS4	Os03g0340500	MSGPKLDRTPSIRDRVEDTLHAHRNELVALLSKYVSQGKGILQPHHILDALDEVQSSGGRALVEGPFLDVLRSAQEAIVLPPFVAIAVRPRPGVWEYVRVNVHELSVEQLTVSEYLRFKEELVDGQYNDPYILELDFEPFNASVPRPNRSSSIGNGVQFLNRHLSSIMFRNKDCLEPLLDFLRGHRHKGHVMMLNDRIQSLGRLQSVLTKAEEHLSKLPADTPYSQFAYKFQEWGLEKGWGDTAGYVLEMIHLLLDVLQAPDPSTLETFLGRIPMIFNVVVVSPHGYFGQANVLGLPDTGGQIVYILDQVRALENEMVLRLKKQGLDFTPKILIVTRLIPEAKGTSCNQRLERISGTQHTYILRVPFRNENGILRKWISRFDVWPYLEKFAEDAAGEIAAELQGTPDFIIGNYSDGNLVASLLSYKMGITQCNIAHALEKTKYPDSDIYWTKYDEKYHFSCQFTADIIAMNNADFIITSTYQEIAGSKNTVGQYESHTAFTLPGLYRIVHGIDVFDPKFNIVSPGADMSIYFPYTEKAKRLTSLHGSLENLISDPEQNDEHIGHLDDRSKPILFSMARLDRVKNITGLVEAYAKNARLRELVNLVVVAGYNDVKKSKDREEIAEIEKMHELIKTYNLFGQFRWISAQTNRARNGELYRYIADTHGAFVQPAFYEAFGLTVVEAMTCGLPTFATVHGGPAEIIEHGISGFHIDPYHPDQAANLIADFFEQCKQDPNHWVEVSNRGLQRIYEKYTWKIYSERLMTLAGVYGFWKYVSKLERRETRRYLEMFYILKFRELAKTVPLAVDEAH	809

Table S2. *Cont.*

	OsSS5	Os04g0309600	MASKLSFKRMDSIAETMPDALRQSRYQMKRCFQRYVSKGKRLLKNQQLMEELEKSLDDKVEKEKLVEGFLGYIICSTQEAVVLPPFVAFAVRMNPGIWEYVKVHSDDLSVEGITPSEYLKFKETLYDEKWAKDDNSLEVDFGALDLSTPHLTLPSSIGNGLQFVSKFMSSKLGGKPESMKPLLDYLLTLNYRGEKLMINDTIDTVSKLQTALLLAEVFVSGLPKYTPYLKFEQRFQEWGLERGWGDTAERCKETLNCLSEVLQAPDPTNMEKFFSRVPSIFNIVIFSIHGYFGQEKVLGLPDTGGQVVYILDQVRAMEEELLQRIKQQGLHVTPKILVLTRLIPDAKGTKCNVELEPVENTKYSHILRVPFKTEDGKDLRQWVSRFDIYPYLERYAQDSCAKILDILEGKPDLIIGNYTDGNLVASLLSNKLCVTQGTIAHALEKTKYEDSDVKWREMDQKYHFSCQFTADMISMNTSDFIITSTYQEIAGSKEKPGQYEHHYAFTMPGLCRYATGINVFDPKFNIAAPGADQSIYFPFTQKQKRLTDLHPQIDELLYSKDDTDEHIGYLADRNKPIIFSMARLDKVKNKKLRDLVNLVVVAGLLDASQSKDREEIEEINKMHNLMDRYQLKGQIRWIKAQTDRVRNGELYRCIADTKGAFVQPALYEAFGLTVIEAMNCGLPTFATNQGGPAEIIIDGVSGFHVNPINDREAGIKIADFFQKCKEDPSYWNKVSTAGLQRICECYTWKIYATRVLNMGSTYSFWKTLNKEERQAKQRYLQIFYNVQYRNLAKAMARAGDQQARQTTTGVAPSEIVVRPKERKPQTRMQRILTRLAGQKPPVSE	844
	OsSS6	Os02g0831500	MAVGLRRSDSIADMMPEALRQSRYQMKRCFQRYVSQGKRLMKRQQLLDELDKSVDDKADKDQLLQGFLGYVISSTQEAAVLPPFVAFAVRMNPGIWEFVKVHSANLSVEQMTPSDYLKNKEALVDDKWGAYDDDSQLEVDFGALDLSTPHLTLPSSIGKGAHLVSRFMSSKLTDNKKPLLDYLLALSHRGDKLMINDILDTVDKLQTALLLAEVYVAGLHPDTNYSEFEQKFQEWGLEKGWGDTAETCKETLSSLSEVLQAPDPINMEKFFSTVPCVFTVVIFSIHGYFGQEKVLGMPDTGGQVVYILDQVRALEDELLQRIKQQGLNATPKILVLTRLIPEAKGTKCNVELEPIENTKHSNILRVPFKTEDGKVLPQWVSRFDIYPYLERYAQDSSVKILEILEGKPDLVIGNYTDGNLVASLLTSKLGVTQGTIAHALEKTKYEDSDIKWRELDHKYHFSCQFTADMIAMNTSDFIIASTYQEIAGSKEKPGQYESHYAFTMPGLCRYATGINVFDPKFNIAAPGADQSVYFPFTQKQKRLTDLHPQIEELLYSKEDNNEHIGHLADRSKPIIFSMARLDKIKNITGLVEWYGQNKRLRDLVNLVIVGGLLDPSQSKDREEIEEINKMHSLINKYQLVGQIRWIKGQTDRVRNGELYRCIADTKGAFVQPALYEAFGLTVIEAMNCGLPTFATNQGGPAEIIVDEVSGFHINPLNGKEASDKIADFFQKCKEDLIYWSKMSTAGLQRIYECYTWQIYATKVLNMASIYGFWRTLDKEERQAKQHYLHMFYNLQFRKLAKNVPTLGEQPAQPTESAEPNRIIPRPKERQVCPFLRNLLKKETGNN	846
Zea mays	ZmSS1	L29418	MGEGAGDRVLSRLHSVRERIGDSLSAHPNELVAVFTRLKNLGKGMLQPHQIIAEYNNAIPEAEREKLKDGAFEDVLRAAQEAIVIPPWVALAIRPRPGVWEYVRVNVSELAVEELRVPEYLQFKEQLVEEGPNNNFVLELDFEPFNASFPRPSLSKSIGNGVQFLNRHLSSKLFHDKESMYPLLNFLRAHNYKGMTMMLNDRIRSLSALQGALRKAEEHLSTLQADTPYSEFHHRFQELGLEKGWGDCAKRAQETIHLLLDLLEAPDPSTLEKFLGTIPMVFNVVILSPHGYFAQANVLGYPDTGGQVVYILDQVRAMENEMLLRIKQCGLDITPKILIVTRLLPDATGTTCGQRLEKVLGTEHCHILRVPFRTENGIVRKWISRFEVWPYLETYTDDVAHEIAGELQANPDLIIGNYSDGNLVACLLAHKMGVTHCTIAHALEKTKYPNSDLYWKKFEDHYHFSCQFTTDLIAMNHADFIITSTFQEIAGNKDTVGQYESHMAFTMPGLYRVVHGIDVFDPKFNIVSPGADLSIYFPYTESHKRLTSLHPEIEELLYSQTENTEHKFVLNDRNKPIIFSMARLDRVKNLTGLVELYGRNKRLQELVNLVVVCGDHGNPSKDKEEQAEFKKMFDLIEQYNLNGHIRWISAQMNRVRNGELYRYICDTKGAFVQPAFYEAFGLTVVEAMTCGLPTFATAYGGPAEIIVHGVSGYHIDPYQGDKASALLVDFF	816

Table S2. *Cont.*

			DKCQAEPSHWSKISQGGLQRIEEKYTWKLYSERLMTLTGVYGFWKYVS NLERRETRRYLEMLYALKYRTMASTVPVAVEGEPSSK	
	ZmSS2	L22296	MGEGAGDRVLSRLHSVRERIGDSLSAHPNELVAVFTRLKNLGKGMLQP HQIIAEYNNAIPEAEREKLKDGAFEDVLRAAQEAIVIPPWVALAIRPRPG VWEYVRVNVSELAVEELRVPEYLQFKEQLVEEGPNNNFVLELDFEPFN ASFPRPSLSKSIGNGVQFLNRHLSSKLFHDKESMYPLLNFLRAHNYKGM TMMLNDRIRSLSALQGALRKAEEHLSTLQADTPYSEFHHRFQELGLEK GWGDCAKRAQETIHLLLDLLEAPDPSTLEKFLGTIPMVFNVVILSPHGY FAQANVLGYPDTGGQVVYILDQVRAMENEMLLRIKQCGLDITPKILIVT RLLPDATGTTCGQRLEKVLGTEHCHILRVPFRTENGIVRKWISRFEVWP YLETYTDDVAHEIAGELQANPDLIIGNYSDGNLVACLLAHKMGVTHCT IAHALEKTKYPNSDLYWKKFEDHYHFSCQFTTDLIAMNHADFIITSTFQ EIAGNKDTVGQYESHMAFTMPGLYRVVHGIDVFDPKFNIVSPGADLSIY FPYTESHKRLTSLHPEIEELLYSQTENTEHKFVLNDRNKPIIFSMARLDRV KNLTGLVELYGRNKRLQELVNLVVVCGDHGNPSKDKEEQAEFKKMFD LIEQYNLNGHIRWISAQMNRVRNGELYRYICDTKGAFVQPAFYEAFGL TVVEAMTCGLPTFATAYGGPAEIIVHGVSGYHIDPYQGDKASALLVDFF DKCQAEPSHWSKISQGGLQRIEEKYTWKLYSERLMTLTGVYGFWKYVS NLERRETRRYLEMLYALKYRTMASTVPLAVEGEPSSK	816
	ZmSS3	BT069288	MGEGAGDRVLSRLHSVRERIGDSLSAHPNELVAVFTRLKNLGKGMLQP HQIIAEYNNAIPEAEREKLKDGAFEDVLRAAQEAIVIPPWVALAIRPRPG VWEYVRVNVSELAVEELRVPEYLQFKEQLVEEGPNNNFVLELDFEPFN ASFPRPSLSKSIGNGVQFLNRHLSSKLFHDKESMYPLLNFLRAHNYKGM TMMLNDRIRSLSALQGALRKAEEHLSTLQADTPYSEFHHRFQELGLEK GWGDCAKRAQETIHLLLDLLEAPDPSTLEKFLGTIPMVFNVVILSPHGY FAQANVLGYPDTGGQVVYILDQVRAMENEMLLRIKQCGLDITPKILIVT RLLPDATGTTCGQRLEKVLGTEHCHILRVPFRTENGIVRKWISRFEVWP YLETYTDDVAHEIAGELQANPDLIIGNYSDGNLVACLLAHKMGVTHCT IAHALEKTKYPNSDLYWKKFEDHYHFSCQFTTDLIAMNHADFIITSTFQ EIAGNKDTVGQYESHMAFTMPGLYRVVHGIDVFDPKFNIVSPGADLSIY FPYTESHKRLTSLHPEIEELLYSQTENTEHKFVLNDRNKPIIFSMARLDRV KNLTGLVELYGRNKRLQELVNLVVVCGDHGNPSKDKEEQAEFKKMFD LIEQYNLNGHIRWISAQMNRVRNGELYRYICDTKGAFVQPAFYEAFGL TVVEAMTCGLPTFATAYGGPAEIIVHGVSGYHIDPYQGDKASALLVDFF DKCQADPSHWSKISQGGLQRIEEKYTWKLYSERLMTLTGVYGFWKYVS NLERRETRRYLEMLYALKYRTMASTVPLAVEGEPSSK	816
	ZmSS4	NM_001111 941	MAAKLTRLHSLRERLGATFSSHPNELIALFSRYVHQGKGMLQRHQLLA EFDALFDSDKEKYAPFEDILRAAQEAIVLPPWVALAIRPRPGVWDYIRV NVSELAVEELSVSEYLAFKEQLVDGQSNSNFVLELDFEPFNASFPRPSMS KSIGNGVQFLNRHLSSKLFQDKESLYPLLNFLKAHNYKGTTMMLNDRI QSLRGLQSSLRKAEEYLLSVPQDTPYSEFNHRFQELGLEKGWGDTAKRV LDTLHLLLDLLEAPDPANLEKFLGTIPMMFNVVILSPHGYFAQSNVLGY PDTGGQVVYILDQVRALENEMLLRIKQQGLDITPKILIVTRLLPDAAGTT CGQRLEKVIGTEHTDIIRVPFRNENGILRKWISRFDVWPYLETYTEDVSSE IMKEMQAKPDLIIGNYSDGNLVATLLAHKLGVTQCTIAHALEKTKYPN SDIYLDKFDSQYHFSCQFTADLIAMNHTDFIITSTFQEIAGSKDTVGQYES HIAFTLPGLYRVVHGIDVFDPKFNIVSPGADMSVYYPYTETDKRLTAFH PEIEELIYSDVENSEHKFVLKDKKKPIIFSMARLDRVKNMTGLVEMYGK NARLRELANLVIVAGDHGKESKDREEQAEFKKMYSLIDEYKLKGHIRW ISAQMNRVRNGELYRYICDTKGAFVQPAFYEAFGLTVIESMTCGLPTIAT CHGGPAEIIVDGVSGLHIDPYHSDKAADILVNFFDKCKADPSYWDEISQ	802

Table S2. *Cont.*

			GGLQRIYEKYTWKLYSERLMTLTGVYGFWKYVSNLERRETRRYIEMFYA<br>LKYRSLASQVPLSFD	
	ZmSS5	EU971052	MAAKLTRLHSLRERLGATFSSHPNELIALFSRYVHQGKGMLQRHQLLA<br>EFDALFDSDKEKYAPFEDILRAAQEAIVLPPWVALAIRPRPGVWDYIRV<br>NVSELAVEELSVSEYLAFKEQLVDGQSNSNFVLELDFEPFNASFPRPSMS<br>KSIGNGVQFLNRHLSSKLFQDKESLYPLLNFLKAHNYKGTTMMLNDRI<br>QSLRGLQSSLRKAEEYLLSVPQDTPYSEFNHRFQELGLEKGWGDTAKRV<br>LDTLHLLLDLLEAPDPANLEKFLGTIPMMFNVVILSPHGYFAQSNVLGY<br>PDTGGQVVYILDQVRALENEMLLRIKQQGLDITPKILIVTRLLPDAAGTT<br>CGQRLEKVIGTEHTDIIRVPFRNENGILRKWISRFDVWPYLETYTEDVSSE<br>IMKEMQAKPDLIIGNYSDGNLVATLLAHKLGVTQCTIAHALEKTKYPN<br>SDIYLDKFDSQYHFSCQFTADLIAMNHTDFIITSTFQEIAGSKDTVGQYES<br>HIAFTLPGLYRVVHGIDVFDPKFNIVSPGADMSVYYPYTETDKRLTAFH<br>PEIEELINSDVENSEHKFVLKDKKKPIIFSMARLDRVKNMTGLVEMYGK<br>NARLRELANLVIVAGDHGKESKDREEQAEFKKMYSLIDEYKLKGHIRW<br>ISAQMNRVRNGELYRYICDTKGAFVQPAFYEAFGLTVIESMTCGLPTIAT<br>CHGGPAEIIVDGVSGLHIDPYHSDKAADILVNFFDKCKADPSYWDKISQ<br>GGLQRIYEKYTWKLYSERLMTLTGVYGFWKYVSNLERRETRRYIEMFYA<br>LKYRSLASQVPLSFD	802
Triticum aestivum	TaSS1	AJ001117	MAAKLTRLHSLRERLGATFSSHPNELIALFSRYVHQGKGMLQRHQLLA<br>EFDALFESDKEKYAPFEDILRAAQEAIVLPPWVALAIRPRPGVWDYIRV<br>NVSELAVEELTVSEYLAFKEQLVDEHASSKFVLELDFEPFNASFPRPSMS<br>NSIGKRVQFLNRHLSSQLFQDKESLYPLLNFLKAHYYKGTTMMLNDRI<br>QSLRGLQSALRKAEEYLVSIPEDTPSSEFNHRFQELGLEKGWGDTAKRV<br>HDTIHLLLDLLEAPDPASLEKFLGTIPMMFNVVILSPHGYFAQSNVLGY<br>PDTGGQVVYILDQVRALENEMLLRIKQQGLDITPKILIVTRLLPDAVGTT<br>CGQRLEKVIGTEHTDILRVPFRTDNGILRKWISRFDVWPYLETYTEDVA<br>NELMREMQTKPDFIIGNNSDGNLVATLLAHKLGVTQCTIAHALEKTKY<br>PNSDIYLDKFDSQYHFSCQFTADLIAMNHTDFIITSTFQEIAGSKDSVGQ<br>YESHIAFTLPDLYRVVHGIDVFDPKFNIVPGADMTVYFPYTETDKRLTAF<br>HSEIEELLYSDVENDEHKFVLKDRNKPIIFSMARLDRVKNMTGLVEMYG<br>KNAHLKGFGKLVIVAGDHGKESKDREEQAEFKRMYSLIEEYKLKGHIR<br>WISAQMNRVRNGELYRYICDTKGAFVQPAFYEAFGLTVIEVHECGLPTI<br>ATCHGGPAEIIVNGVSGLHIDPYHSDKAADILVNFFEKCSEDPSYWDK<br>MSEGGLKRIYEKYTWKLYSERLMTLTGVYGFWKYVSNLERRETRRYLE<br>MFYALKYRSLAAAVPLAVDGESSDN	807
	TaSS2	AJ000153	MGETAGGRALSRIHSVRERIGDSLSAHTNELVAVFSRLVNQGKGMLQP<br>HQITAEYNAAIPEAEREKLKDTAFEDLLRGAQEAIVIPPWVALAIRPRPG<br>VWEYVRVNVSELGVEELNVLNYLQFKEQLANGSIDNNFVLELDFEPFN<br>ASFPRPSLSKSIGNGVQFLNRHLSSKLFHDKESMYPLLNFLRAHNYKGM<br>TMMLNDRIRSLGTLQGALRKAETHLSGLPADTPYSEFHHRFQELGLEK<br>GWGDCAQRASETIHLLLDLLEAPDPSSLEKFLGTIPMVFNVVILSPHGYF<br>AQANVLGYPDTGGQVVYILDQVRAMENEMLLRIKQQGLDITPKILIVT<br>RLLPDAHGTTCGQRLEKVLGTEHTHILRVPFKTEDGIVRKWISRFEVWP<br>YLEAYTDDVAHEIAGELQATPDLIIGNYSDGNLVACSLAHKLGVTHCIA<br>HALEKTKYPNSDLYWKKFEDHYHFSCQFTADLIAMNHADFIITSTFQEI<br>AGNKDTVGQYESHMAFTMPSLYRVVHGIDVFDPKFNIVSPGADMSIYF<br>PYTEQQKRLTSLHTEIEELLFSDVENAEHKFVLKDKKKPIIFSMARLDRV<br>KNMTGLVEMYGRNPRLQELVNLVVVCGDHGKVSKDKEEQAEFKKMF<br>DLIEQYNLIGHIRWISAQMNRVRNGELYRYICDMKGAFVQPAFYEAFG<br>LTVIEAMTCGLPTFATAYGGPAEIIVHGVSGYHIDPYQNDKASALLVDF	815

			FGKCQEDPSHWNKISQGGLQRIEEKYTWKLYSERLMTLSGVYRFWKYV<br>SNLDRRETRRYLEMLYALKYRKMAATVPLAVEGETSGK	
Hordeum vulgare	HvSS1	X69931	MGETAGERALSRVHSVRERIGHSLSAHTNELVAVFSRLVNQGKGMLQP<br>HQITAEYNAAIPEAEREKLKNTPFEDLLRGAQEAIVIPPWVALAIRPRPG<br>VWEYVRVNVSELGVEELSVLRYLQFKEQLANGSTDNNFVLELDFGPFN<br>ASFPRPSLSKSIGNGVQFLNRHLSSKLFHDKESMYPLLNFLRAHNYKGM<br>TMMLNDRIRSLGTLQGALRKAETHLSGLPADTPYTEFHHRFQELGLEK<br>GWGDCAQRASETIHLLLDLLEAPDPSSLEKFLGTIPMVLNVVILSPHGYF<br>AQANVLGYPDTGGQVVYILDQVRAMENEMLLRIKQQGLDITPKILIVT<br>RMLPDAHGTTCGQRLEKVLGTEHTHILRVPFKTEDGIVRKWISRFEVWP<br>YLEAYTDDVAHEIAGELQANPDLIIGNYSDGNLVACLLAHKLGVTHCT<br>IAHALEKTKYPNSDLYWKKFEDHYHFSCQFTADLIAMNHADFIITSTFQ<br>EIAGNKDTVGQYESHMAFTMPGLYRVVHGIDVFDPKFNIVSPGADMSI<br>YFPYTEQQKRLTSLHTEIEELLFSDVENAEHKFVLKDKKKPIIFSMARLD<br>RVKNMTGLVEMYGRNPRLQELVNLVVVCGDHGKVSKDKEEQVEFKK<br>MFDLIEKYNLSGHIRWISAQMNRVRNGELYRYICDMKGAFVQPAFYEA<br>FGLTVIEAMTCGLPTFATAYGGPAEIIVNGVSGYHIDPYQNDKASALLV<br>GFFGKCQEDPSHWNKISQGGLQRIEEKYTWKLYSERLMTLSGVYGFWK<br>YVSNLDRRETRRYLEMLYALKYRKMAATVPLAVEGETSGE	816
	HvSS2	Y15802	MGETAGERALSRVHSVRERIGHSLSAHTNELVAVFSRLVNQGKGMLQP<br>HQITAEYNAAIPEAEREKLKNTPFEDLLRGAQEAIVIPPWVALAIRPRPG<br>VWEYVRVNVSELGVEELSVLRYLQFKEQLANGRTDNNFVLELDFGPFN<br>ASFPRPSLSKSIGNGVQFLNRHLSSKLFHDKESMYPLLNFLRAHNYKGM<br>AMMLNDRIRSLGTLQGALRKAETHLSGLPADTPYSEFHHRFQELGLEK<br>GWGDCAQRASETIHLLLDLLEAPDPSSLEKFLGTIPMVFNVVILSPHGYF<br>AQANVLGYPDTGGQVVYILDQVRAMENEMLLRIKQQGLDITPKILIVT<br>RMLPDAHGTTCGQRLEKVLGTEHTHILRVPFKTEDGIVRKWISRFEVWP<br>YLEAYTDDVAHEIAGELQANPDLIIGNYSDGNLVACLLAHKLGVTHGT<br>IAHALEKTKYPNSDLYWKKFEDHYHFSCQFTADLIAMNHADFIITSTFQ<br>EIAGKKDTVGQYESHMAFTMPGLYRVVHGIDVFDPKFNIVSPGADMSI<br>YFPYTEQQKRLTSLHTEIEELLFSDVENAEHKFVLKDKKKPIIFSMARLD<br>RVKNMTGLVEMYGRNPRLQELVNLVVVCGDHGKVSKDKEEQVEFKK<br>MFDLIEKYNLSGHIRWISAQMNRVRNGELYRYICDMKGAFVQAAFYEA<br>FGLTVIEAMTCGLPTFATAYGGPAEIIVNGVSGYHIDPYQNDKASALLV<br>DFFGKCQEDPSHWNKISQGGLQRIEEKYTWKLYSERLMTLSGVYGFWK<br>YVSNLDRRETRRTLKCSMPSSTAKWLQLSHWLLRARPRANDLSLPEK	823
Bambusa oldhamii	BoSS1	AF412036	MGEAAGDRVLSRLHSVRERIGDSLSAHPNELVAVFTRLVNLGKGMLQP<br>HQIIAEYNNAIPEAEREKLKDGAFEDVLRAAQEAIVIPPWVALAIRPRPG<br>VWEYVRVNVSELAVEELRVPEYLQFKEQLVEGSTNNNFVLELDLEPFN<br>ASFPRPSLSKSIGNGVQFLNRHLSSKLFHDKESMYPLLNFLRAHNYKGM<br>TMMLNDRIRSLSALQGALRKAEEHLSGLSADTSYSDFHHRFQELGLEK<br>GWGDCAKRAQETIHLLLDLLEAPDPSTLEKFLGTIPMVFNVVILSPHGY<br>FAQANVLGYPDTGGQVVYILDQVRAMENEMLLRIKQQGLNITPRILIVT<br>RLLPDATGTTCGQRLEKVLGTEHTHILRVPFRTENGIVRKWISRFEVWP<br>YLETFTDDVAHEIAGELQANPDLIIGNYSDGNLVACLLAHKMGVTHCT<br>IAHALEKTKYPNSDLYWKKFEDHYHFSCQFTTDLIAMNHADFIITSTFQ<br>EIAGNKDTVGQYESHMAFTMPGLYRVVHGIDVFDPKFNIVSPGADLSIY<br>FPYTESHKRLTSLHPEIEELLYSDVDNHEHKFVLKDRNKPIIFSMARLDR<br>VKNLTGLVELYGRNPRLQELVNLVVVCGDHGNPSKDKEEQAEFQKMF<br>DLIEQYNLNGHIRWISAQMNRVRNGELYRYICDTKGAFVQPAFYEAFG<br>LTVVESMTCGLPTFATAYGGPAEIIVHGVSGFHIDPYQGDKASALLVEFF<br>EKCQQDHSHWTKISQGGLQRIEEKYTWKLYSERLMTLTGVYGFWKYVS<br>NLERRETRRYLEMLYALKYRTMASTVPLAVDGEPSSK	816

Table S2. *Cont.*

	BoSS2	AF412038	MAAKLTRLHSLRERLSATFSSHPNELIALFSRYVHQGKGMLQRHQLLA<br>EFDALIAADKEKYAPFEDILRAAQEAIVLPPWVALAIRPRPGVWDYIRV<br>NVSELAVEELSVSEYLEFKEQLVDGHTNSNFVLELDFEPFNASFPRPSMS<br>KSIGNGVQFLNRHLSSKLFQDKESLYPLLNFLKAHNHKGTTMMLNDRI<br>QSLRGLQSALRKAEEYLMSFPQDTPYSEFNHRFQELGLEKGWGDTAKR<br>VLDTIHLLLDLLEAPDPANLEKFLGTIPMTFNVVILSPHGYFAQSNVLGY<br>PDTGGQVVYILDQVRALENEMLLRIKQQGLDITPKILIVTRLLPDAVGTT<br>CGQRLEKVIGTEHTDILRVPFRTENGILRKWISRFDVWPFLETYTEDVAN<br>EIMREMQAKPDLIIGNYSDGNLVATLLAHKLGVTQCTIAHALEKTKYP<br>NSDIYLDKFDSQYHFSCQFTADLIAMNHTDFIITSTFQEIAGSKDTVGQY<br>ESHIAFTLPGLYRVVHGIDVFDPKFNIVSPGADMSVYFPYTETDKRLTAF<br>HPEIEELIYSDVENSEHKFVLKDKNKPIIFSMARLDRVKNMTGLVEMYG<br>KNAHLRDLANLVIVAGDHGKESKDREEQAEFKRMYSLIDEYKLKGHIR<br>WISAQMNRVRNGELYRYICDTKGVFVQPAFYEAFGLTVIESMTCGLPTI<br>ATCHGGPAEIIVDGVSGLHIDPYHSNKAADILVNFFEKCKEDPTYWDKI<br>SQGGLKRIYEKYTWKLYSERLMTLTGVYGFWKYVSNLERRETRRYLEMF<br>YALKYRSLASAVPLAVDGDSAAN	808
	BoSS3	AF412037	MGETAGDRVLSRLHSVRERIGDSLSAHPNELVAVFTRLVNLGKGMLQP<br>HQIIAEYNNAIPEAERDKLKDGAFEDVLRAAQEAIVIPPWVALAIRPRP<br>GVWEYVRVNVSELAVEELRVPEYLQFKEQLVEGSTNNNFVLELDFEPF<br>NASFPRPSLSKSIGNGVQFLNRHLSSKLFHDKESMYPLLNFLRAHNYKG<br>MTMMLNDRIRSLSALQGALRKAEEHLSGLSADTPYSDFHHRFQELGLE<br>KGWGDCAKRAQETIHLLLDLLEAPDPSTLEKFLGTIPMVFNVVILSPHG<br>YFAQANVLGYPDTGGQVVYILDQVRAMENEMLLRIKQQGLNITPRILIV<br>TRLLPDATGTTCGQRLEKVLGTEHTHILRVPFRTENGIVRKWISRFEVWP<br>YLETFTDDVAHEIAGELQANPDLIIGNYSDGNLVACLLAHKMGVTHCT<br>IAHALEKTKYPNSDLYWKKFEDHYHFSCQFTTDLIAMNHADFIITSTFQ<br>EIAGNKDTVGQYESHMAFTMPGLYRVVHGIDVFDPKFNIVSPGADMSI<br>YFPYSESHKRLTSLHPEIEELLYSDVDNNEHKFVLKDRNKPIIFSMARLD<br>RVKNLIGLVELYGRNPRLQELVNLVVVCGDHGNPSKDKEEQAEFKKM<br>FDHIEQYNLNGHIRWISAQMNRVRNGELYRYICDTRGAFVQPAFYEAF<br>GLTVVESMSCGLPTFATAYGGPAEIIVHGVSGFHIDPYQGDKASALLVE<br>FFEKCQQDPAHWTKISQGGLQRIEEKYTWKLYSERLMTLTGVYGFWKY<br>VSNLERRETRRYLEMLYALKYRKMASTVPLAVEGEPSNK	816
	BoSS4	AF412039	MAAKLTRLHSLRERLGATFSSHPNELIALFSRYVNQGKGMLQRHQLLA<br>EFDALMDADKEKYAPFEDILRAAQEAIVLPPWVALAIRPRPGVWDYIR<br>VNVSELAVEELSVSEYLAFKEQLVDGHTNSNFVLELDFEPFNASFPRPS<br>MSKSIGNGVQFLNRHLSSKLFQDKESLYPLLNFLKAHNPKGKTMMLN<br>DRIQSLRGLQSALRKAEEYLISIPQDTPCSEFNHRFQELGLEKGWGDTAK<br>RVLDTIHLLLDLLEAPDPANLEKFLGTIPMTFNVVILSPHGYFAQSNVLG<br>YPDTGGQVVYILDQVRALENEMLLRIKQQGLDITPKILIVTRLLPDAVGT<br>TCGQRLEKVLGTEHTDILRVPFRTENGILRKWISRFDVWPFLETYTEDVA<br>NEIMREMQAKPDLIIGNYSDGNLVATLLAHKLGVTQCTIAHALEKTKY<br>PNSDIYLDKFDSQYHFSCQFTADLIAMNHTDFIITSTFQEIAGSKDTVGQ<br>YESHIAFTLSGLYRVVHGIDVFDPKFNIVSPGADMSVYFPYTETDKRLTA<br>FHPEIEELIYSDVENSEHQFVLKDKNKPIIFSMARLDRVKNMTGLVEMY<br>GKNAHLRDLANLVVVAGDHGKESKDREEQAEFKRMYSLIEEYKLKGH<br>IRWISAQMNRVRNGELYRYICDTRGVFVQPAFYEAFGLTVIESMTCGLP<br>TIATCHGGPAEIIVDGVSGLHIDPYHSDKAADILVNFFEKCKEDPTYWD<br>KISLGGLKRIYEKYTWKLYSERLMTLTGVYGFWKYVSNLERRETRRYLE<br>MFYALKYRSLASAVPLAVDGDSVAK	808

Table S2. *Cont.*

B. Bacteria				
Nostoc punctiforme	NpSSA	AJ316589	MSELLQAVLDSEERSDLRSFLSELRQQEKKYLLRNDILNVYSEYCSKSQK<br>PEAYYTTSELGKLIYYTQEIIQEDSNFCFIIRSKIASQEVYWLTSDLSIEPMT<br>VQDLLDLRDRLVNKFHPNDGDLLELDFGPFYDYTPIIRDPKNIGKGVQF<br>LNRYLSSKIFQDSKQLLENLLNFLRLHHYNGVQLLVNDRIQSQQQLSEQ<br>VKKAIGFVNNRPDDEPYEQFRFQLQSMGFEPGWGNTAARVRETLNILD<br>ELIDSADPQTLEAFISRVPMIFRIVLVSAHGWFGQEGVLGRPDTGGQVV<br>YVLDQAKSLEKQLQEDVLLAGLEKLNVEPKVIILTRLIPNSDGTLCNQR<br>LEKVHGTENAWILRVPLRDFNPNMTQNWISRFEFWPYLETFAIDSEREL<br>RAEFQGTPDLIVGNYTDGNLIAFLLARRLKVTQCNVAHALEKSKYLFSN<br>LYWQELEEKYHFSLQFTADLIAMNAANFVVSSTYQEIVGTPDSVGQYES<br>YKCFTMPELYHVTNGIELFSPKFNVVPPGVNENNYFPYTRTKDRVESDR<br>QRLAETLFTLEDPTQIFGKLDDPNKRPLFSMARLDHIKNLTGLAECYGQ<br>SKELQEHCNLILVAGKLRVEESGDNEERDEIIKLYNIIDEYNLHGKIRWL<br>GVRLSKTDSGEIYRVIADRQGIFVQPALFEAFGLTILESMVSGLPTFATQF<br>GGPLEIIQDKVNGFLINPTNLDETATKIVDFITKCEQNPNYWNEISQRGI<br>DRVYSTYTWKIHTSKLLSLARIYGFWNFTSKENREDLLRYLEALFYLIYK<br>PRAQQLLEQHKYR	806
Lyngbya majuscule	LmSS	AY522504	LIQDVLESDEKSDLRHFTSQLKTAEPRYLLRNEILAAFNEYCTKHKKSEY<br>FYHSSHLGKLIYYTQEIILEDESLCLIIRPKIAAKRAFRLFEDLRAQEVTPEE<br>LLDIRDRFVNRYNPKVGEVLQLDFQPFYDYSPLIRDPKNIGKGVRFLNR<br>YLSSKLFQDPEQWLESLYGFLKVRHFHGNQLLINERIHNHQQLSEQVKL<br>ALEFVSDRPDSESYDKFRFKLQEMGFEPGWGNTASRVRETLAMLDELID<br>EPDDRALEQFLSRIPMIFRIVLVSVHGWFGQEGVLGRPDTGGQVVYVLD<br>QAKSLEKQLQENLTLAGLNIQPKVIILTRLIPNNDGTRCNERLEKIKGTE<br>NAWILRVPFREFNPKVTQDWISRFEIWPYLETYAIDAEKELLAEFQGRPD<br>LIVGNYSDGNLVAFLLARRLKVTQCNIAHALEKSKYLFSNLYWQDSEQ<br>QYHFSLQFTADLIAMNAANFIISSTYQEIVGTADSVGQYESYQNFTMPD<br>LYHVINGIELFSPKFNVVPPGVNETVFFPYTRTQGRVASDIKRLDEFLFTL<br>DDEAQVFGKLDYPNKRPIFSMARLDRIKNLTGLAECFGKSRKLQERCN<br>LILIAGNLRTEDSSDSEEKAEIIKLYQIIEEYNLYGKIRWLGVRLSKSDSGE<br>VYRVIADRHGIFVQPALFEAFGLTILESMISGLPTFGTQFGGPLEIIQDKV<br>NGILINPTNQEEMAQKILDFVTKCEENPQYWEEISNQGIERVYSTYTWKI<br>HTTRLLSLARIYRFWNYTSKANQEDMLRYLEALFHLIYKPRAKKLLEEH<br>LTLPARA	804
Thermosynechococcus elongates	TeSS	NP_681838	MTCVLLKAVVESDERADLRQFSRILQLGEKRYLLRNDILDAFADYCRD<br>QERPVPPPSESRLSKLVFYTQEIIVDNESLCWIVRPRIAQQEVCRLLVEDL<br>TIVPMTIPELLDLRDRLVNHYHPNEGDVFEIDVQPFYDYSPIIRDAKNIG<br>KGVEFLNRYLSSKLFQDPRQWQQNLFNFLRIHRYNGYQLLINERIRSPQ<br>HLSEQVKQALVVLSDRPPTEAYSEFRFELQNLGFEPGWGNTVARVRDT<br>LEILDQLLDSPDHQVLEAFVSRIPMLFRIALISPHGWFGQEGVLGRPDTG<br>GQVVYILDQVKSLEKQMREDLELAGLGVLEAQPKIIVLTRLIPNAEGTL<br>CNQRLEKIYGTNDAWILRVPFREFNPKVTQNWISRFEIWPYLETFAIDAE<br>RELRAEFGHVPDLIIGNYSDGNLVAFLLARRLKVTQCNIAHALEKSKYL<br>FSNLYWQDLEDKYHFSLQFTADLIAMNAANFIISSTYQEIVGTPDSIGQY<br>ESYQSFTMPDLYHVVNGIELFSPKFNVVPPGVNEQVYFPYYHYTERLEG<br>DRQRLEELLFTLEDPQQIYGYLEAPEKRPLFSMARLDRIKNLTGLAEAFG<br>RSKALQERCNLILVAGKLRTADSSDREEIAEIEKLYQIIHQYNLHGKIRW<br>LGIRLPKADSGEIYRIIADRQGIFVQPALFEAFGLTILEAMISGLPTFGTRF<br>GGPLEIIQDGVNGFYINPTHLEEMAETIVRFLEACDRDPQEWQRISKAGI	808

Table S2. *Cont.*

			ERVYSTYTWKIHCTRLLSLAKIYGFWNFSSQENREDMMRYMEALFHLL YKPRAQALLAEHLQR	
Acaryochloris marina	AmSS	NC_009925	MSQLIQAVLNSDEKTDLRQFASEVHNQPERYLLRNDILSVFDTFCEKYE KTPAFQLSSRLQKLIYYTQEILVEDENLYLIIRPKIASEEAYRLDPRELVYE QIQIDELLDLRDRFVGHYHPQEGDILEIDFRPFYDYSPVIRDPKNIGRGV QYLNRYLSSKMFEGPQQWLFSLFSFLKLHSYNGTQLLINQRIQSPEHLSE CVKQAISLVGGLPPEQPYPEFRFNFQELGFEPGWGNTAARVLETLEMLD ELIDSPDDQVLEAFISRIPMIFKIVLVSIHGYFGQEGVLGRPDTGGQVVYV LDQAKSLEKQLQEDLQFAGLDTLEVQPKLIILSRLIPNSEGTLCNQRLEK VHATDNVWILRVPFRELNPKYTQNWISRFEIWPYLETYAIDAERELLAE FRGLPDLIVGNYTDGNLVAFLLSRRLGVTQCNVAHALEKSKYLFSNLY WQDLEEQYHFSMQFTADLIAMNAANFIVTSTYQEIAGRPDTIGQYESYR SFTMPDLYHVVYGAELFSPKFNVVPPGVNESVYFPFTRHQERTPGDIDR LEELLFTLEDPEHVFGHLDNPGKPPLFSMARLDRIKNLTGLAECFGQHP QLQDHYNLILVAGKLRTSDSVDHEEINEIERLYRIIDQYNLHGKIRWLG VRFPKQDSGEIYRVVADHKGIFVQPALFEAFGLTVLEAMISGLPTFATRF GGPLEIIRDGIDGFYINPTHNEEIATKLLEFAKECATNPDYWQQISEQAIE RVYTTYTWKIHTSRLLSLAKIYGFWNYTSREKREDMLRYIETIFYLLYKP MAKKLLAKHMDS	807

Table S3. The Ka, Ks and Ka/Ks values of PbSSs duplication gene pairs.

Duplicate genes IDs	ks	ka	Ka/ks	Purifying selection	Duplicated type
PbSS1 vs *PbSS18*	0.2407	0.0403	0.167428	Yes	Segmental
PbSS3 vs *PbSS16*	0.1352	0.0201	0.148669	Yes	Segmental
PbSS3 vs *PbSS17*	0.1308	0.0202	0.154434	Yes	Segmental
PbSS3 vs *PbSS15*	0.8614	0.1627	0.188879	Yes	Segmental
PbSS5 vs *PbSS13*	0.543	0.4424	0.814733	Yes	Segmental
PbSS8 vs *PbSS15*	0.1048	0.0191	0.182252	Yes	Segmental
PbSS10 vs *PbSS20*	0.2181	0.0407	0.186612	Yes	Segmental
PbSS12 vs *PbSS7*	0.4387	0.3837	0.87463	Yes	Segmental
PbSS15 vs *PbSS16*	0.7509	0.1816	0.241843	Yes	Tandem
PbSS25 vs *PbSS1*	0.0112	0.005	0.446429	Yes	Segmental
PbSS25 vs *PbSS18*	0.2193	0.0505	0.230278	Yes	Segmental
PbSS28 vs *PbSS3*	0.8743	0.1751	0.200275	Yes	Segmental
PbSS29 vs *PbSS7*	0.0051	0.0041	0.803922	Yes	Segmental
